# Challenging cross couplings, in water, aided by *in situ* iodination of (hetero)aromatic bromides†

**DOI:** 10.1039/d3sc04199a

**Published:** 2023-11-07

**Authors:** Rohan M. Thomas, David B. Obbard, Bruce H. Lipshutz

**Affiliations:** a Department of Chemistry and Biochemistry, University of California Santa Barbara CA 93106 USA lipshutz@chem.ucsb.edu

## Abstract

Palladium-catalyzed reactions that involve functionalized substrates are oftentimes problematic. Those involving aryl or heteroaryl bromides that are either resistant to, or inefficient in such couplings present challenges that are difficult to overcome and may require development of an entirely new route, or worse, no opportunity to install the desired group using a standard coupling strategy. In this report, we describe a solution that allows for the *in situ* conversion of such bromo educts to transient iodide derivatives that can be made and used under environmentally responsible conditions, for subsequent reactions to highly functionalized, complex targets.

## Abstract 2

Palladium-catalyzed reactions that involve functionalized substrates are oftentimes problematic. An initial Br-to-I conversion followed by an immediate cross coupling may allow for the targeted C–C bonds to be obtained.

## Graphical abstract

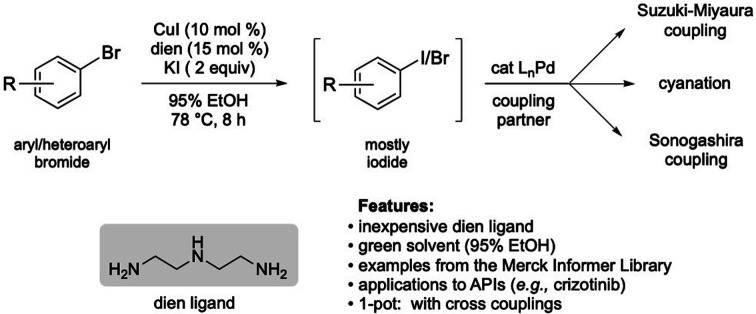

## Introduction

Cross-coupling chemistry has evolved to such an extent that most aryl-/hetero-aryl chlorides, and more often, bromides, are common reaction partners en route to strategically valuable C–C bonds. Nonetheless, as the level of substrate complexity increases the situation can change dramatically, and not for the better. This is why inclusion of reaction partners in studies aimed at developing cross coupling methodology that address late-stage functionalization is an important indicator of potential success. However, what are the options when neither a chloride, or even bromide undergo the desired coupling involving highly derivatized molecules, perhaps due to undesired metal complexation by functionality present that significantly reduces catalyst activity, thereby disabling the initial oxidative addition required to just begin the catalytic cycle. One clear cut example of this was reported by Buchwald, Cernak, and co-workers where a stoichiometric level of palladium was required to form the Pd(ii) adduct in order to subsequently negotiate eventual product formation.^1^

**Fig. 1:**
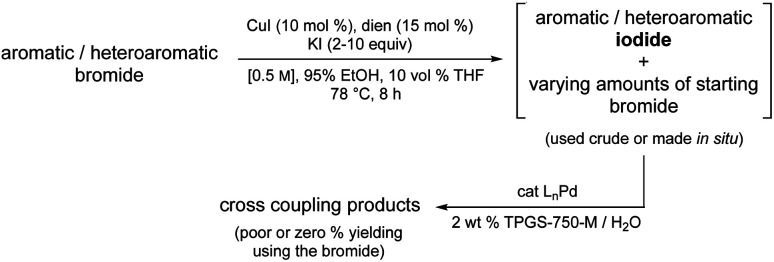
Overall strategy.

Rather than relying on costly and unsustainable approaches, the option to transform, *in situ*, a readily available but recalcitrant bromide precursor to the corresponding iodide under environmentally responsible conditions using a recyclable medium and earth-abundant metal/ligand combination, followed by the immediate Pd-catalyzed coupling of the derived iodide appeared as a potential solution to this longstanding synthetic problem in catalysis. That is, other than pre-forming a stoichiometric adduct with palladium, there is no existing technology that addresses this issue. Indeed, given the situation today, one is faced with the realization that either the targeted adduct from a cross coupling is simply unattainable, or an alternative route must be first devised and then developed. Neither is appealing. In this report, therefore, a simple solution is disclosed that can be utilized immediately; one that relies on commercially available reagents including a known ligand^2^ normally considered a waste product that can be re-purposed, thereby adding yet another element to the overall “greenness” of this technology (Fig. 1).

Just the conversion of halide precursors to the corresponding iodides as coupling partners is far from a new, although strategies along these lines are typically viewed as unattractive; indeed, since the original disclosure over two decades ago,^3^ only two reports using this approach have appeared.^4–6^ This is, perhaps, not surprising, as the conditions required (*vide infra*) are especially harsh and far from environmentally attractive as in all known cases waste-generating organic solvents are used as reaction media. Moreover, isolation of product iodides presents additional practical issues, such as light sensitivity and hence, limited shelf stability. In terms of commercial availability, iodides tend to be more expensive, should they even be items of commerce.^6^

**Fig. 2:**
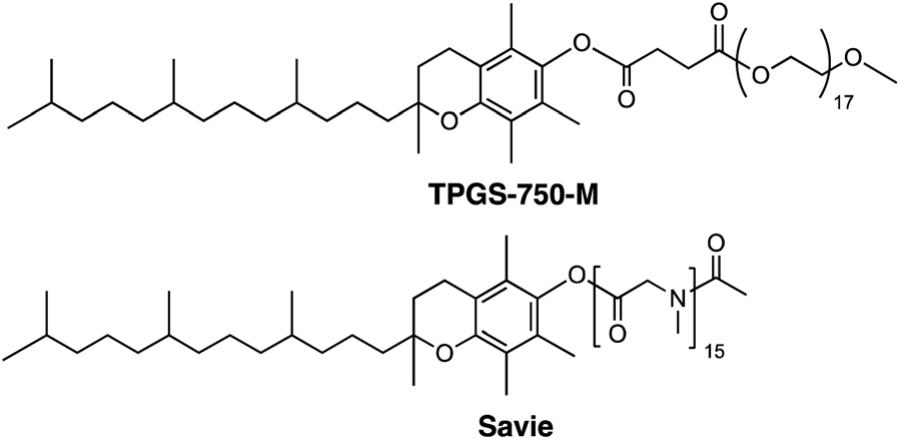
Structures of designer surfactants.

One solution stems from the opportunity to capitalize on the high local concentrations associated with the inner cores of nanomicelles characteristic of aqueous micellar media,^7^ formed by designer surfactants such as TPGS-750-M^8^ and Savie (Fig. 2).^9^ These concentrations are known to be roughly ten times those traditionally utilized in organic solvents,^7^ thereby potentially enabling *in situ* halide interconversion under milder reaction conditions, leading to greater functional group tolerance, in contrast to those that require elevated temperatures.^10^ More importantly, the newly generated iodides could then be used without isolation and/or purification; rather, their direct participation as educts in Pd-catalyzed cross couplings should lead to the targeted products. Such an overall sequence would not only be viewed as sustainable, it would also offer a pathway that does not currently exist to molecules of greater complexity that characterize the fine chemicals industry.

## Results and discussion

**Scheme 1:**
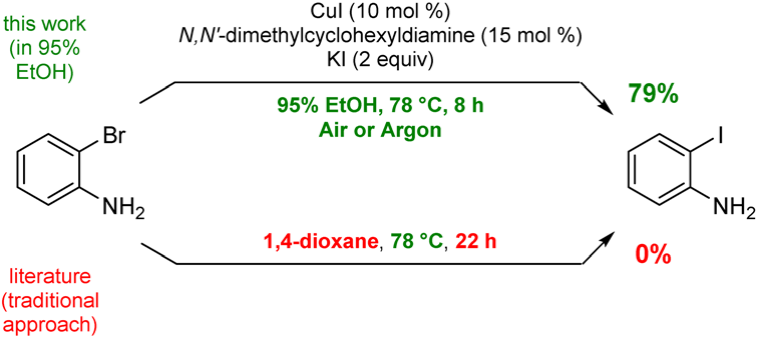
Representative example using an alternative protocol.

**Scheme 2:**
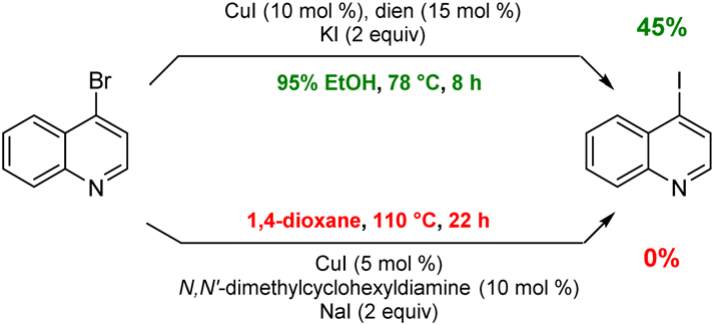
A representative sensitive substrate.

To arrive at an earth-abundant metal-catalyzed first-step conversion of an aryl bromide to the corresponding iodide, various amine-ligated species were screened under micellar catalysis conditions. The combination of a Cu(i) salt (CuI; see Table S5†) and dien ligand (*i.e.*, diethylenetriamine; an industrial waste product formed from the synthesis of ethylenediamine; see Table S1†), afforded the best results, although only a 51% level of conversion was noted. Ultimately, use of green and inexpensive 95% EtOH gave the best levels of conversion (Scheme 1 and see Table S2 in the ESI†). Surprisingly, neither an aqueous micellar medium (containing 2 wt% TPGS-750-M) nor a purely alcoholic medium (100% EtOH) led to competitive levels of conversion. These observations suggest, that the presence of water is required, but that use of an (essentially) exclusively aqueous environment is not ideal. We rationalize this on the basis of solubility, where dien-ligated Cu(i) (as with many Cu(i) complexes) forms a stable species in water,^11^ while in THF the catalyst tends to form insoluble aggregates. Likewise, in CH_3_CN, where it has been used for this purpose, it experiences significant disproportionation especially at the higher temperatures of use and when sensitive substrates are involved (*e.g.*, Scheme 2). As noted in the Jin and Davies work, the Cu-based catalyst system is capable of iodination of certain substrates under an air atmosphere without a loss in conversion (Scheme 1).^2^

**Scheme 3:**
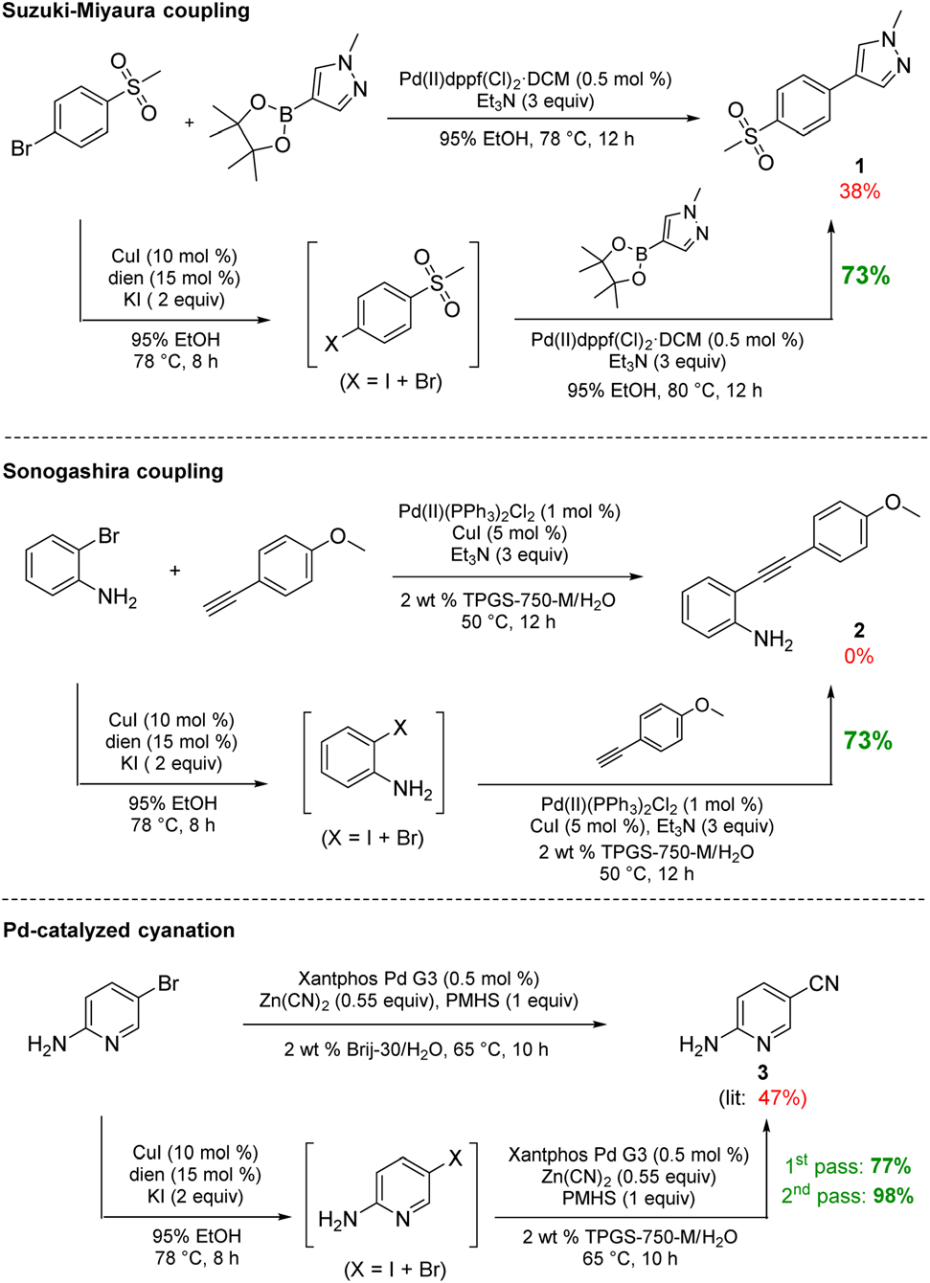
Direct comparisons for Pd-catalyzed couplings using this *in situ* technology.

The initially formed iodides, in each case and without separation or further purification, are amenable to subsequent cross-couplings. Thus, simple filtration to remove copper salts and the amine ligand leads to (crude) materials that are more amenable to Pd-catalyzed couplings than the starting aryl bromides. Unlike all traditional iodination reactions, the high local concentrations associated with the aqueous micellar medium enables highly effective palladium-catalyzed cross-coupling reactions. For example, in the challenging Suzuki–Miyaura coupling illustrated in Scheme 3, a dramatic increase in yield is observed (73%) relative to that obtained from the starting bromide (38%).^12^ Likewise, a Sonogashira coupling proceeded uneventfully to alkyne 2, while the corresponding bromide under identical conditions led to none of the expected product. Lastly, a very challenging Pd-catalyzed cyanation of a 2-aminopyridine documents the potential for additional applications to heteroaromatic systems, as well as being illustrative of the differences in reaction efficiencies to be expected. Direct conversion to pyridylnitrile 3 under related aqueous conditions is reported to afford this product in 47% isolated yield.^13^ Using only a single pass of this iodination/cyanation sequence led to an increase in yield to 77%. However, by simply repeating the iodination on the initially formed crude mixture, followed by subjecting the material (with no purification whatsoever) to the same cyanation gave the targeted product 3 in 98% isolated yield.

**Scheme 4:**
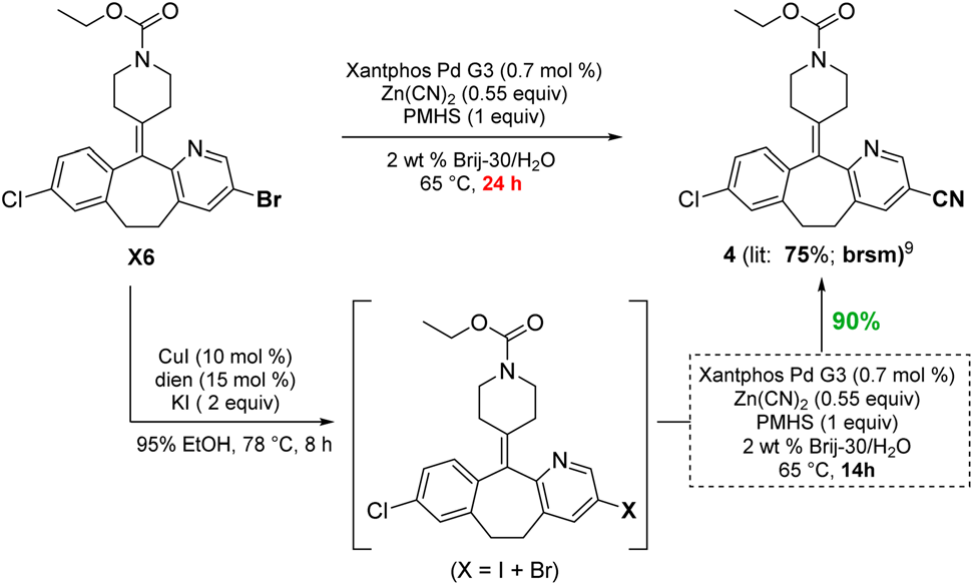
Comparison between an aryl bromide and *in situ*-derived iodide for a Pd-catalyzed cyanation.

To demonstrate functional group tolerance using these mild conditions, an educt from the Merck Informer Library^14^ (X6) was treated under these halide exchange conditions, after which another challenging cyanation could be used to afford product 4, all in 1-pot (Scheme 4). While the bromide is known to afford product 4 in 75% yield based on recovered starting material (brsm),^13^ the iodo-enriched material gave cyanide 4 in 90% isolated yield.

**Scheme 5:**
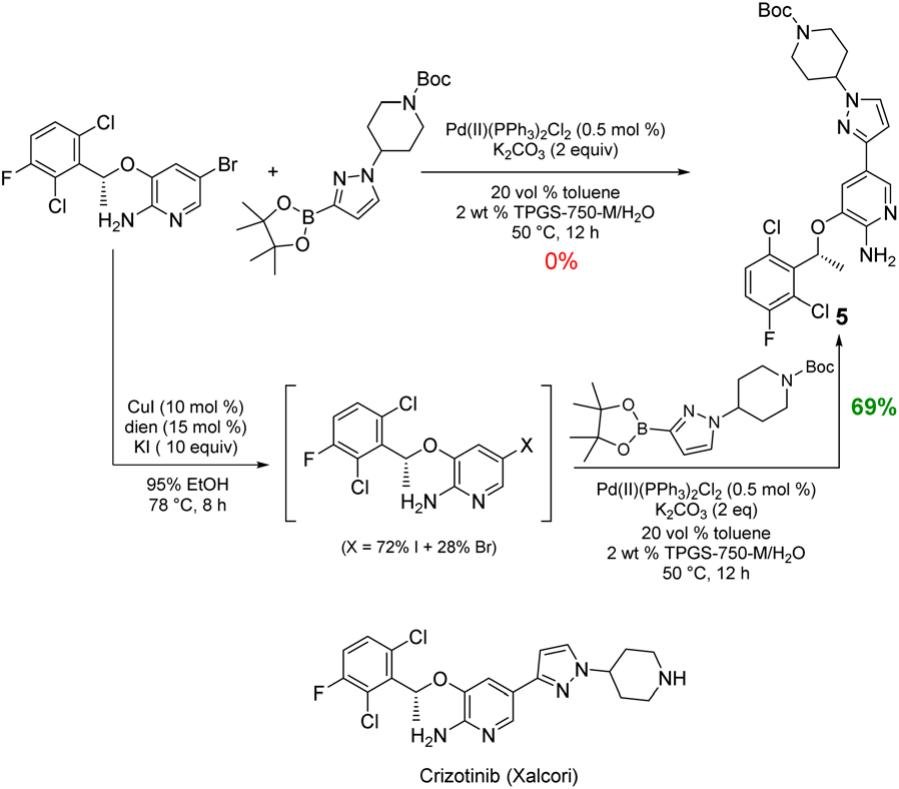
One-pot iodination/Suzuki–Miyaura coupling to 5, the precursor to crizotinib.

Likewise, the penultimate step in Pfizer's discovery route to the $500+ million per year anticancer drug crizotinib requires a Suzuki–Miyaura coupling in the undesirable solvent dimethoxyethane.^15^ The reported coupling also utilizes an unsustainable and costly 4 mol% loading of palladium catalyst (Pd(dppf)Cl_2_), all at 90 °C. The identical coupling run in aqueous TPGS-750-M at 50 °C carried out with the corresponding *in situ*-formed iodide gave the same product 5 in 55% yield (not shown). To further increase the yield, 10 equivalents of KI was used to drive the conversion to the iodide, while subsequent Suzuki–Miyaura coupling to give 5 (69%) required only 0.5 mol% of the far less expensive (triphenylphosphine-based) Pd(PPh_3_)_2_Cl_2_. Under the same aqueous conditions, the corresponding bromide proved unreactive (Scheme 5).

**Scheme 6:**
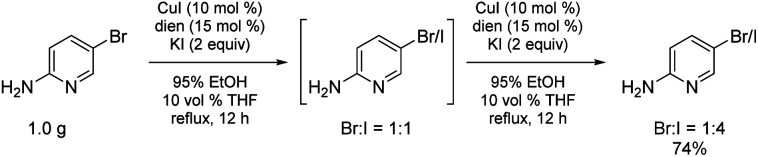
Gram level conversion to the more reactive iodide.

Bromide-to-iodide conversions can be easily scaled to the gram-plus level (Scheme 6), unlike previously reported processes where, *e.g.*, Buchwald, *et al.* recognized the handling and safety issues associated with the high pressures and sealed tubes their conditions necessitated.^16^ Given that these halide inter-conversions are run at 78 °C using inexpensive and environmentally attractive aqueous (95%) ethanol, there is no special equipment involved, translating into an easily adapted and safe process, seemingly independent of scale. Estimated conversions are routinely determined using ^1^H NMR, whether involving one or two exposures of starting bromide to the reaction conditions.

**Scheme 7:**
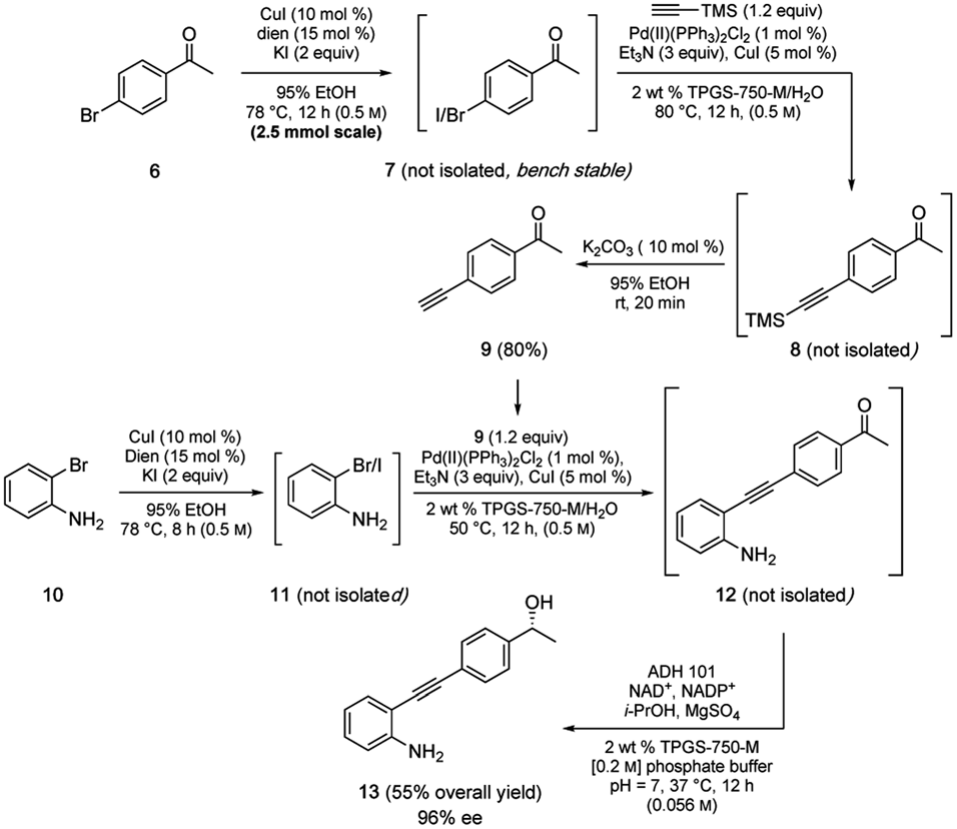
Chemoenzymatic route to disubstituted alkyne 13.

Also worthy of note is that the aqueous conditions associated with this methodology, as has been found to be the case in several other reaction types,^17,18^ appear to be compatible with enzyme-based transformations allowing entry to sequences in the chemoenzymatic regime.^19^ A representative case illustrative of the multitude of possibilities is shown in Scheme 7, which is today typically limited to only two reactions per sequence (*i.e.*, one featuring a chemocatalysis step, the other an enzyme-mediated transformation).^17^ Thus, a 2-pot, multi-step, convergent sequence involving initial iodination of 6 was followed by a Sonogashira coupling of product 7 to alkyne 8, which was desilylated to arrive at terminal alkyne 9 (80%). Coupling partner 11 was prepared *in situ via* iodination of bromide 10, which, without isolation, was converted to diarylalkyne 12. Lastly, ketone 12 was treated with a keto-reductase (KRED) leading to the anticipated nonracemic aminoalcohol 13 (96% ee) in 55% overall isolated yield.

**Scheme 8:**
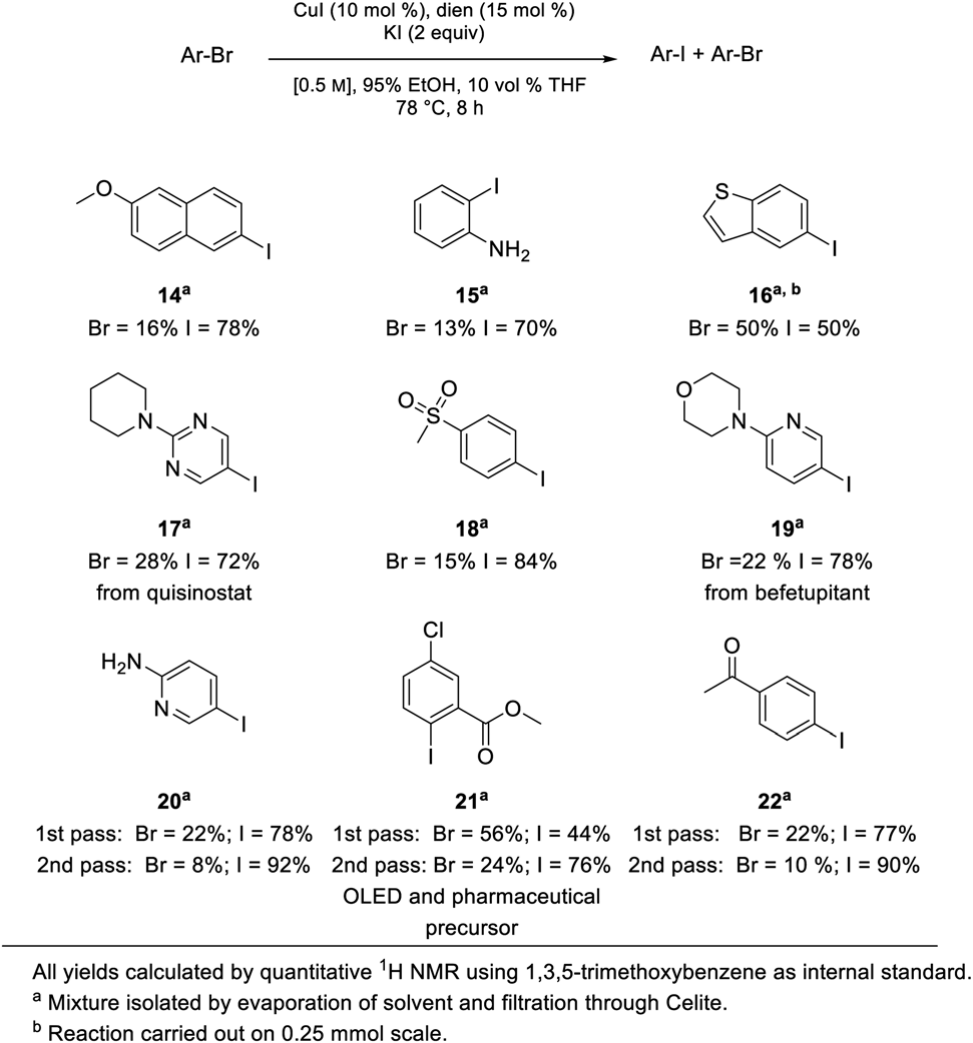
Representative substrate scope.

**Scheme 9:**
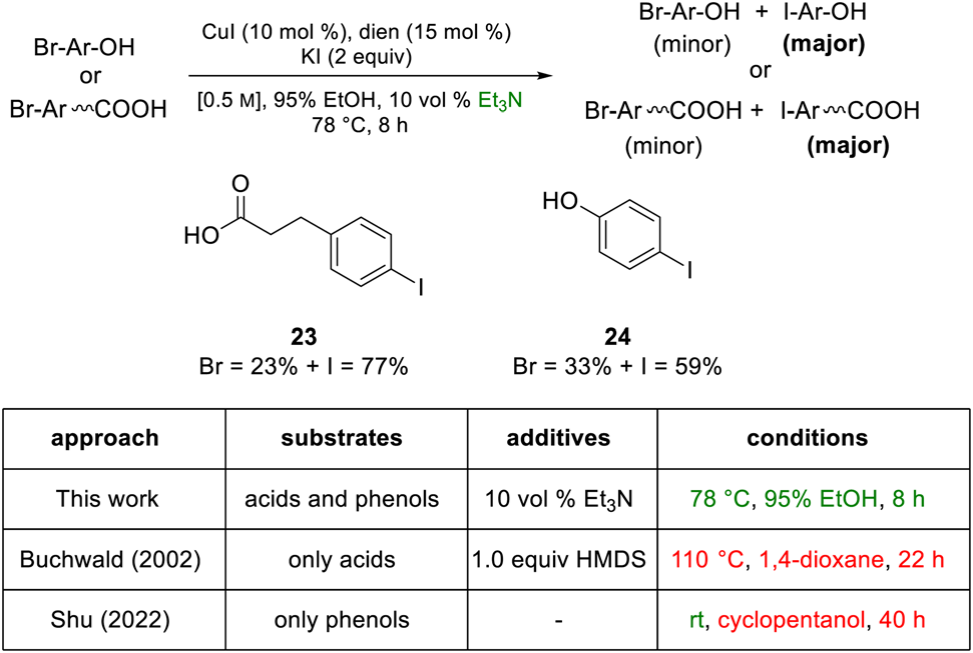
Protic substrates: an acid and a phenol.

Although the most effective ligand identified for this newly introduced capability has been previously described for related bromide-to-iodide only transformations by Jin and Davies,^2^ our system, nonetheless, offers several advantages over prior art. Firstly, the rate at which equilibrium is reached usually requires less than eight hours which, by contrast, is dramatically faster than the 22 and 40 hours characteristic of prior conversions (Scheme 9).^2,3,11,20–22^ Also noteworthy is that the catalyst/solvent system developed accommodates important substrate functionality. Thus, in addition to increased tolerance of sensitive functional groups as documented in the Schemes shown earlier, as well as in Scheme 8, substrates bearing free O–H groups can now readily participate, unlike previous approaches that required considerable manipulation of the hydroxy group.^18,20^

## Conclusions

A safe, scalable, general, and green process has been developed for the *in situ* conversion of unreactive, highly functionalized aromatic and heteroaromatic bromides into useful corresponding iodides that, without isolation and purification, can then be used as cross coupling partners in Pd-catalyzed complex molecule synthesis. These sequences involving two or more reactions take place in 1-pot, and may even include both chemocatalysis as well as biocatalytic events. The potential for applications to targets of value is represented by a sequence leading to the protected form of the antitumor agent crizotinib, which is only viable *via* initial conversion of a heteroaromatic bromide to its reactive iodide. This unprecedented sequential technology is likely to be of particular interest to those in both the discovery and process areas of the pharmaceutical industry.

## Data availability

The synthetic procedures, characterization, and spectral data supporting this article have been uploaded as part of the ESI.†

## Author contributions

R. M. T. conducted most of the experiments and wrote the initial manuscript draft. D. B. O. assisted in conducting experiments and product isolation. B. H. L. oversaw work and aided in drafting the manuscript.

## Conflicts of interest

There are no conflicts to declare.

## Supplementary Material

SC-014-D3SC04199A-s001
