# Supplementary material for: Challenging cross couplings, in water, aided by in situ iodination of (hetero)aromatic bromides
Source: Chem Sci. 2023 Nov 7;14(46):13503–7. doi: 10.1039/d3sc04199a (PMC10686040; doi:10.1039/d3sc04199a)
Supplement: SC-014-D3SC04199A-s001 [file SC-014-D3SC04199A-s001.pdf]

Electronic Supplementary Material (ESI) for Chemical Science.
This journal is © The Royal Society of Chemistry 2023

Electronic Supplementary Information

Challenging cross couplings, in water, aided by *in-situ* iodination of (hetero)aromatic bromides

Rohan M. Thomas, David B. Obbard, and Bruce. H. Lipshutz*

Department of Chemistry and Biochemistry, University of California, Santa Barbara, CA, 93106 USA

Phone : 805-893-2521 Fax : 805-893-8265 Email: lipshutz@chem.ucsb.edu

Contents

1. General Information S2
2. One-Pot Reactions and Procedures S3
3. Large-Scale Reaction Procedures....................S27
4. Optimization Studies....................S27
5. General Procedures S32
6. Characterization data S34
7. References S71

1. General Information

A solution of 2 wt % surfactant/H_2O was prepared by dissolving the surfactant in degassed HPLC grade water and was stored under argon. TPGS-750-M was obtained from a contract manufacturer (PHT International), but is also available from Sigma-Aldrich (catalog #733857). All commercially available reagents were purchased from Sigma-Aldrich, Combi-Blocks, Ambeed Inc., Acros Organics, BLD Pharma, Fischer Scientific, or ChemScene. ADH101 is commercially available from the enzyme kit EZK-001 from Johnson Matthey. NAD+ was purchased from Bioworld and NADP+ from Chem-Impex. Isopropyl alcohol was purchased from VWR. All commercial reagents were used without further purification. Thin layer chromatography (TLC) was done using Silica Gel 60 F254 plates (Merck, 0.25 mm thick). The developed TLC plate was analyzed by a UV lamp (254 nm). The plates were further analyzed with the use of an aqueous ceric ammonium molybdate stain or ethanolic vanillin and developed with a heat gun. All commercially available reagents were used without further purification. Flash chromatography was performed using Silicycle Silicaflash® P60 unbonded grade silica. ^{1}H, ^{13}C, and ^{19}F NMR were recorded at 25 °C on either an Agilent Technologies 400 MHz, a Bruker Avance III HD 400 MHz, or a Agilent Technologies 500 MHz, a Bruker Avance III HD 400 MHz spectrometer in $CDCl_3$ with residual $CHCl_3$ (^{1}H = 7.26 ppm, ^{13}C = 77.16 ppm) as internal standard. Chemical shifts are reported in parts per million (ppm, or Hz). The data presented will be reported as follows: chemical shift, multiplicity (s = singlet, bs = broad singlet, d = doublet, dd = doublet of doublet, t = triplet, q = quartet, quin = quintet, m = multiplet), coupling constant (if applicable), and integration. High-resolution mass analyses (HRMS) were recorded on Waters GCT Premier GC TOF. Gas chromatography-mass spectrometry (GC-MS) analyses were carried out using a Shimadzu GCMS-QP2020NX system with a GC-2030 front end and a J&W HP-5ms column (30 m, 0.25 mm, 0.25 µm film).

2. One-Pot Reactions and Procedures

1-Methyl-4-(4-(methylsulfonyl)phenyl)-1H-pyrazole (1)

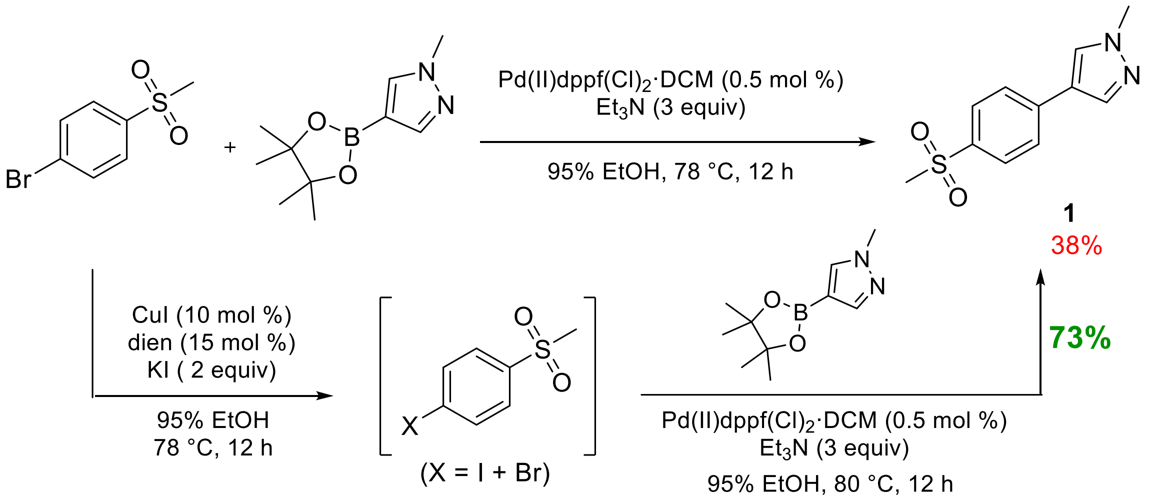

From aryl bromide:

To an oven dried 1 dram vial, 4-bromophenyl methyl sulfone (117.5 mg, 0.5 mmol, 1 equiv), 1-methyl-1H-pyrazol-4-boronic acid pinacol ester (124.8 mg, 0.6 mmol, 1.2 equiv) were added along with a ptfe-coated magnetic stir bar. The reaction vial was brought into an argon filled glovebox where [1,1′-*bis*(diphenylphosphino)ferrocene]dichloropalladium(II) DCM adduct (2.0 mg, 0.0025 mmol, 5000 ppm) was added and the reaction vial was capped with a rubber septum. The capped vial was removed from the glovebox and through the septum, de-gassed triethylamine (151.8 mg, 209 µL, 3 equiv) and 0.9 mL of de-gassed 95% ethanol were added. The vial was then placed in a pre-heated aluminum heating block over a stir plate with a thermocouple probe in the aluminum block set to 83 °C to give an internal vial temperature of 78 °C for 12 h.

The reaction was then removed from the heat and allowed to cool to rt. Ethanol was removed under vacuum and 1 mL of DI H_2O was added and the aqueous solution subsequently extracted with 3 x 1 mL portions of EtOAc. The combined organic phase was subsequently dried onto silica and the product purified by flash chromatography (70% EtOAc/30% hexanes) to yield **1** as an off-white solid (45.0 mg, 38% yield).

From aryl iodide:

To an oven dried 1 dram vial, 4-bromophenyl methyl sulfone (117.5 mg, 0.5 mmol, 1 equiv), KI (166.0 mg, 1.0 mmol, 2.0 equiv) were added along with a ptfe-coated magnetic stirbar. The vial was then transferred to an argon-filled glove box where CuI (9.6 mg, 0.05 mmol, 0.1 equiv) was added. The vial was then capped with a rubber septum and removed from the glove box. Through the septum, 100 µL of de-gassed THF as co-solvent was added followed by diethylenetriamine (8 µL, 0.075 mmol, 0.15 equiv) as a stock solution in 0.9 mL of 95% ethanol that was rigorously

degassed with Ar. The reaction was sealed with PTFE-tape and placed into a preheated aluminum block reactor set to 83 °C (internal temperature 78 °C) and left to react for 8 h.

Upon completion, the vial was removed from the heating block and allowed to cool to rt. The ethanol was removed under vacuum and the residue suspended in 1 mL of EtOAc. This residue was then filtered through a short plug of silica and the silica washed with 3 x 1 mL portions of EtOAc back into the same vial. The solvent was removed under vacuum and 1-methyl-1H-pyrazol-4-boronic acid pinacol ester (124.8 mg, 0.6 mmol, 1.2 equiv) was added as a solid. The reaction vial was then brought into an argon filled glovebox where [1,1′-Bis(diphenylphosphino)-ferrocene]dichloropalladium(II) DCM adduct (2.0 mg, 0.0025 mmol, 5000 ppm) was added and the reaction vial was capped with a rubber septum. The capped vial was removed from the glovebox and through the septum, de-gassed triethylamine (151.8 mg, 209 µL, 3 equiv) and 0.9 mL de-gassed 95% ethanol were added. The vial was then placed in a pre-heated aluminum heating block over a stir plate with thermocouple probe in the aluminum block set to 83 °C to give an internal vial temperature of 78 °C for 12 h.

The reaction was then removed from the heat and allowed to cool to rt. Ethanol was removed under vacuum and 1 mL of DI H_2O was added and the aqueous solution subsequently extracted with 3 x 1 mL portions of EtOAc. The combined organic phase was subsequently dried onto silica and the product purified by flash chromatography (70% EtOAc/30% hexanes, R_f: 0.30) to yield **1** as an off-white solid (86.1 mg, 73% yield).

^{1}H NMR (500 MHz, $CDCl_3$) δ 7.94 – 7.88 (m, 2H), 7.83 (s, 1H), 7.72 (s, 1H), 7.66 – 7.61 (m, 2H), 3.97 (s, 3H), 3.06 (s, 3H).. ^{13}C NMR (126 MHz, $CDCl_3$) 138.4, 137.8, 137.2, 128.2, 127.9, 125.8, 121.4, 44.7, 39.3.

Spectral data matched those previously obtained.[23]

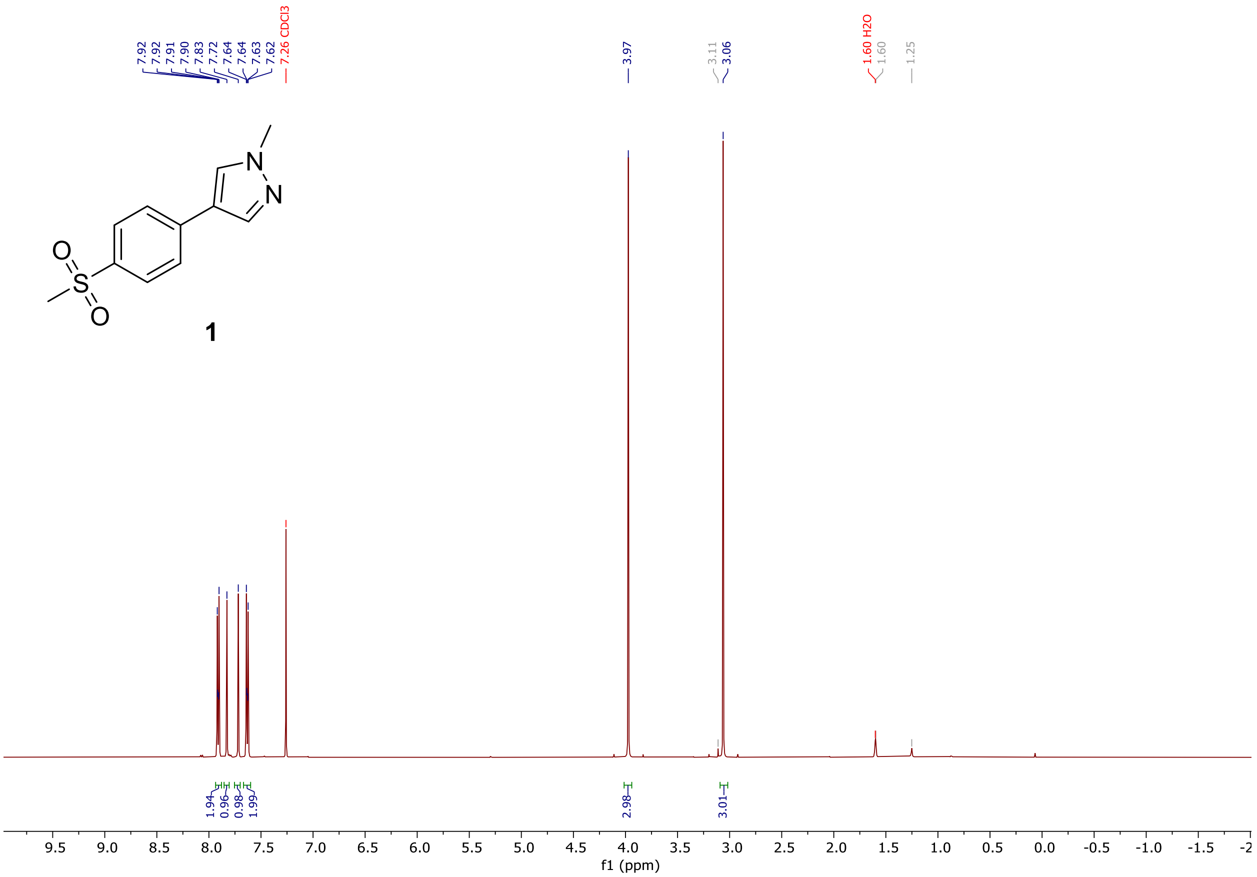

Figure S1. ^{1}H NMR spectrum of **1** in $CDCl_3$

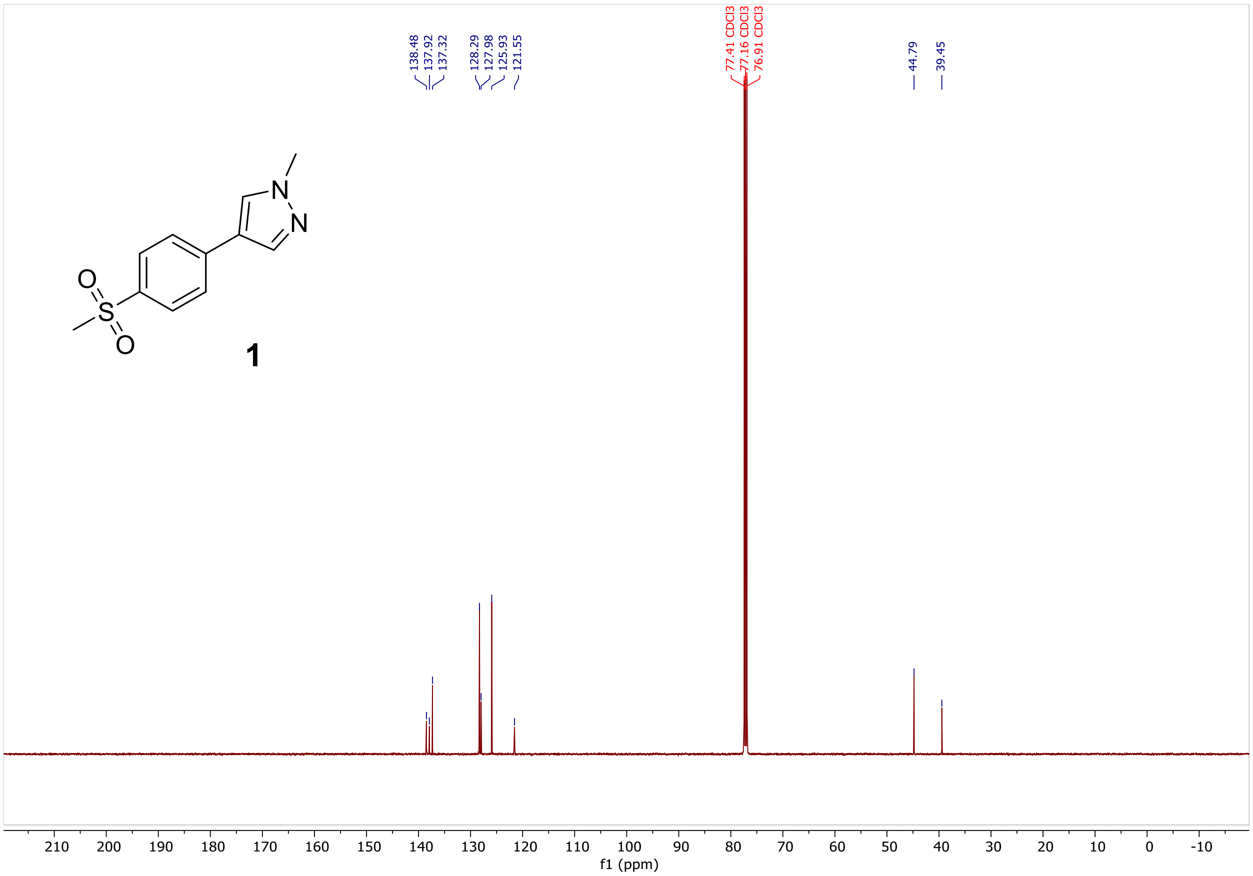

Figure S2. ^{13}C NMR spectrum of **1** in $CDCl_3$

2-((4-Methoxyphenyl)ethynyl)aniline (2)

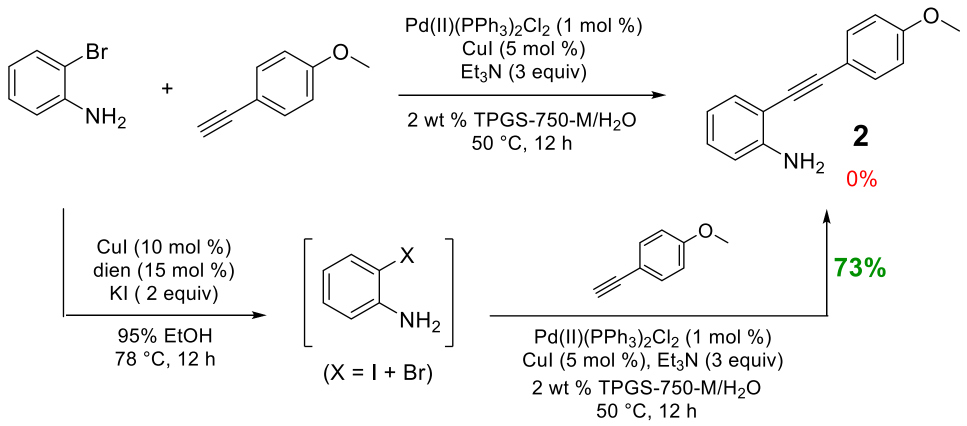

To an oven dried 1 dram vial, 2-bromoaniline (43.0 mg, 0.25 mmol, 1 equiv), KI (83.0 mg, 0.5 mmol, 2.0 equiv) were added along with a ptfe-coated magnetic stirbar. The vial was then transferred to an argon-filled glove box where CuI (4.8 mg, 0.025 mmol, 0.1 equiv) was added. The vial was then capped with a rubber septum and removed from the glove box. Through the septum, 50 µL of de-gassed THF as co-solvent was added followed by diethylenetriamine (4 µL, 0.0375 mmol, 0.15 equiv) as a stock solution in 0.45 mL of 95% ethanol that was rigorously degassed with Ar. The reaction was sealed with PTFE-tape and placed into a preheated aluminum block reactor set to 83 °C (internal temperature 78 °C) and left to react for 8 h.

Upon completion, the vial was removed from the heating block and allowed to cool to rt. The ethanol was removed under vacuum and *bis*-triphenylphosphine palladium(II)Cl_2 (1.8 mg, 0.0025 mmol, 0.01 equiv) was added to the crude residue and the vial brought into an argon-filled glove box where CuI (2.4 mg, 0.0125 mmol, 0.05 equiv) was added and the vial capped with a rubber septum. The vial was removed from the glove box and through the septum, de-gassed triethylamine (75.9 mg, 105 µL, 3 equiv), 0.4 mL de-gassed 2 wt % TPGS-750-M/H_2O, and 4-ethynylanisole (40.0 mg, 0.3 mmol, 39 µL, 1.2 equiv) were added sequentially. The vial was then placed in a pre-heated aluminum heating block over a stir plate with thermocouple probe in the aluminum block set to 55 °C to give an internal vial temperature of 50 °C for 12 h.

The reaction was then removed from the heat and allowed to cool to rt. The aqueous reaction mixture was extracted with 3 x 1 mL portions of EtOAc. The combined organic phase was subsequently dried onto silica and the product purified by flash chromatography (15% EtOAc/ 85% hexanes, R_f: 0.40) to yield **2** as a yellow solid (40.9 mg, 73% yield).

^{1}H NMR (500 MHz, $CDCl_3$) δ 7.51 – 7.42 (m, 2H), 7.35 (dd, *J* = 7.6, 1.5 Hz, 1H), 7.12 (ddd, *J* = 8.5, 7.4, 1.5 Hz, 1H), 6.92 – 6.84 (m, 2H), 6.75 – 6.67 (m, 2H), 4.25 (s, 2H), 3.82 (s, 3H).^{13}C NMR (126 MHz, $CDCl_3$) δ ^{13}C NMR (126 MHz, $CDCl_3$) δ 159.5, 147.5, 132.8, 131.9, 129.3, 117.9, 115.3, 114.1, 113.9, 108.3, 94.5, 84.3, 55.2.

Spectral data matched those previously obtained.[24]

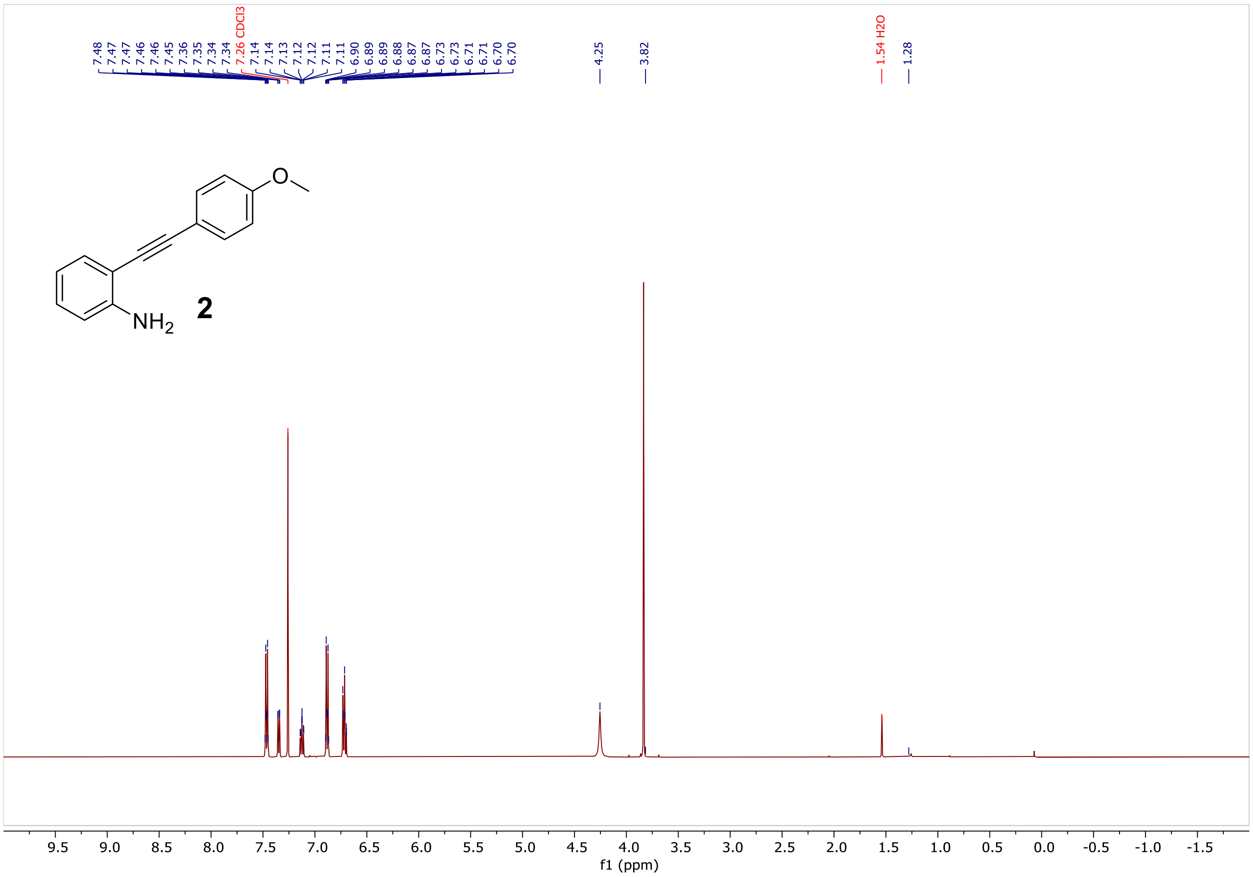

Figure S3. ^{1}H NMR spectrum of **2** in $CDCl_3$

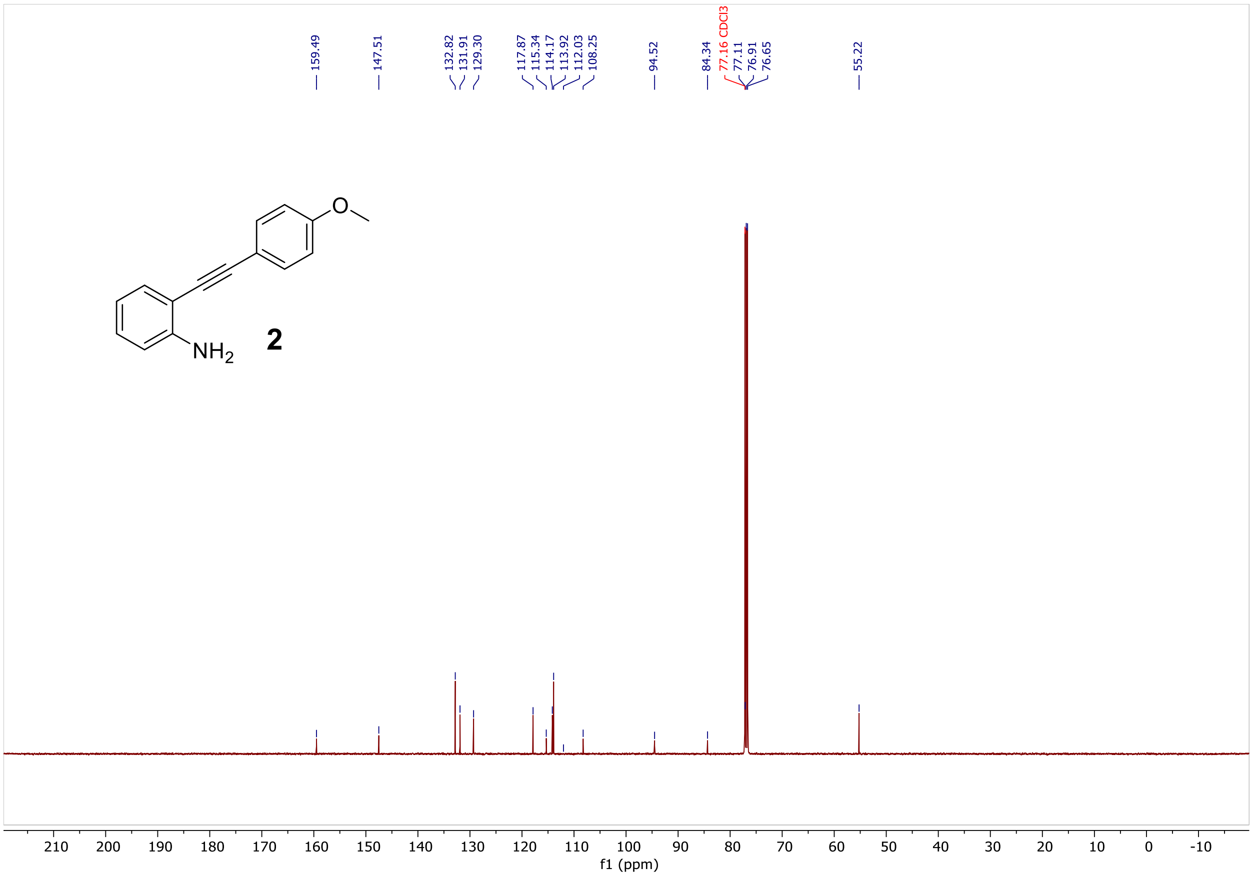

Figure S4. ^{13}C NMR spectrum of **2** in $CDCl_3$

5-Cyanopyridin-2-amine (3)

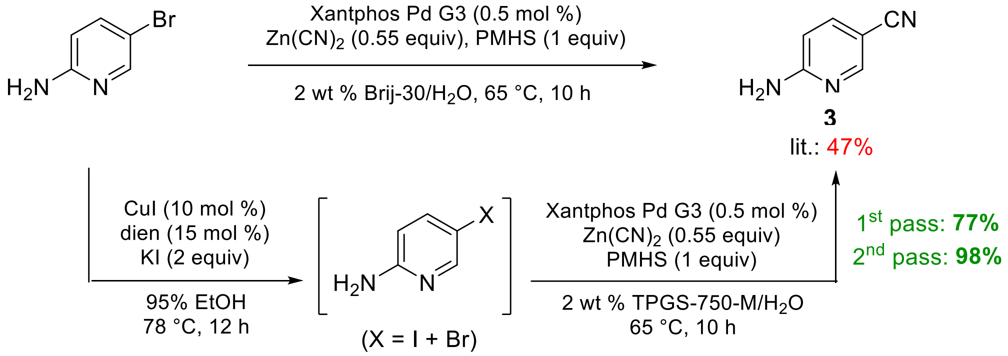

To an oven dried 1 dram vial, 5-bromopyridin-2-amine (87.0 mg, 0.5 mmol), KI (83.0 mg, 0.5 mmol, 2 equiv) were added along with a ptfe-coated magnetic stir bar. The vial was then transferred to an argon-filled glove box where CuI (4.8 mg, 0.025 mmol, 0.1 equiv) was added. The vial was then capped with a rubber septum and removed from the glove box. Through the septum, 50 µL of de-gassed THF as co-solvent was added followed by diethylenetriamine (4 µL, 0.0375 mmol, 0.15 equiv) as a stock solution in 0.45 mL of 95% ethanol that was rigorously degassed with Ar. The reaction was sealed with PTFE-tape and placed into a preheated aluminum block reactor set to 83 °C (internal temperature 78 °C) and left to react for 8 h. For the reaction utilizing a second-pass iodination, see General Procedure 3.

Upon completion, the vial was removed from the heating block and allowed to cool to rt. The ethanol was removed under vacuum and Xantphos Pd G3 (2.4 mg, 0.0025 mmol, 0.005 equiv as 100 µL of a stock solution in degassed DCM) was added to the crude residue after which a high-vacuum was applied to remove the DCM. To this vial, $ZnCN_2$ (32.0 mg, 0.275 mmol, 0.55 equiv) was added and the vial brought into an argon-filled glove box and capped with a rubber septum. The vial was removed from the glove box and through the septum, de-gassed PMHS from Sigma-Aldrich (32 µL, 1.0 equiv), 100 µL degassed THF, and 0.9 mL of de-gassed 2 wt % TPGS-750-M/H_2O were added sequentially. The vial was then placed in a pre-heated aluminum heating block over a stir plate with thermocouple probe in the aluminum block set to 70 °C to give an internal vial temperature of 65 °C for 10 h.

The reaction was then removed from the heat and allowed to cool to rt. The aqueous reaction mixture was extracted with 3 x 1 mL portions of EtOAc. The combined organic phase was subsequently dried onto silica and the product purified by flash chromatography (50% EtOAc/ 50% hexanes, R_f: 0.50) to yield **3** as a pale yellow solid (1st pass: 45.3 mg, 77% yield; 2nd pass: 66.4 mg, 98% yield).

^{1}H NMR (400 MHz, $CDCl_3$) δ 8.36 (d, J = 2.2 Hz, 1H), 7.61 (dd, J = 8.7, 2.2 Hz, 1H), 6.50 (d, J = 8.6 Hz, 1H), 4.97 (s, 2H). ^{13}C NMR (126 MHz, $CDCl_3$) δ 160.1, 153.2, 140.2, 118.1, 108.0, 98.5.

Spectral data matched those previously obtained.[25]

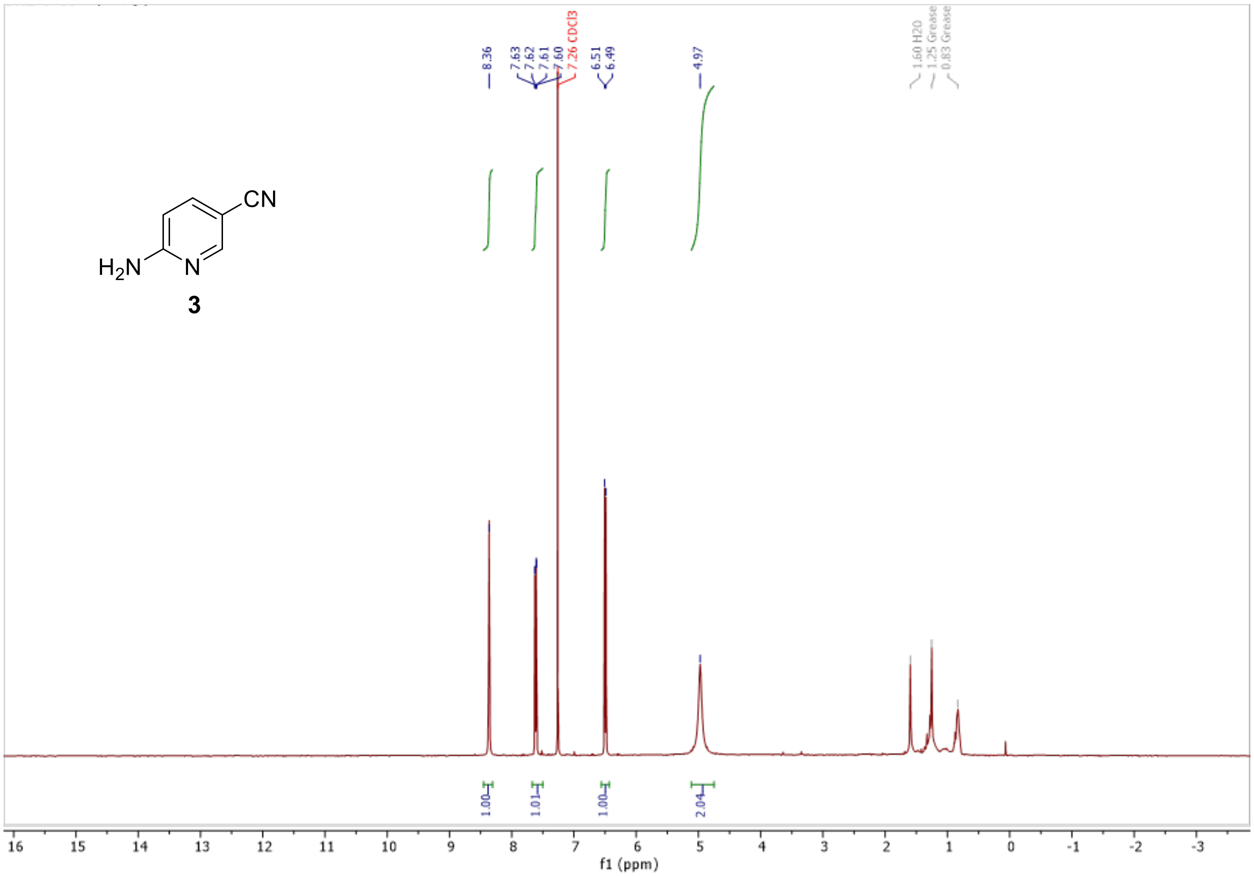

Figure S5. ^{1}H NMR spectrum of **3** in $CDCl_3$

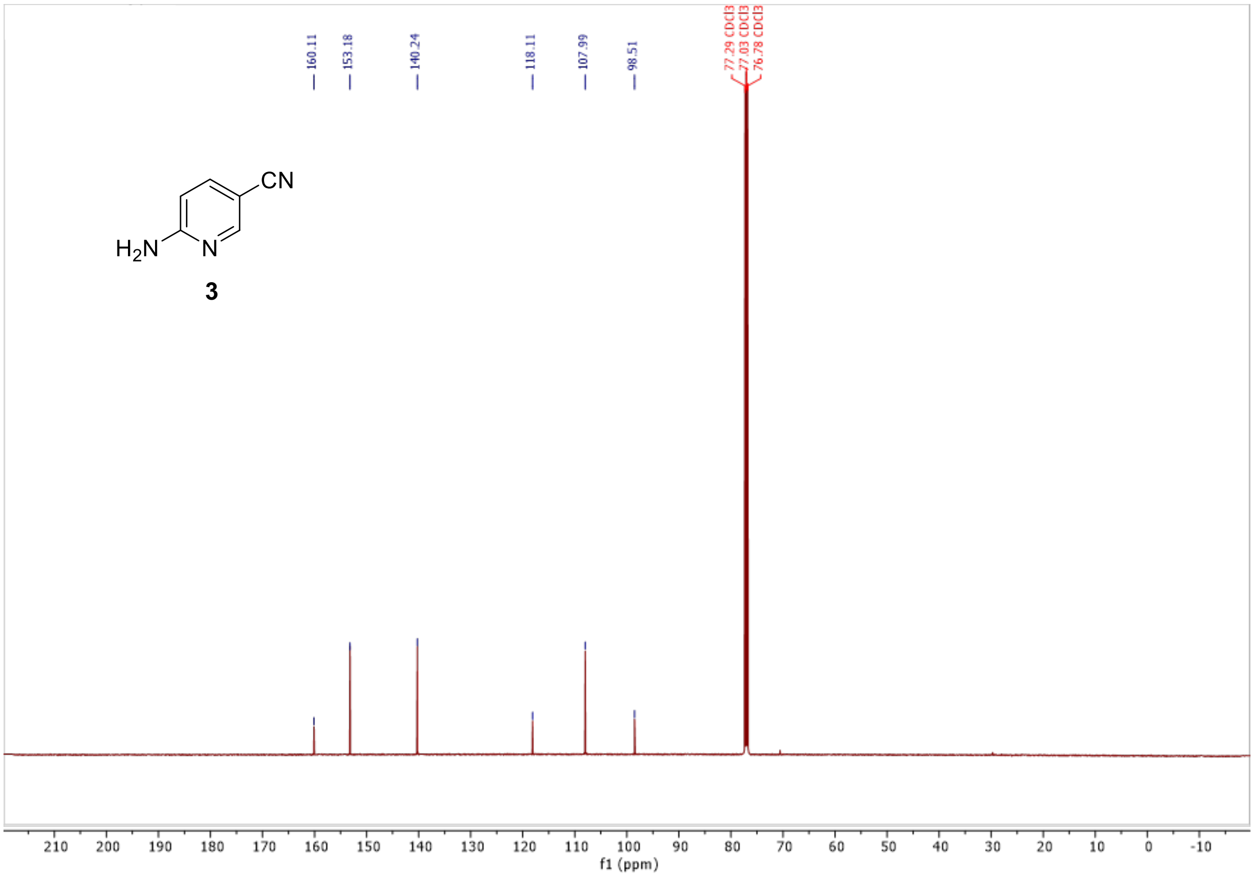

Figure S6. ^{13}C NMR spectrum of **3** in $CDCl_3$

Ethyl 4-(3-cyano-8-chloro-5,6-dihydro-11*H*-benzo[5,6]cyclohepta[1,2-*b*]pyridin-11-ylidene)piperidine-1-carboxylate (4)

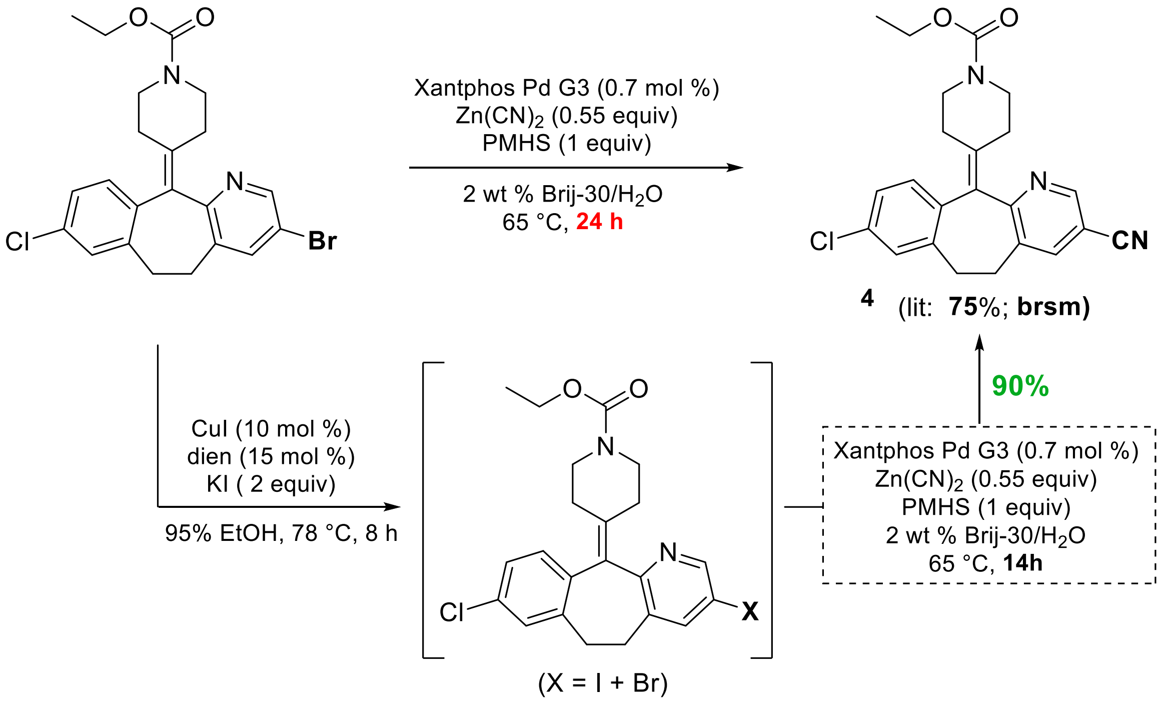

To an oven dried 1 dram vial, ethyl 4-(3-bromo-8-chloro-5,6-dihydro-11*H*-benzo[5,6]cyclohepta-[1,2-*b*]pyridin-11-ylidene)piperidine-1-carboxylate (46.0 mg, 0.1 mmol), KI (17.0 mg, 0.2 mmol, 2.0 equiv) were added along with a ptfe-coated magnetic stir bar. The vial was then transferred to an argon-filled glove box where CuI (1.0 mg, 0.001 mmol, 0.1 equiv) was added. The vial was then capped with a rubber septum and removed from the glove box. Through the septum, 50 µL of de-gassed THF as co-solvent was added followed by diethylenetriamine (0.8 µL, 0.015 mmol, 0.15 equiv) as a stock solution in 0.45 mL 95% ethanol that was rigorously degassed with Ar. The reaction was sealed with PTFE-tape and placed into a preheated aluminum block reactor set to 83 °C (internal temperature 78 °C) and left to react for 8 h.

Upon completion, the vial was removed from the heating block and allowed to cool to rt. The ethanol was removed under vacuum and Xantphos Pd G3 (0.7 mg, 0.0007 mmol, 0.007 equiv as 28 µL of a stock solution in degassed DCM) was added to the crude residue after which high-vacuum was applied to remove the DCM. To this vial, $ZnCN_2$ (6.4 mg, 0.055 mmol, 0.55 equiv) was added and the vial brought into an argon-filled glove box and capped with a rubber septum. The vial was removed from the glove box and through the septum, de-gassed PMHS (6.4 µL, 1.0 eq), 25 µL degassed THF, and 0.225 mL de-gassed 2 wt % Brij-30/H_2O were added sequentially. The vial was then placed in a pre-heated aluminum heating block over a stir plate with thermocouple probe in the aluminum block set to 70 °C to give an internal vial temperature of 65 °C for 14 h.

The reaction was then removed from the heat and allowed to cool to rt. The aqueous reaction mixture was extracted with 3 x 1 mL portions of EtOAc. The combined organic phase was subsequently dried onto silica and the product purified by flash chromatography (40% EtOAc/ 60% hexanes) to yield **4** as a pale yellow solid (41.0 mg mg, 90.5% yield).

^{1}H NMR (500 MHz, $CDCl_3$) δ 8.70 (d, *J* = 2.1 Hz, 1H), 7.74 (d, *J* = 2.0 Hz, 1H), 7.24 – 7.17 (m, 2H), 7.12 (d, *J* = 8.1 Hz, 1H), 4.17 (q, *J* = 7.1 Hz, 2H), 3.77 (br s, 2H), 3.44 – 3.36 (m, 2H), 3.21 (ddt, *J* = 13.1, 8.8, 4.1 Hz, 2H), 2.90 (ddd, *J* = 16.0, 10.1, 4.7 Hz, 1H), 2.82 (ddd, *J* = 16.3, 8.1, 4.6 Hz, 1H), 2.48 (ddd, *J* = 13.9, 9.0, 4.5 Hz, 1H), 2.40 –2.34 (m, 1H), 2.33-2.22 (m, 2H), f1.28 (t, *J* = 7.1 Hz, 3H). ^{13}C NMR (126 MHz, $CDCl_3$) δ 160.8, 155.4, 149.3, 140.5, 139.9, 139.0, 136.6, 134.1, 133.7, 133.0, 130.7, 129.1, 126.7, 116.6, 108.3, 61.5, 44.8, 44.7, 31.4, 31.1, 30.9, 30.7, 14.7.

Spectral data matched those previously obtained.[25]

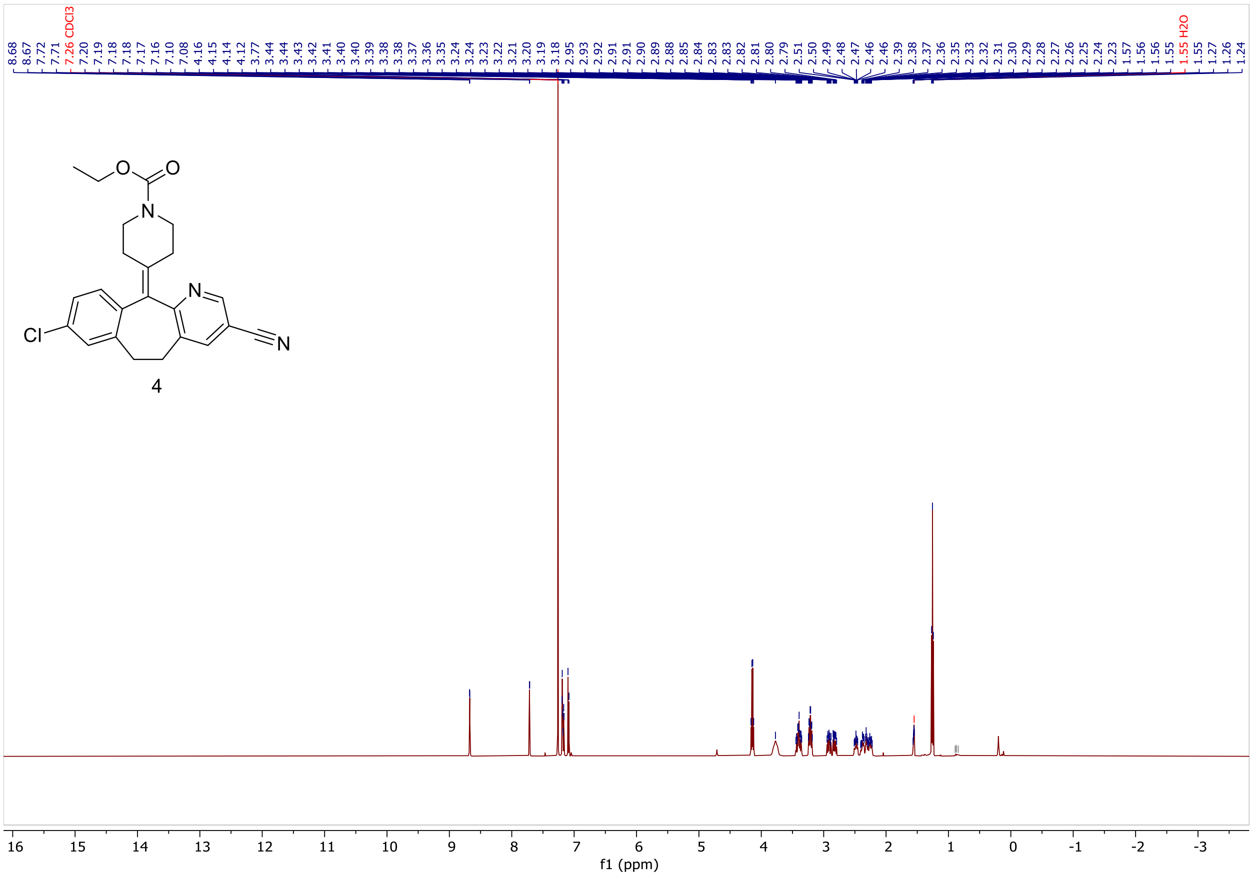

Figure S7. ^{1}H NMR spectrum of **4** in $CDCl_3$

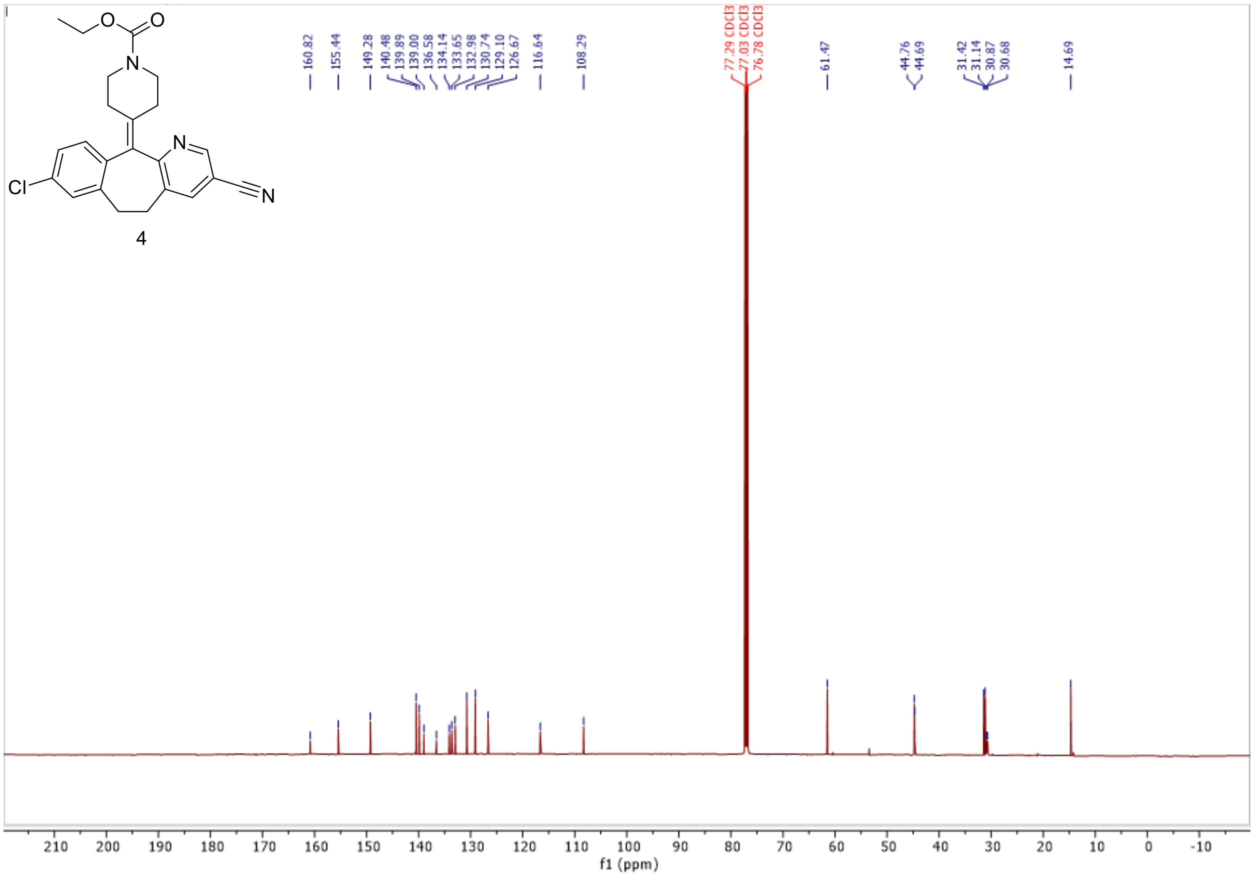

Figure S8. ^{13}C NMR spectrum of **4** in $CDCl_3$

4-(4-{6-Amino-5-[(R)-1-(2,6-dichloro-3-fluoro-phenyl)-ethoxy]-pyridin-3-yl}-pyrazol-1-yl)-piperidine-1-carboxylic acid *t*-butyl ester (5)

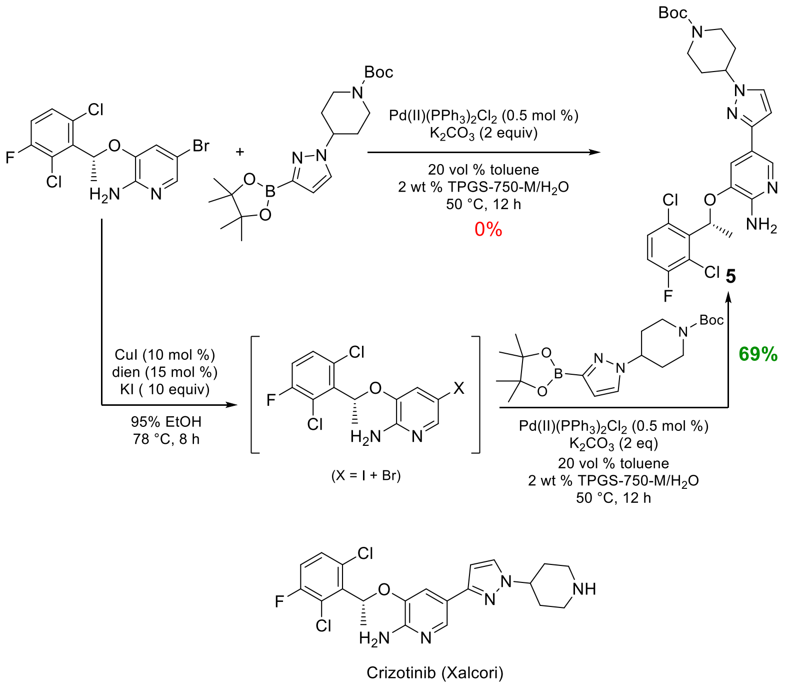

To an oven dried 1 dram vial, 3-[(*R*)-1-(2,6-dichloro-3-fluorophenyl)-ethoxy]-5-bromo-pyridin-2-ylamine (190.0 mg, 0.5 mmol, 1 equiv), KI (830.0 mg, 5.0 mmol, 10.0 eq) were added along with a ptfe-coated magnetic stir bar. The vial was then transferred to an argon-filled glove box where CuI (9.6 mg, 0.05 mmol, 0.1 equiv) was added. The vial was then capped with a rubber septum and removed from the glove box. Through the septum, 100 µL of de-gassed THF as co-solvent was added followed by diethylenetriamine (8 µL, 0.075 mmol, 0.15 equiv) as a stock solution in 0.9 mL 95% ethanol that was rigorously degassed with Ar. The reaction was sealed with PTFE-tape and placed into a preheated aluminum block reactor set to 83 °C (internal temperature 78 °C) and left to react for 8 h.

Upon completion, the vial was removed from the heating block and allowed to cool to rt. The ethanol was removed under vacuum and the residue suspended in 1 mL EtOAc. This residue was then filtered through a short plug of silica and the silica washed with 3 x 1 mL portions of EtOAc back into the same vial. The solvent was removed under vacuum and *t*-butyl 4-(4-(4,4,5,5-tetramethyl-1,3,2-dioxaborolan-2-yl)-1H-pyrazol-1-yl)piperidine-1-carboxylate (207.0 mg, 0.55 mmol, 1.1 equiv), *bis*-triphenylphosphine palladium(II)Cl_2 (0.9 mg, 0.0025 mmol, 0.005 equiv), and anhydrous K_2CO_3 (138.0 mg, 1.0 mmol, 2 equiv) were added. The reaction vial was brought into an argon filled glovebox and capped with a rubber septum. The capped vial was removed from the glovebox and through the septum, de-gassed toluene (200 µL) and 0.8 mL de-gassed 2

wt % TPGS-750-M/H_2O were added. The vial was then placed in a pre-heated aluminum heating block over a stir plate with thermocouple probe in the aluminum block set to 55 °C to give an internal vial temperature of 50 °C for 12 h.

The reaction was then removed from the heat and allowed to cool to rt. The reaction was extracted with 3 x 1 mL portions of EtOAc. The combined organic phase was subsequently dried onto silica and the product purified by flash chromatography (80% EtOAc/20% hexanes, R_f: 0.30) to yield **5** as a dark foam. This foam was dissolved in 0.5 mL of toluene at 60 °C to which 1 mL of *n*-heptane at 60 °C was added. The suspension was allowed to cool to rt and left to stir for 10 h. After this, the mixture was filtered, and the solid washed with hexane and dried under high-vacuum at 60 °C for 12h. (189.7 mg, 69% yield).

^{1}H NMR (500 MHz, $CDCl_3$) δ 7.75 (s, 1H), 7.56 (s, 1H), 7.48 (s, 1H), 7.30 (dd, *J* = 8.9, 4.7 Hz, 1H), 7.05 (t, *J* = 8.3 Hz, 1H), 6.88 – 6.84 (m, 1H), 6.07 (q, *J* = 6.7 Hz, 1H), 4.76 (s, 2H), 4.29 – 4.19 (m, 3H), 2.89 (t, *J* = 12.4 Hz, 2H), 2.16 – 2.09 (m, 2H), 1.98 – 1.83 (m, 5H), 1.48 (s, 9H). ^{13}C NMR (126 MHz, $CDCl_3$) δ 157.5 (d, *J* = 250.74 Hz), 154.6, 149.0, 139.8, 137.0, 135.9, 135.6, 129.9, 129.0 (d, *J* = 3.78 Hz), 122.6, 122.1 (d, *J* = 250.74 Hz), 120.1, 119.1, 116.7 (d, *J* = 23.94 Hz), 114.9, 79.9, 72.4, 59.4, 32.4, 28.4, 18.9.
^{19}F NMR (471 MHz, $CDCl_3$) δ -112.04.

Spectral data matched those previously obtained.[26]

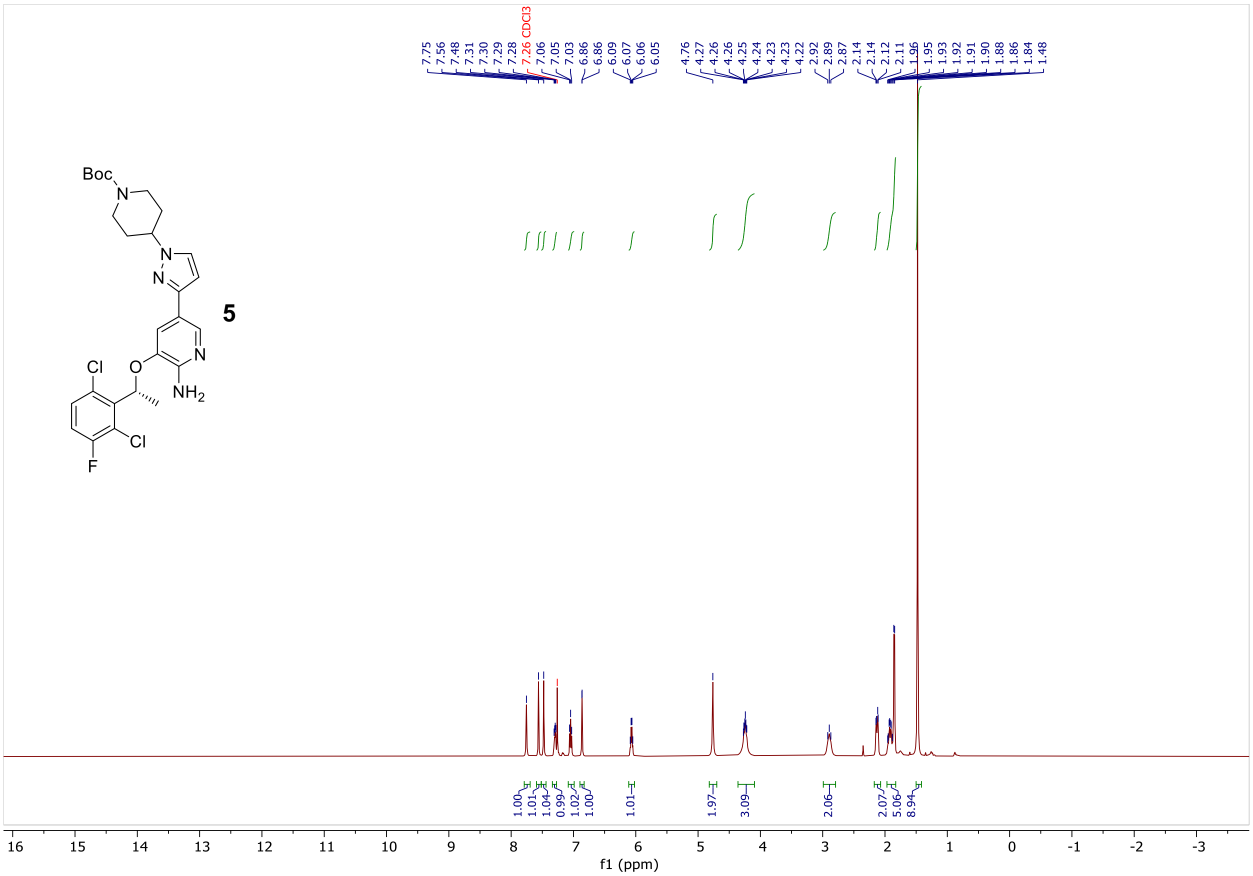

Figure S9. ^{1}H NMR spectrum of **5** in $CDCl_3$

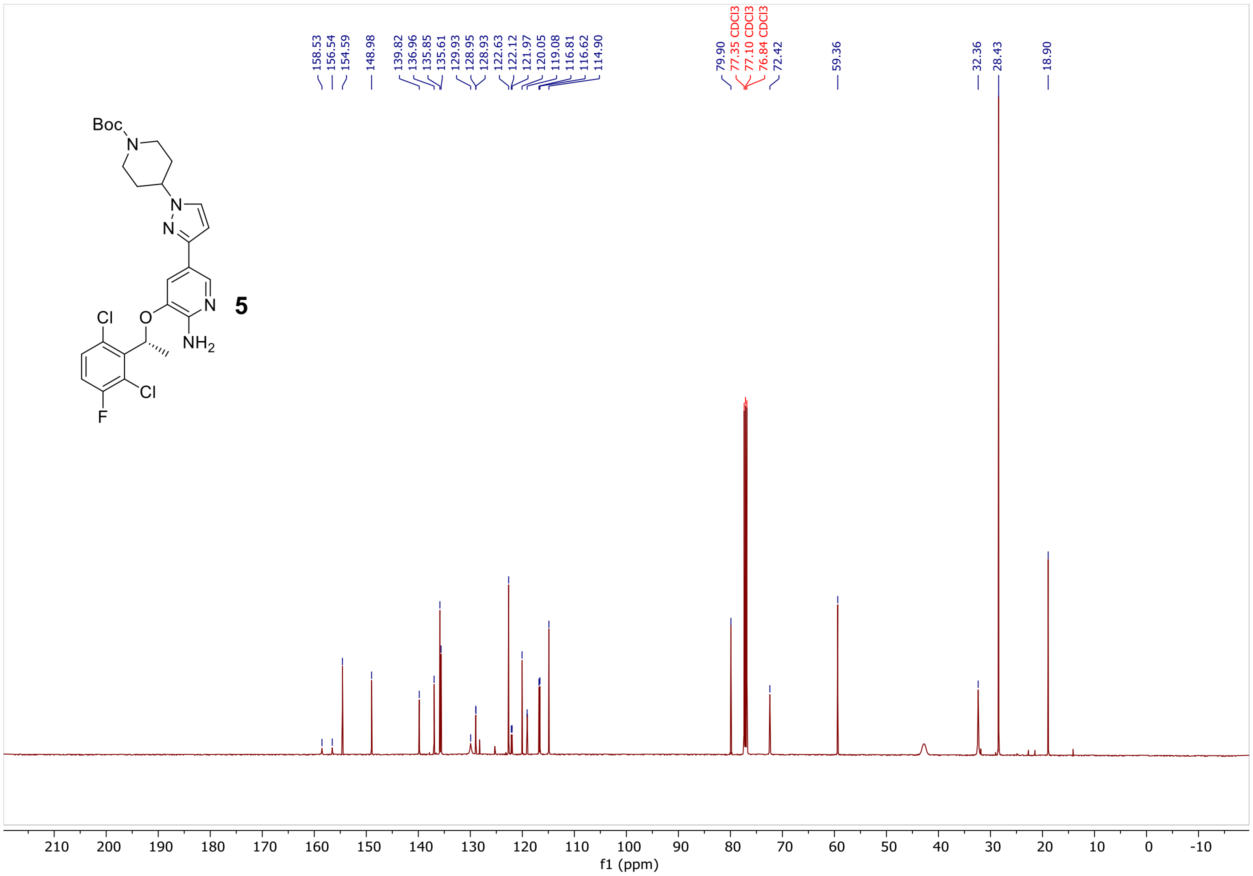

Figure S10. ^{13}C NMR spectrum of **5** in $CDCl_3$

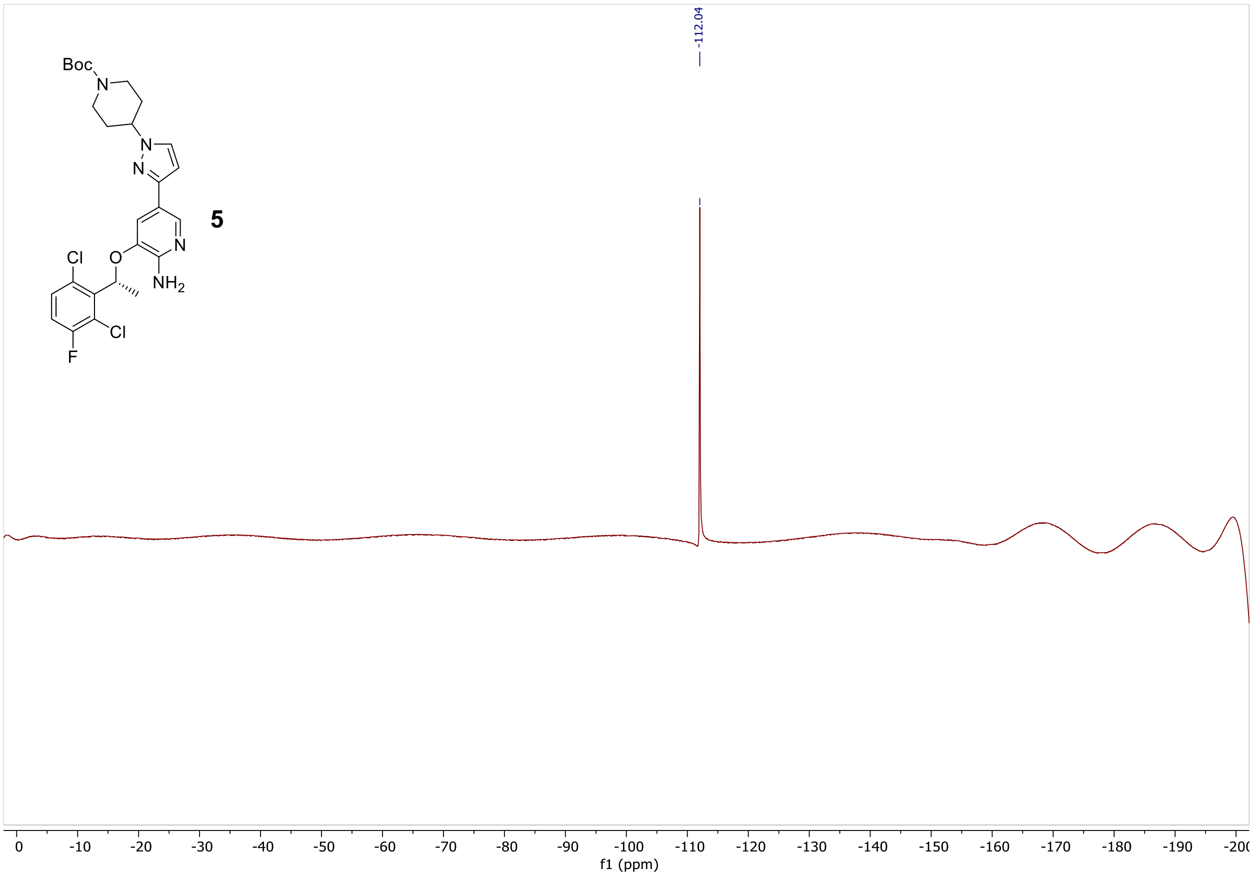

Figure S11. ^{19}F NMR spectrum of **5** in $CDCl_3$

Multi-step, 1-pot convergent synthesis of **13**

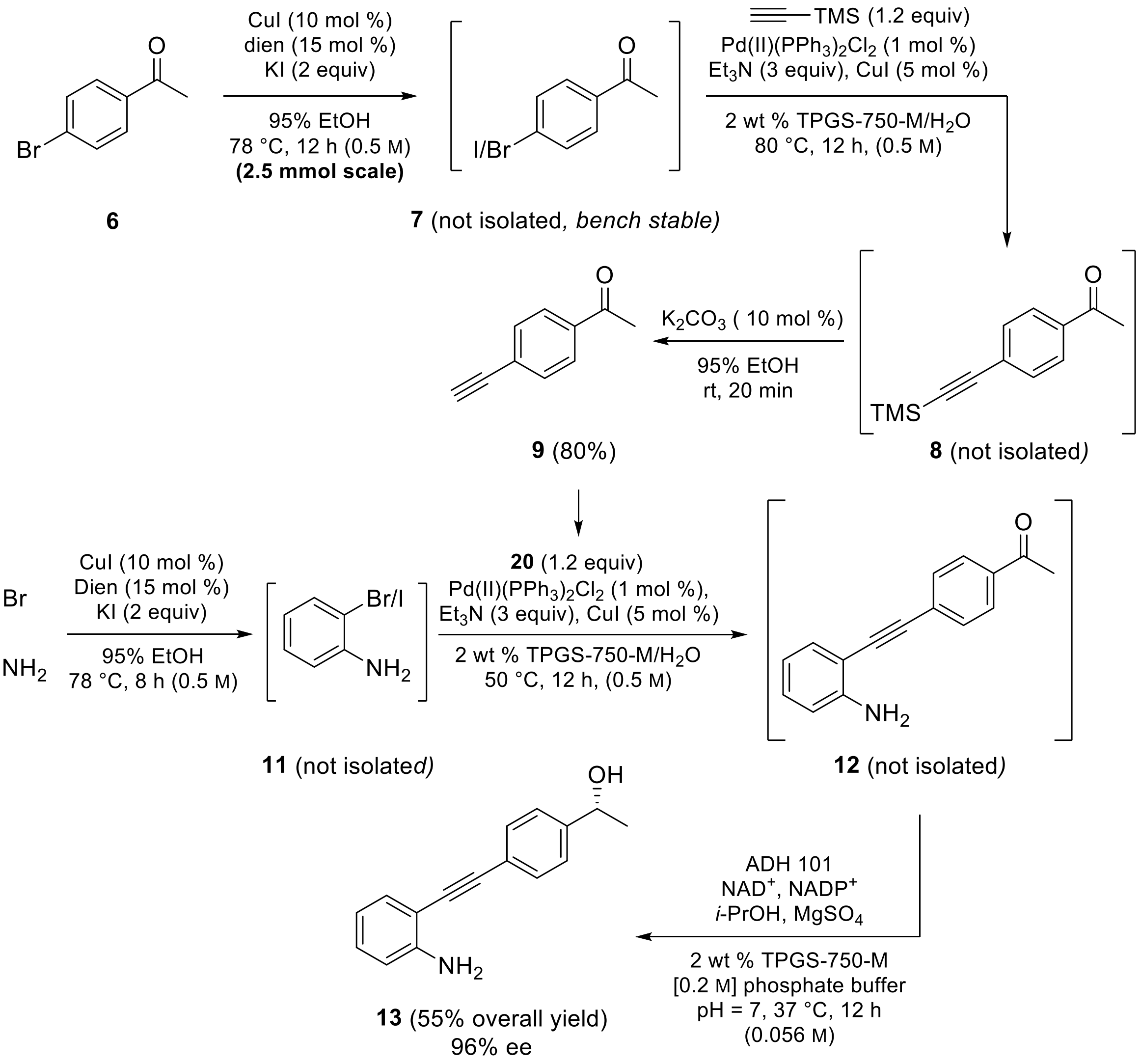

3-Step, 1-pot sequence procedure: synthesis of 4'-ethnynylacetophenone **(13)**

Iodination: To an oven dried 2 dram vial, 4'-bromoacetophenone (**6**) (500.0 mg, 2.5 mmol, 1 equiv), KI (830.0 mg, 5 mmol, 2.0 equiv) were added along with a ptfe-coated magnetic spin vane. The vial was then transferred to an argon-filled glove box where CuI (48.0 mg, 0.25 mmol, 0.1 equiv) was added. The vial was then capped with a rubber septum and removed from the glove box. Through the septum, 500 µL of de-gassed THF as co-solvent was added followed by diethylenetriamine (40 µL, 0.375 mmol, 0.15 equiv) as a stock solution in 4.5 mL of 95% ethanol that was rigorously degassed with Ar. The reaction was sealed with PTFE-tape and placed into a preheated aluminum block reactor set to 83 °C (internal temperature 78 °C) and left to react for 8 h. Upon completion, the vial was allowed to come to rt and the solvent removed under vacuum. The crude material was suspended in 5 mL of EtOAc and filtered over a plug of silica. The plug

was washed with EtOAc and solvent removed to yield **7**, which was stored on the bench wrapped in foil until further use (approximately 1 week).

1st Sonogashira coupling: To a 2-dram vial containing **7**, was added *bis*-triphenylphosphine palladium(II)Cl_2 (18.0 mg, 0.025 mmol, 0.01 equiv) and the vial brought into an argon-filled glove box where CuI (24.0 mg, 0.125 mmol, 0.05 equiv) was added and the vial capped with a rubber septum. The vial was removed from the glove box and through the septum, de-gassed triethylamine (759.0 mg, 1050 µL, 3 equiv), 4 mL de-gassed 2 wt % TPGS-750-M/H_2O, and TMS-acetylene (294.7 mg, 427 µL, 3 mmol, 1.2 equiv) were added sequentially. The vial was then placed in a pre-heated aluminum heating block over a stir plate with thermocouple probe in the aluminum block set to 55 °C to give an internal vial temperature of 50 °C for 12 h.

Deprotection: The reaction was allowed to cool to rt and the reaction extracted with 3 x 5 mL portions of EtOAc. The solvent was removed to yield an oily residue which was dissolved in 25 mL of 95% EtOH [0.1 M] and then added anhydrous K_2CO_3 (34.5 mg, 0.25 mmol, 0.1 equiv) and left to react for 20 min until all the intermediate **8** was consumed. The reaction was dried directly onto silica and purified by flash column chromatography (10% EtOAc/90% hexanes, R_f: 0.40) and dried under vacuum to yield **9** as a light yellow powder (288.3 mg, 80%). Spectral data matched those found in literature. [27]

3-Step, 1-pot sequence procedure: synthesis of 1-(4-((2-aminophenyl)ethynyl)phenyl)ethan-1-ol (**13**)

Iodination: To an oven dried 1 dram vial, 2-bromoaniline (43.0 mg, 0.25 mmol, 1 equiv), KI (83.0 mg, 0.5 mmol, 2.0 equiv) were added along with a ptfe-coated magnetic stir bar. This was then transferred to an argon-filled glove box where CuI (4.8 mg, 0.025 mmol, 0.1 equiv) was added. The vial was then capped with a rubber septum and removed from the glove box. Through the septum, 50 µL of de-gassed THF as co-solvent was added followed by diethylenetriamine (4 µL, 0.0375 mmol, 0.15 equiv) as a stock solution in 0.45 mL of 95% ethanol that was rigorously degassed with Ar. The reaction was sealed with PTFE-tape and placed into a preheated aluminum block reactor set to 83 °C (internal temperature 78 °C) and left to react for 8 h. Upon completion, the vial was allowed to come to rt and the solvent removed under vacuum.

2nd Sonogashira coupling: Upon completion, the vial was removed from the heating block and allowed to cool to rt. The ethanol was removed under vacuum and *bis*-triphenylphosphine palladium(II)Cl_2 (1.8 mg, 0.0025 mmol, 0.01 equiv) and alkyne **9** (43.2 mg, 0.3 mmol, 1.2 equiv) were added to the crude residue and the vial brought into an argon-filled glove box where CuI (2.4 mg, 0.0125 mmol, 0.05 equiv) was added and the vial capped with a rubber septum. The vial was removed from the glove box and through the septum, de-gassed triethylamine (75.9 mg, 105 µL, 3 equiv), 0.4 mL de-gassed 2 wt % TPGS-750-M/H_2O, and 4-ethynylanisole (40.0 mg, 0.3 mmol, 39 µL) were added sequentially. The vial was then placed in a pre-heated aluminum heating block

over a stir plate with thermocouple probe in the aluminum block set to 55 °C to give an internal vial temperature of 50 °C for 12 h.

Reduction: The reaction was allowed to cool to rt and the concentration adjusted to [0.056 M] by adding 3.9 mL of a 2 wt % TPGS-750-M/buffer solution (phosphate [0.2 M], pH = 7). Anhydrous $MgSO_4$ (1.0 mg), NAD+ (3.1 mg), NADP+ (3.0 mg), i-PrOH (0.6 mL), and ADH101 (25 mg) were added in succession.[28] The reaction was stirred at 37 °C for 16 h and the reaction extracted with 4 x 1 mL of EtOAc. The combined organic fractions were dried onto silica and isolated by flash column chromatography (30% EtOAc/70% hexanes, R_f: 0.35, dark yellow spot) and dried under vacuum to afford product **13** as a dark, yellow crystalline solid. (70.8 mg, 55% yield, 96% ee).

^{1}H NMR (500 MHz, $CDCl_3$) δ 7.56 – 7.46 (m, 2H), 7.36 (dd, *J* = 8.0, 1.9 Hz, 3H), 7.14 (ddd, *J* = 8.5, 7.4, 1.6 Hz, 1H), 6.80 – 6.63 (m, 2H), 4.92 (q, *J* = 6.5 Hz, 1H), 1.50 (d, *J* = 6.5 Hz, 3H).

^{13}C NMR (126 MHz, $CDCl_3$) δ 147.8, 146.0, 132.2, 131.6, 129.8, 125.5, 122.4, 118.1, 114.4, 114.4, 108.0, 94.6, 85.8, 70.1, 25.2.
HRMS (ESI-GC-MS) *m/z*: $[M-H_2O]^+$ calcd. for $C_{16}H_{13}N$: 219.1048; found: 219.1039 (4.1 ppm)

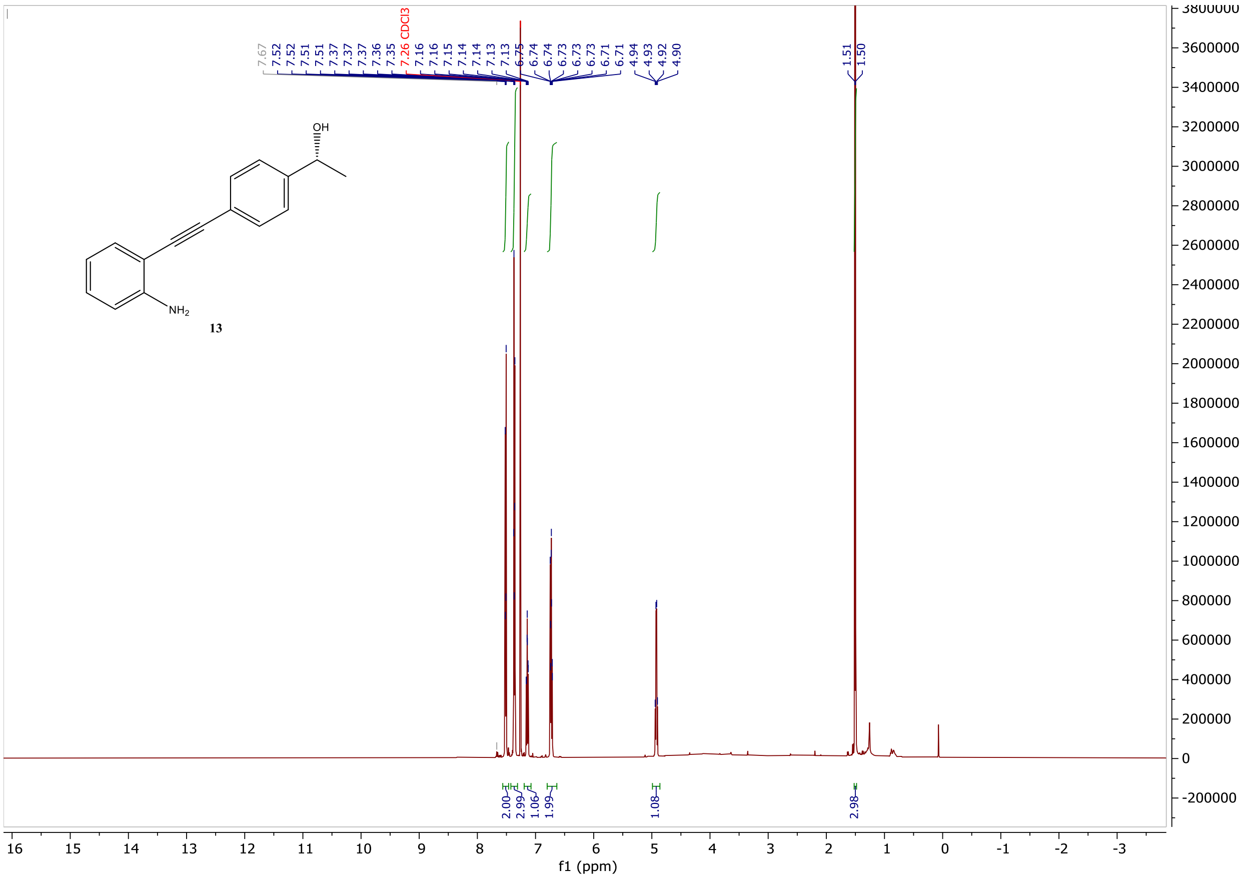

Figure S12. ^{1}H NMR spectrum of **13** in $CDCl_3$

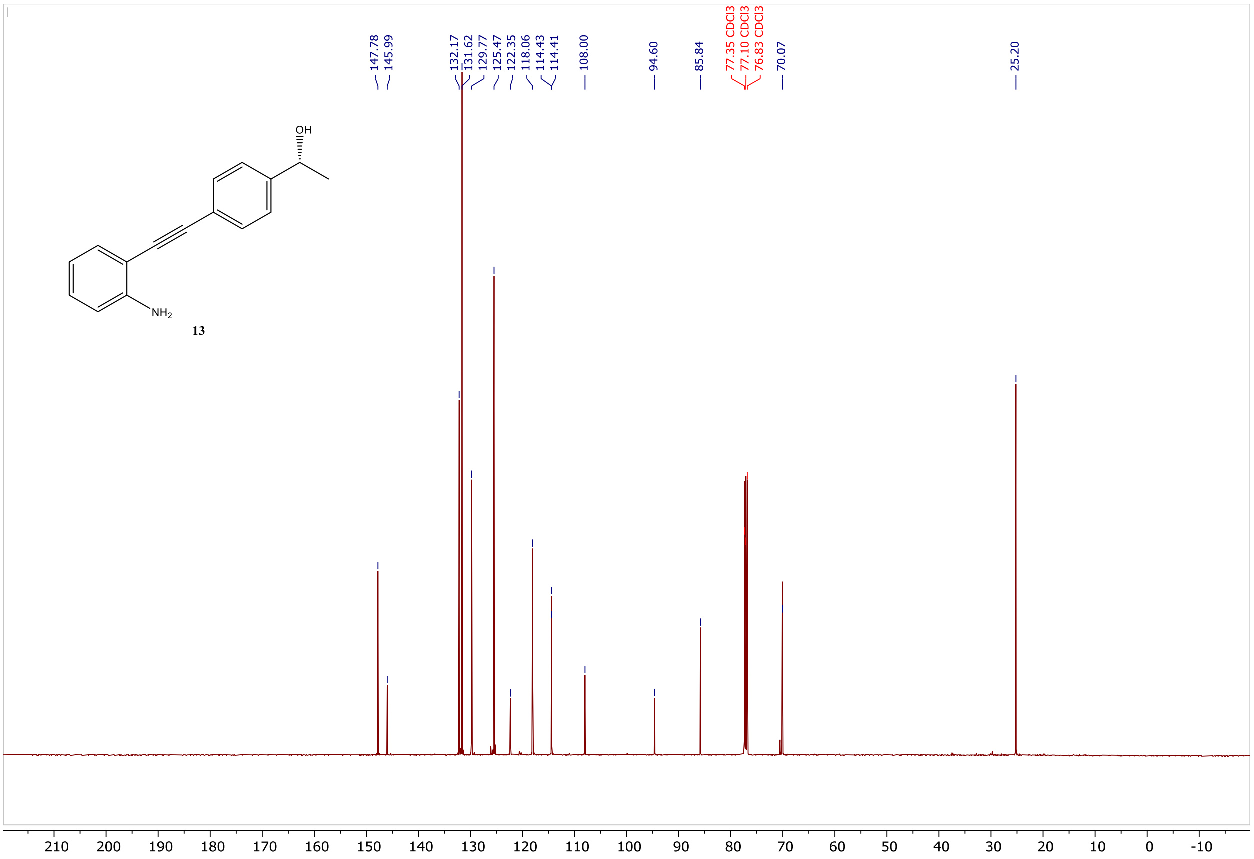

Figure S13. ^{13}C NMR spectrum of **13** in $CDCl_3$

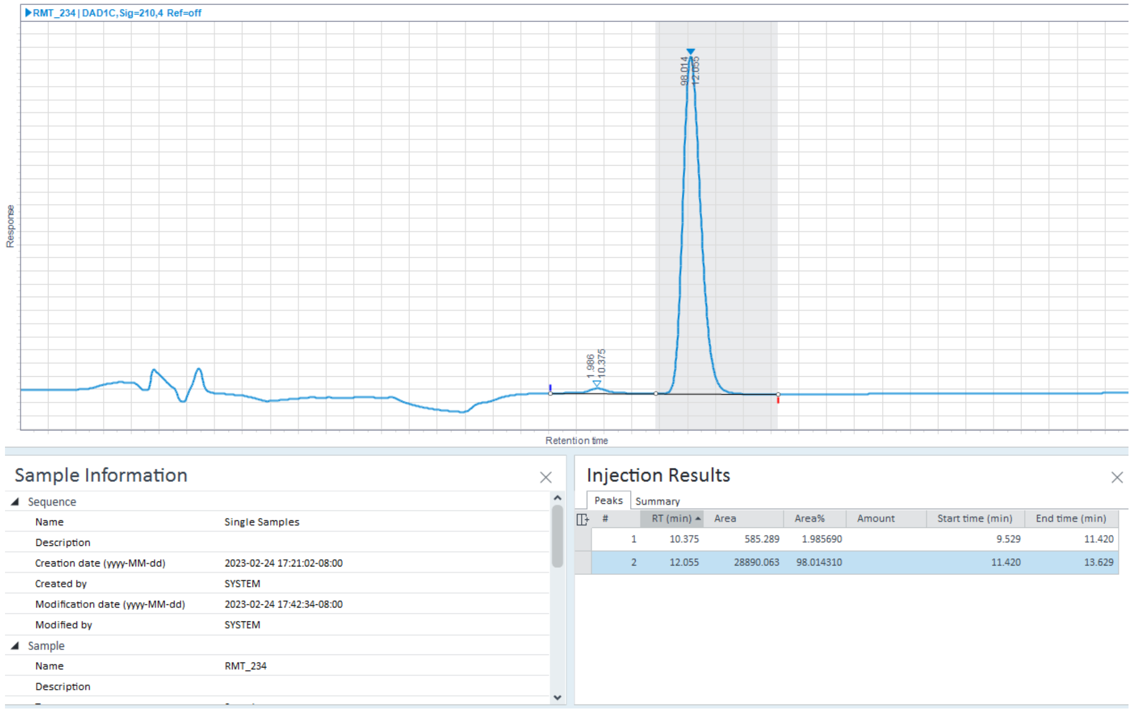

#	RT (min)	Area	Area%	Amount	Start time (min)	End time (min)
1	10.375	585.289	1.985690		9.529	11.420
2	12.055	28890.063	98.014310		11.420	13.629

Figure S14. Chiral HPLC of **13**

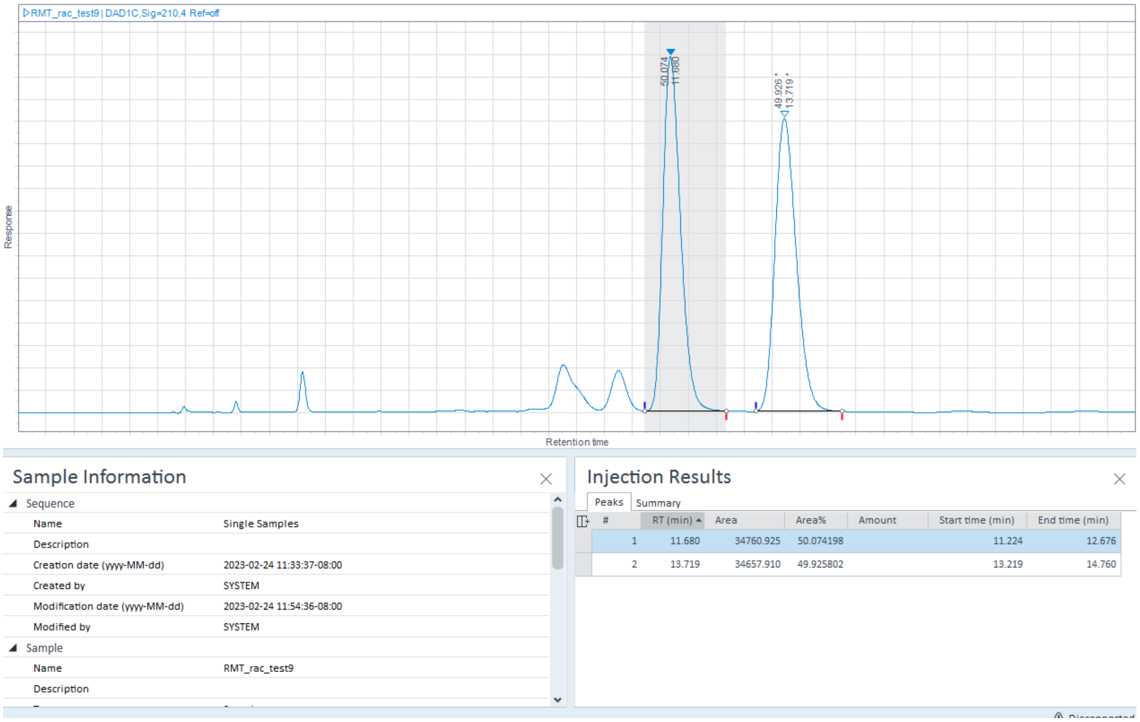

#	RT (min)	Area	Area%	Amount	Start time (min)	End time (min)
1	11.680	34760.925	50.074198		11.224	12.676
2	13.719	34657.910	49.925802		13.219	14.760

Figure S15. Chiral HPLC on *rac*-**13**

Screening of Sensitive Substrates

4-Iodoquinoline

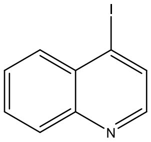

Following the General Procedure 1, 4-Bromoquinoline (52 mg, 0.25 mmol) was converted into 4-Iodoquinoline. Filtration provided the mixture of starting material and product (Br: 66%, I: 44%) by q^{1}H NMR. ^{1}H NMR (500 MHz, CDCl3) δ **8.68 (d, *J* = 4.6 Hz, 0.31H)**, **8.46 (d, *J* = 4.5 Hz, 0.21H)**, 8.28 – 8.15 (m, 0H), 8.14 – 8.08 (m, 0H), 8.07 – 7.98 (m, 1H), 7.86 – 7.76 (m, 0H), 7.79 – 7.72 (m, 0H), 7.71 (d, *J* = 4.6 Hz, 0H), 7.69 – 7.63 (m, 0H), 7.66 – 7.58 (m, 0H), 6.09 (s, 1H), 5.30 (s, 0H), 3.77 (s, 3H), 3.72 (q, *J* = 7.0 Hz, 0H), 3.49 (s, 2H), 1.24 (t, *J* = 7.0 Hz, 1H).

Br (lit.)[29] : ^{1}H NMR (400 MHz, CDCl3): δ **8.68 (d, J = 4.7 Hz, 1H)**, 8.20 (dd, J = 8.4, 1.4 Hz, 1H), 8.13 (dd, J = 8.4, 1.4 Hz, 1H), 7.78 (ddd, J = 8.4, 7.0, 1.4 Hz, 1H), 7.72 (d, J = 4.7 Hz, 1H), 7.66 (ddd, J = 8.4, 7.0, 1.4 Hz, 1H)

I (lit.) [30]: ^{1}H NMR ($CDCl_3$) *δ* **8.32 (d, 1H, *J*=4.3 Hz, 2H)**, 7.92 (d, 1H, *J*=7.1 Hz), 7.88 (d, 1H, *J*=8.3 Hz), 7.84 (d, 1H, *J*=4.3 Hz), 7.61 (m, 1H), 7.48 (m, 1H).

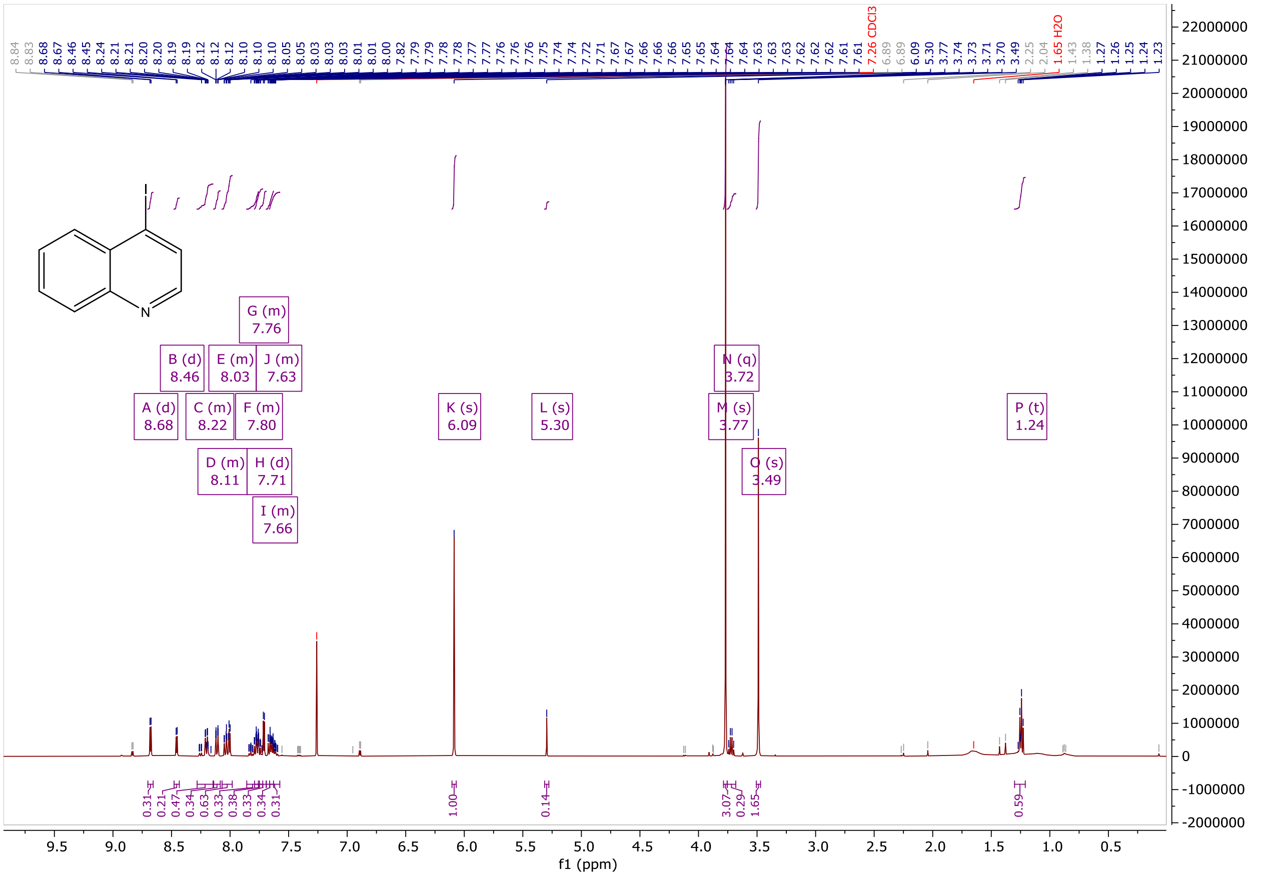

Figure S16. ^{1}H NMR of crude iodination of 4-bromoquinoline to 4-iodoquinoline

3. Large-Scale Reaction Procedures

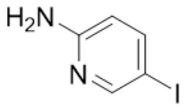

Gram scale synthesis of 5-iodopyridin-2-amine (20)

To a round bottomed flask with a sidearm and ptfe-stopcock was charged with a ptfe-coated magnetic stir bar, 5-bromopyridin-2-amine (1.0 g, 5.8 mmol, 1.0 equiv) and KI (1.919 g, 11.6 mmol, 2.0 equiv). The flask was fitted with a reflux condenser and the system was evacuated and backfilled with argon via Schlenk manifold 3 times. To a 2-dram vial equipped with a stir bar was added, in an argon-filled glove box, CuI (110 mg, 0.58 mmol, 0.1 equiv) and sealed with a rubber septum. This vial was then charged with 6.0 mL of degassed 95% ethanol and dien (92.8 uL, 0.87 mmol, 0.15 equiv). The round bottomed flask was charged with 1.0 mL de-gassed THF, followed by the solution of catalyst and then an additional 6.0 mL of de-gassed 95% ethanol. This reaction was lowered into an oil bath at 83 °C and left to reflux for 12 h. Upon completion, this material was left to cool to rt and then the solvent was removed under vacuum. The crude material was filtered over silica and re-submitted to the same conditions for a second pass. After the second filtration the reaction yielded 904.1 mg of material (73% yield, 4:1 I:Br) as a cakey, yellow powder.

4. Optimization Studies

All optimization study analysis was performed by GC-MS conversions using a Shimadzu-GCMS-QP2020NX system with a GC-2030 front end and a J&W HP-5ms column (30 m, 0.25 mm, 0.25 µm film). All conversions are presented as a percentage of total peak areas of bromide, iodide, and de-halogenated by-products.

Screening of ligands

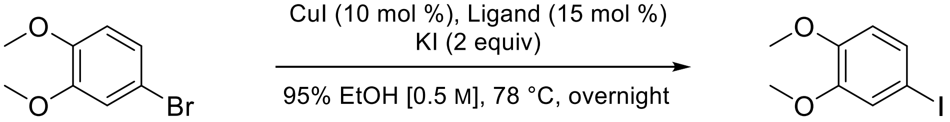

	Ligand	Conversion to iodide (%)		Ligand	Conversion to iodide (%)
**L1**	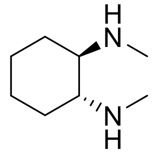	0	**L8**	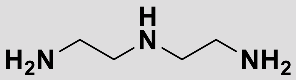	**71**
**L2**	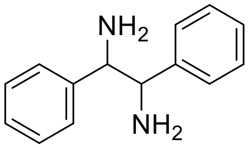	0	**L9**	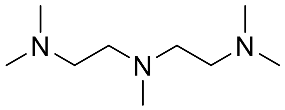	0
**L3**	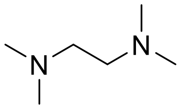	0	**L10**	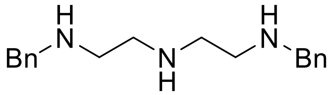	74
**L4**	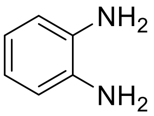	0	**L11**	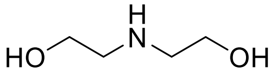	14
**L5**	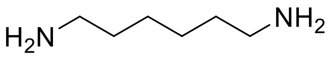	0	**L12**	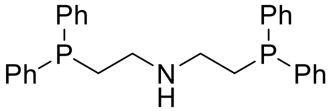	0
**L6**	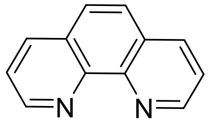	0	**L13**	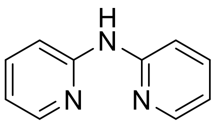	17
**L7**	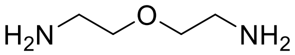	0	**L14**	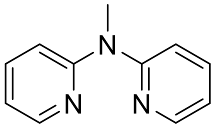	0

Table S1. Screening of Ligands

Solvent screening

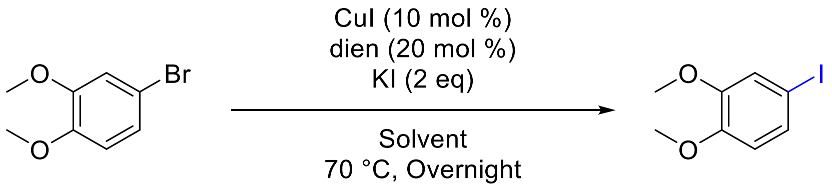

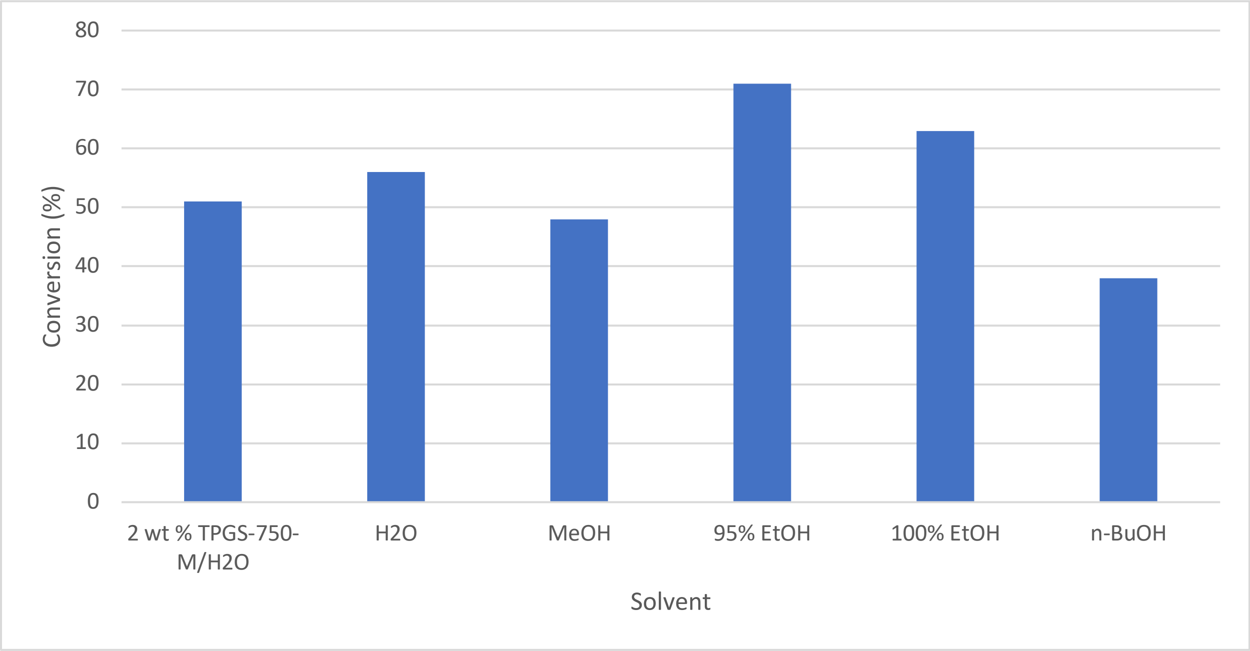

Table S2. Screening of Solvents

Screening of ligand concentration

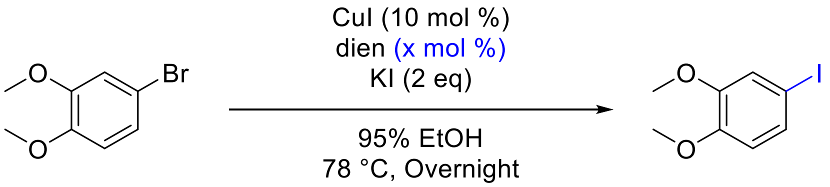

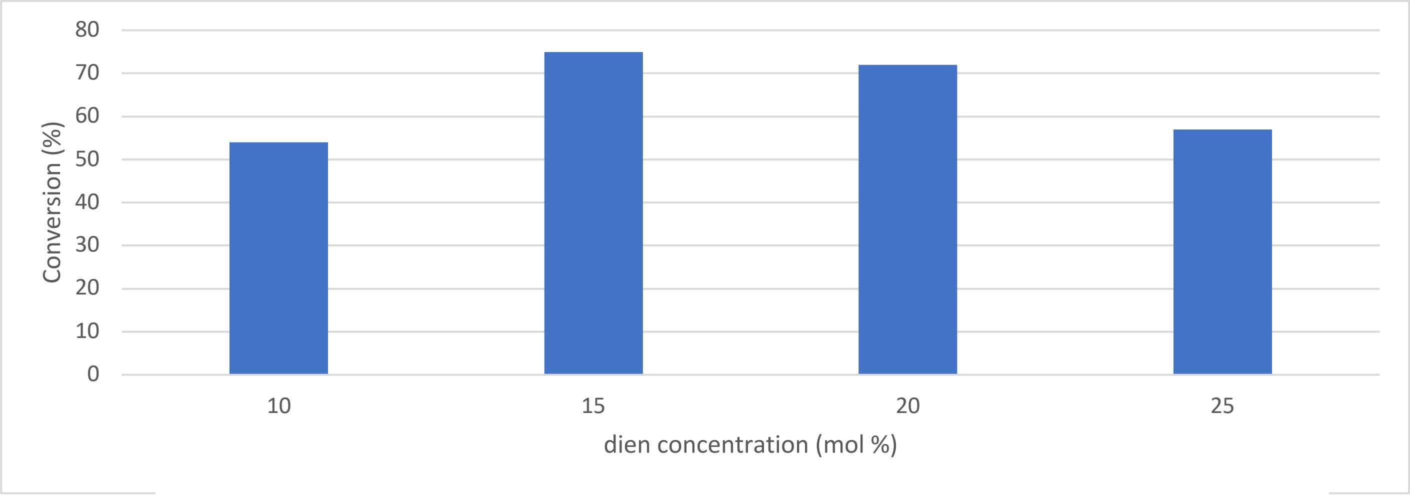

Table S3. Screening of Ligand Concentration

Screening of iodide sources

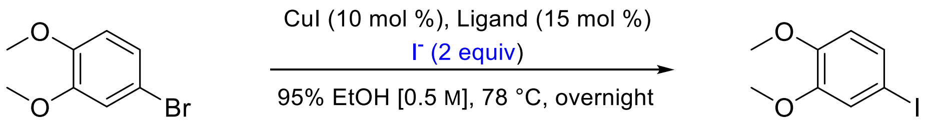

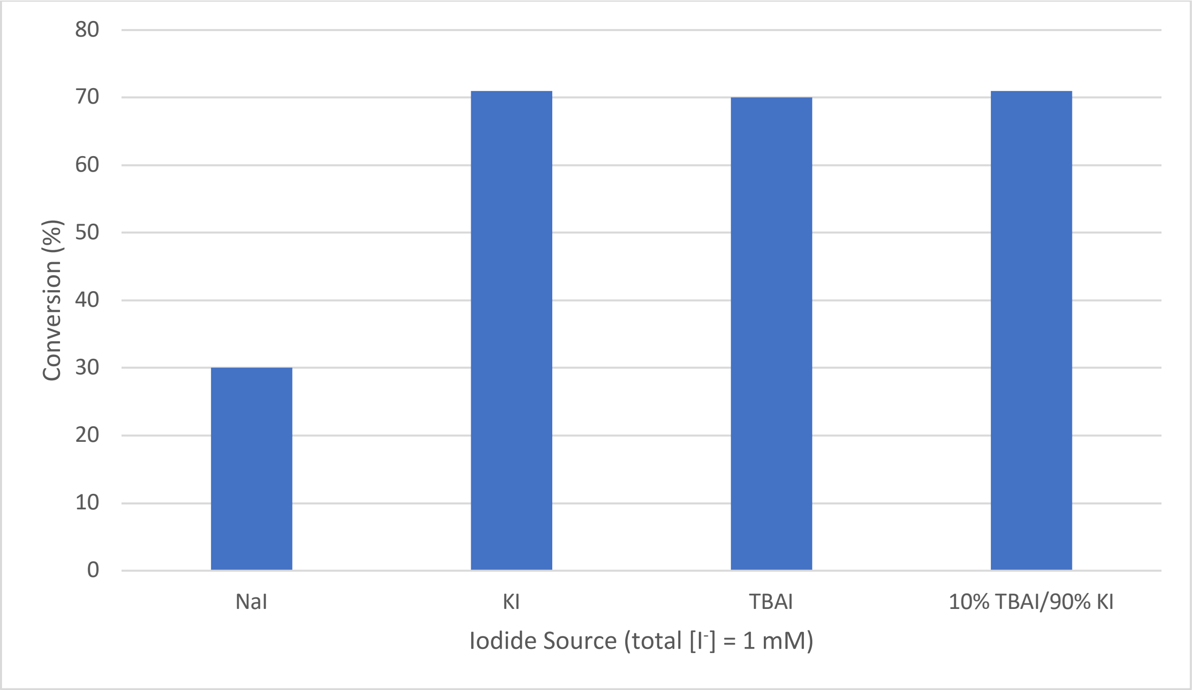

Table S4. Screening of Iodide Sources

Screening of copper sources

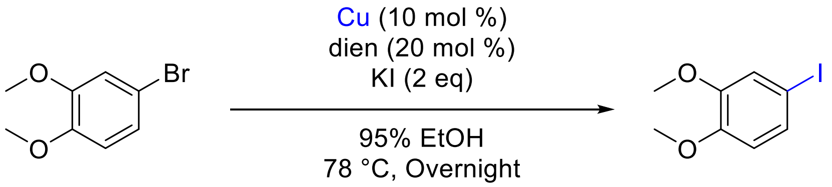

copper source	conversion (%)
Cu(I)I	74
$Cu(I)_2O$	72
Cu(I)OTf	48
Cu(I)Br	40
Cu(I)CN	53
$Cu(II)Br_2$	49
$Cu(II)OTf_2$	0

Table S5. Screening of Copper Sources

Screening of copper loadings

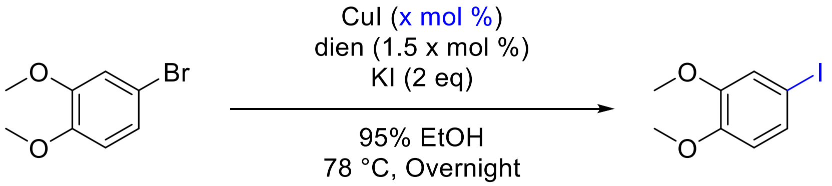

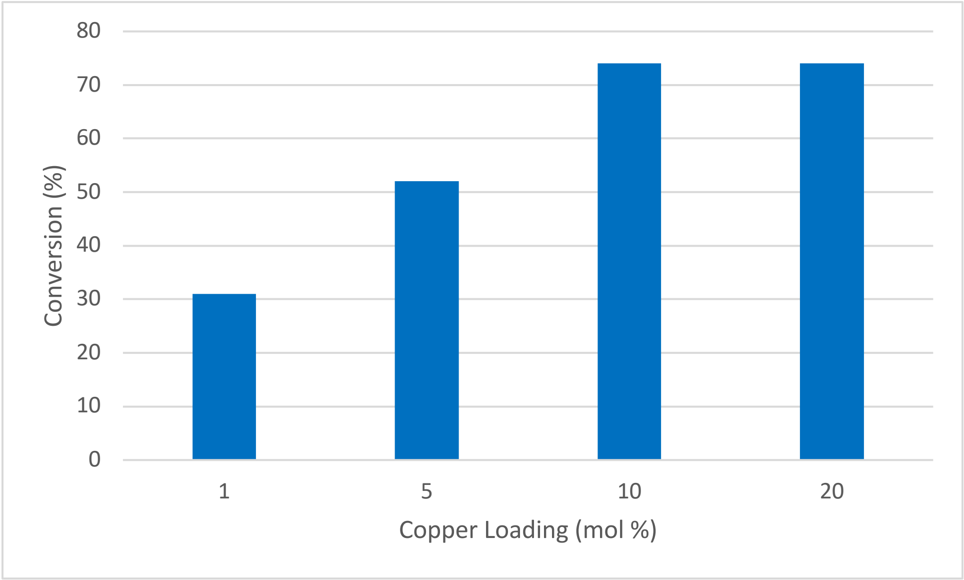

Table S6. Screening of Copper Loadings

5. General Procedures

General Procedure 1 for iodinations:

A 1-dram glass vial was charged with substrate (0.5 mmol), and if a solid, KI (166 mg, 1.0 mmol, 2.0 equiv) and a ptfe-coated magnetic stir bar. The vial was then transferred to an argon-filled glove box where CuI (9.6 mg, 0.05 mmol, 0.1 equiv) was added. The vial was then capped with a rubber septum and removed from the glove box. Through the septum, substrate, if liquid, was added followed by 100 µL of de-gassed THF as co-solvent, if necessary, followed by diethylene triamine (8 µL, 0.075 mmol, 0.15 equiv) as a stock solution in 0.9 mL 95% ethanol that was rigorously degassed with Ar. The reaction was sealed with PTFE-tape and placed into a pre-heated aluminum block reactor set to 83 °C (internal temperature 78 °C) and left to react for 8 h.

Upon completion, the reaction was left to cool and then the solvent removed under vacuum. The remaining wet solids were suspended in 1.0 mL EtOAc and subsequently filtered through ~1 cm pad of silica. The silica pad was subsequently flushed with 2 to 3 portions of EtOAc and the solvent then removed under vacuum to yield crude material for further coupling or left in solution for q^{1}H NMR.

General Procedure 2 for iodinations:

A 1-dram glass vial was charged with substrate (0.5 mmol), KI (166 mg, 1.0 mmol, 2.0 equiv), and a ptfe-coated magnetic stir bar. This was then transferred to an argon-filled glove box where CuI (9.6 mg, 0.05 mmol, 0.1 equiv) was added. The vial was then capped with a rubber septum and removed from the glove box. Through the septum, 100 µL of de-gassed triethylamine as co-solvent was added followed by diethylenetriamine (8 µL, 0.075 mmol, 0.15 equiv) as a stock solution in 0.9 mL of 95% ethanol that was rigorously degassed with Ar. The reaction was sealed with PTFE-tape and placed into a preheated aluminum block reactor set to 83 °C (internal temperature 78 °C) and left to react for 8 h.

Upon completion, the reaction was left to cool and then the solvent removed under vacuum. 1 mL of 1 M HCl solution was added and the reaction extracted with 3 x 1 mL portions of EtOAc and left in solution for q^{1}H NMR.

General Procedure 3 for two-pass iodinations:

Two 1-dram glass vials were each charged with substrate (0.5 mmol), KI (166 mg, 1.0 mmol, 2.0 equiv) and a ptfe-coated magnetic stir bar. These were then transferred to an argon-filled glove box where CuI (9.6 mg, 0.05 mmol, 0.1 equiv) was added. The vials were then capped with a rubber septum and removed from the glove box. Through the septum, 100 µL of de-gassed THF as co-solvent was added followed by diethylenetriamine (8 µL, 0.075 mmol, 0.15 equiv) as a stock solution in 0.9 mL of 95% ethanol that was rigorously degassed with Ar. The reactions were sealed with PTFE-tape and placed into a preheated aluminum block reactor set to 83 °C (internal temperature 78 °C) and left to react for 8 h.

Upon completion, the reactions were left to cool and then the solvent removed under vacuum and the residue suspended in 1mL of ethyl acetate. These were then filtered through a small plug of silica back into the same 1-dram vials. The silica was washed with 2 x 1mL ethyl acetate portions and one reaction was set aside for 1st pass q^{1}H NMR yield analysis. To the remaining vial, the solvent was removed under vacuum and to this crude mixture KI (166 mg, 1.0 mmol, 2.0 eq) was added and the reaction transferred to an argon-filled glove box. CuI (9.6 mg, 0.05 mmol, 0.1 eq) was added and the vial was then capped with a rubber septum and removed from the glove box. Through the septum, 100 µL of de-gassed THF as co-solvent was added, if necessary, followed by diethylenetriamine (8 µL, 0.075 mmol, 0.15 equiv) as a stock solution in 0.9 mL of 95% ethanol that was rigorously degassed with Ar. The reaction was sealed with PTFE-tape and placed into a preheated aluminum block reactor set to 83 °C (internal temperature 78 °C) and left to react for 8 h.

Determination of yields of iodide formed, by q^{1}H NMR of crude reaction material

Yields of newly formed iodides were determined using 1, 3, 5-trimethoxybenzene as internal standard (^{1}H NMR ($CDCl_3$) δ 6.09 (s, 3H), 3.77 (s, 3H)). Unless otherwise specified, 28.0 mg (0.167 mmol) was accurately added to each crude product mixture after silica filtration. This was then dissolved into ethyl acetate and mixed until dissolved. An aliquot of this mixture was evaporated to dryness and submitted for NMR analysis. Yields were obtained relative to the normalized integration of the internal standard peak at 6.09 ppm as 1 for a 0.5 mmol scale reaction.

^{1}H NMR spectra of starting materials and crude reaction mixtures are supplied in their totality as well as stacked, zoomed in spectra of relevant regions to observe the shift in peaks. Relevant peaks are emboldened in the reported spectra. Peaks associated with the internal standard are underlined in the line listings of the ^{1}H NMR spectral data. Where available, published spectral data and their sources are provided.

6. Characterization data

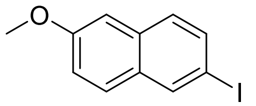

2-Iodo-6-methoxynaphthalene (14)

Following the General Procedure 1, 2-bromo-6-methoxynapthalene (118.5 mg, 0.5 mmol) was converted into 2-iodo-6-methoxynapthalene. Filtration provided the mixture of starting material and **14** (Br: 16%, I: 77%) by q^{1}H NMR. ^{1}H NMR (500 MHz, $CDCl_3$) δ **8.14 (d, *J* = 1.7 Hz, 0.77H)**, **7.92 (d, *J* = 2.0 Hz, 0.16H)**, 7.69 –7.58 (m, 2H), 7.53 – 7.45 (m, 1H), 7.15 (td, *J* = 9.6, 2.6 Hz, 1H), 7.09 (dd, *J* = 9.4, 2.5 Hz, 1H), <u>6.09 (s, 1H)</u>, 3.92 (d, *J* = 1.1 Hz, 3H), <u>3.77 (s, 3H)</u>.

Br (lit.)[1] : ^{1}H NMR ($CDCl_3$) *δ* **7.91 (d, J¼1.7 Hz, 1H),** 7.62 (m, 2H), 7.49 (dd, J1¼2.0 Hz, J2¼8.7 Hz, 1H), 7.16 (dd, J1¼2.5 Hz, J2¼8.9 Hz, 1H), 7.09 (d, J¼2.5 Hz, 1H), 3.91 (s, 3H)

I (lit.)[2] : ^{1}H NMR ($CDCl_3$) *δ* **8.14 (s, 1H)**, 7.68–7.46 (m, 3H), 7.25–7.07 (m, 2H), 3.92 (s, 3H)

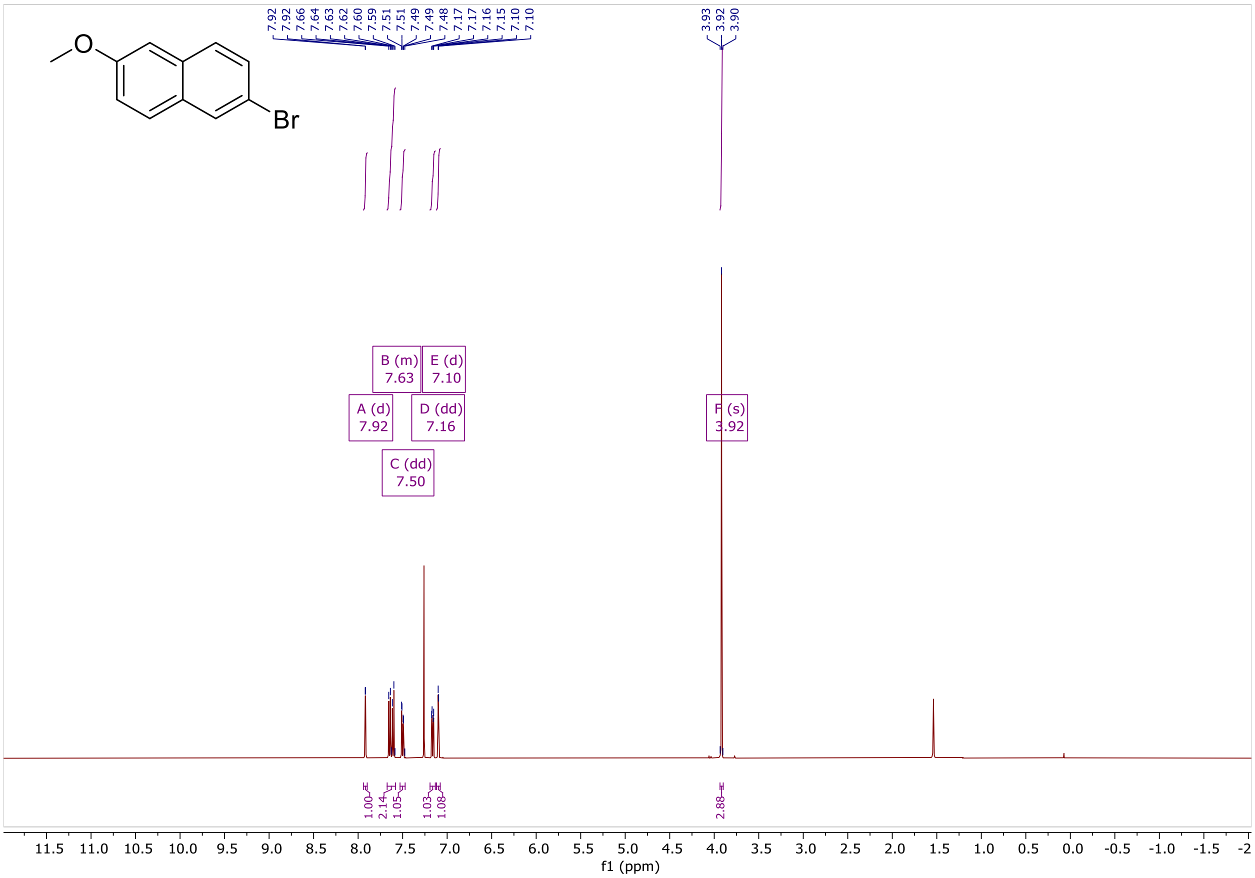

Figure S17. ^{1}H NMR spectrum of **2-bromo-6-methoxynapthalene** in $CDCl_3$

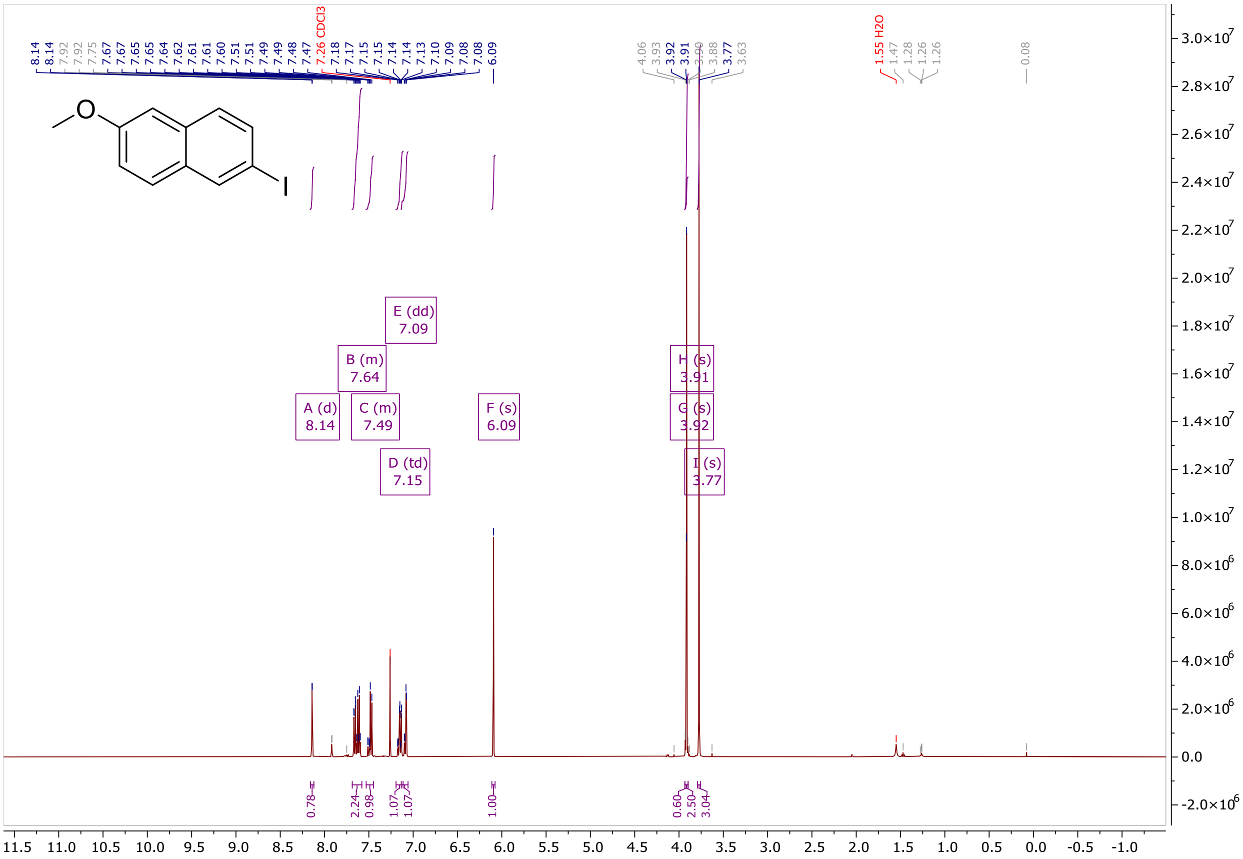

Figure S18. ^{1}H NMR spectrum of **14** in $CDCl_3$

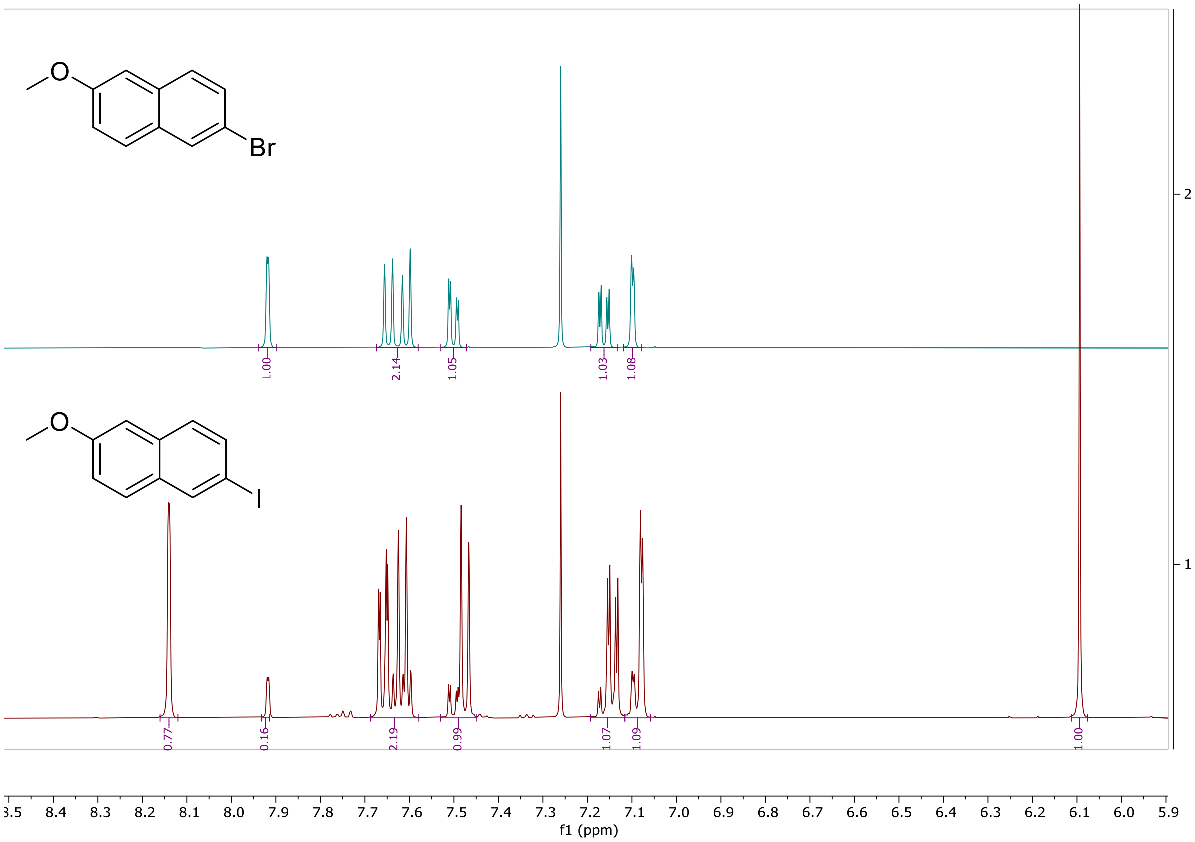

Figure S19. Overlaid ^{1}H NMR spectrum of **2-bromo-6-methoxy naphthalene** and **14** in $CDCl_3$

2-Iodoaniline (15)

Following the General Procedure 1, 2-bromoaniline (86.0 mg, 0.5 mmol) was converted into 2-iodoaniline. Filtration provided the mixture of starting material and **15** (Br: 18%, I: 72%) according to q^{1}H NMR with ethylene carbonate (^{1}H NMR: δ 4.51 (s, 4H)) as internal standard.
A second experiment following General Procedure 1 except without degassing the solvent was performed and the yield was unaffected. ^{1}H NMR (500 MHz, $CDCl_3$) δ 7.63 (dd, *J* = 7.9, 1.4 Hz, 0.95H), 7.40 (dd, *J* = 8.0, 1.4 Hz, 0.16H), 7.15 – 7.07 (m, 1.09H), 6.75 (dd, *J* = 7.9, 1.5 Hz, 1.08H), **6.61 (td, *J* = 7.6, 1.5 Hz, 0.18H)**, **6.48 – 6.45 (m, 0.86H)**, 4.51 (d, *J* = 2.9 Hz, 4H), 4.13 – 4.05 (m, 2H), 1.29 (s, 0H), 1.25 (s, 0H), 0.86 (s, 0H).

Br (lit.)[3]: ^{1}H NMR (400 MHz, $CDCl_3$) δ 7.41 (dd, J = 8.0, 1.4 Hz, 1H), 7.11 (ddd, J = 8.3, 7.4, 1.4 Hz, 1H), 6.77 (dd, J = 8.0, 1.5 Hz, 1H), **6.62 (td, J = 7.6, 1.5 Hz, 1H)**, 3.87 (s, 2H).

I (lit)[4]: ^{1}H NMR (400 MHz, $CDCl_3$) δ 7.6 (d, J = 7.9 Hz, 1H), 7.1 (t, J = 7.6 Hz, 1H), 6.8 (d, J = 8.0 Hz, 1H), **6.5 (t, J = 7.6 Hz, 1H)**, 4.1 (s, 2H).

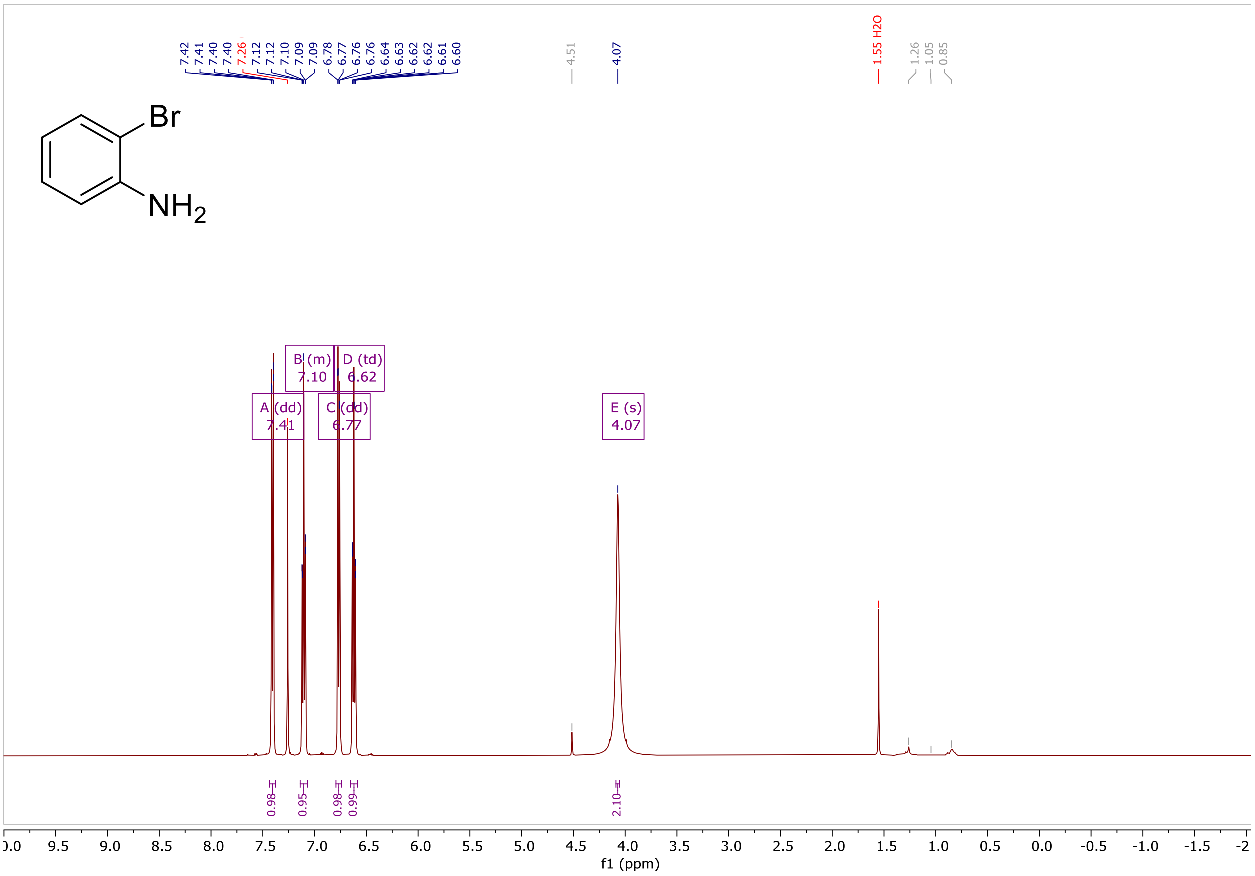

Figure S20. ^{1}H NMR spectrum of **2-bromoaniline** in $CDCl_3$

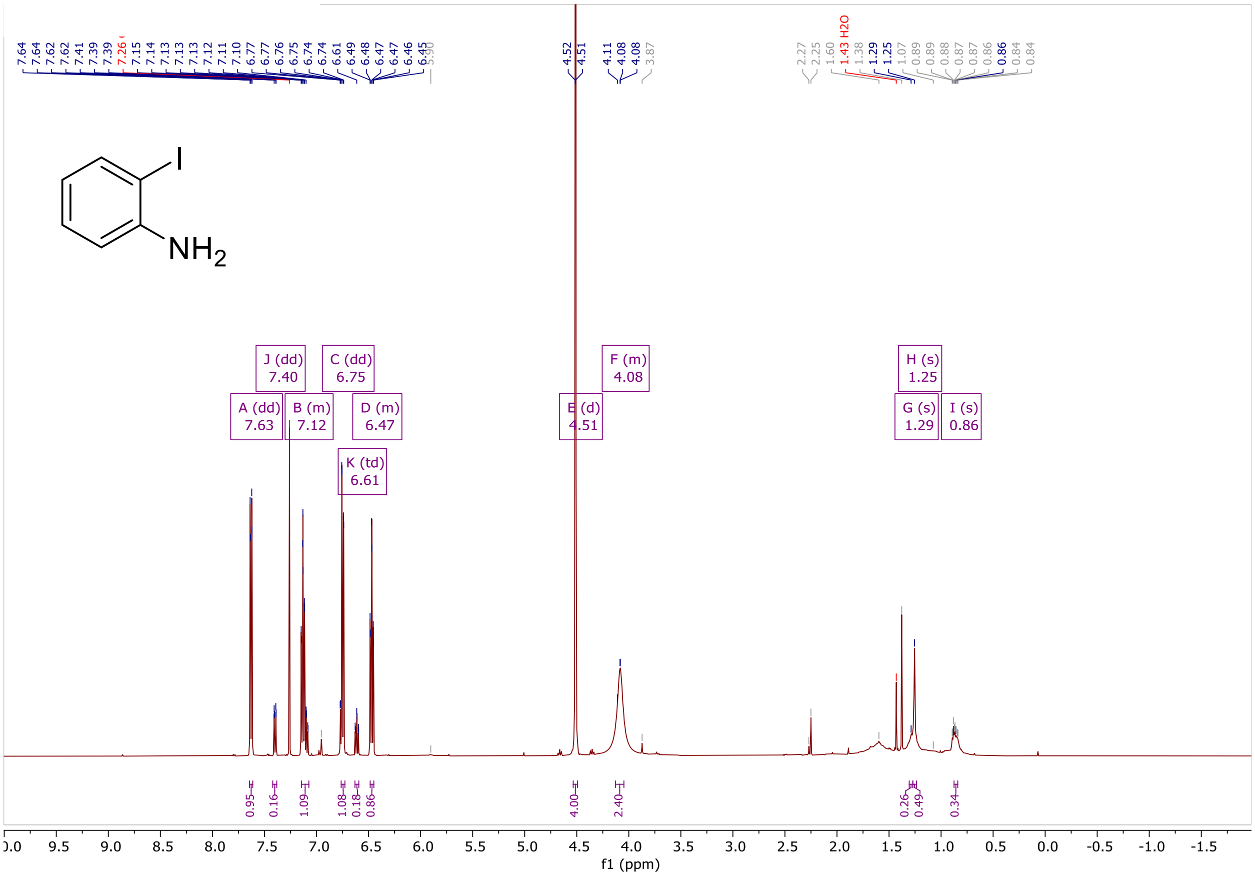

Figure S21. ^{1}H NMR spectrum of **15** in $CDCl_3$

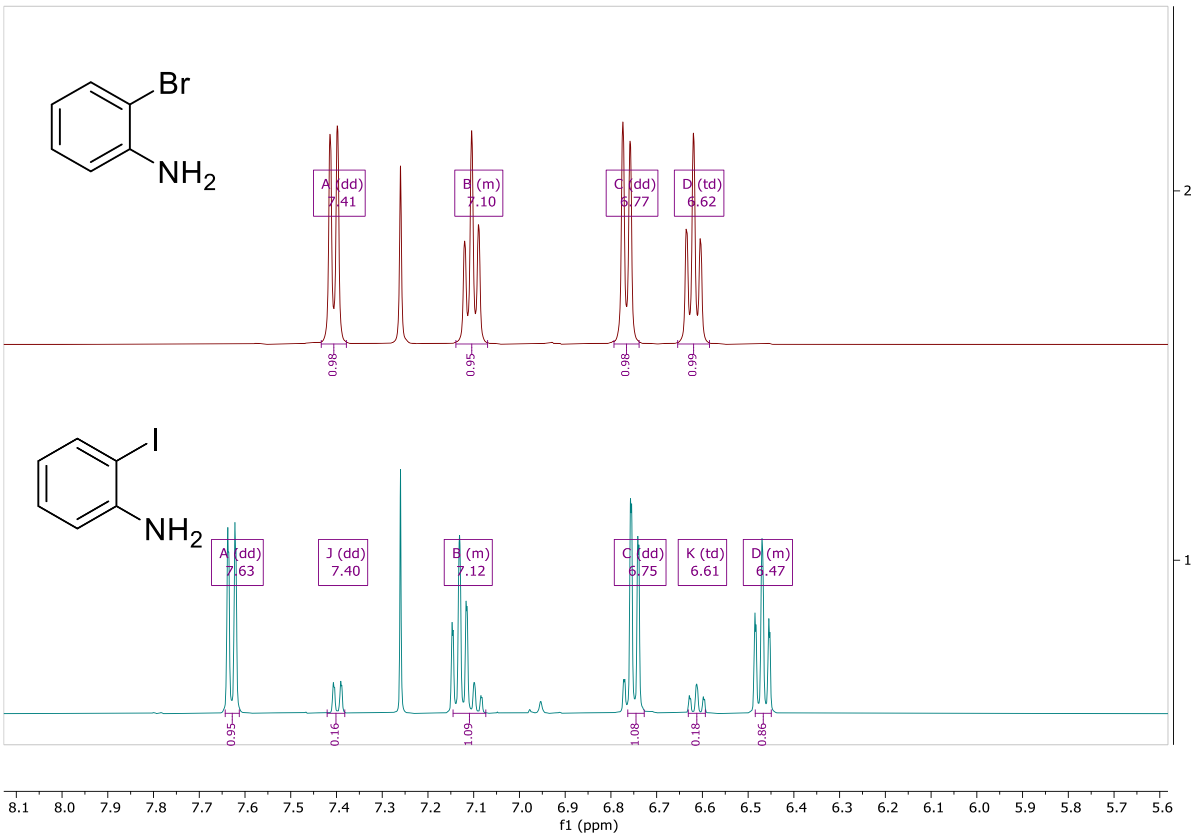

Figure S22. Overlaid ^{1}H NMR spectrum of **2-bromoaniline** and **15** in $CDCl_3$

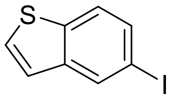

5-Iodo-benzo[b]thiophene (16)

5-Bromobenzo[b]thiophene (53.3 mg, 0.25 mmol) was converted into 5-iodobenzo[b]thiophene by a modified General Procedure 1. 50 µL of THF was used as co-solvent and 4 µL of diethylene triamine ligand in 450 µL of degassed 95% ethanol was used to initiate the reaction. Filtration provided the mixture of starting material and **16** (Br: 50%, I: 50%) according to q^{1}H NMR. ^{1}H NMR (500 MHz, $CDCl_3$) δ **8.14 (d, *J* = 1.7 Hz, 0.28H), 7.92 (d, *J* = 2.0 Hz, 0.25H)**, 7.69 – 7.58 (m, 2H), 7.53 – 7.45 (m, 1H), 7.15 (td, *J* = 9.6, 2.6 Hz, 1H), 7.09 (dd, *J* = 9.4, 2.5 Hz, 1H), <u>6.09 (s, 1H)</u>, 3.92 (d, *J* = 1.1 Hz, 3H), <u>3.77 (s, 3H)</u>.

Br (lit.)[5] : ^{1}H NMR (500 MHz, $CDCl_3$) δ **7.97-7.95 (m, 1H)**, 7.75-7.71 (m, 1H), 7.48-7.46 (m, 1H), 7.45-7.41 (m, 1H), 7.28-7.26 (m, 1H).
I (lit.)[6] : ; ^{1}H NMR (500 MHz, $CDCl_3$) δ **8.18 (dd, J = 1.6, 0.6 Hz, 1H)**, 7.66 – 7.56 (m, 2H), 7.43 (dd, J = 5.5, 0.5 Hz, 1H), 7.25 (dd, J = 5.4, 0.7 Hz, 1H).

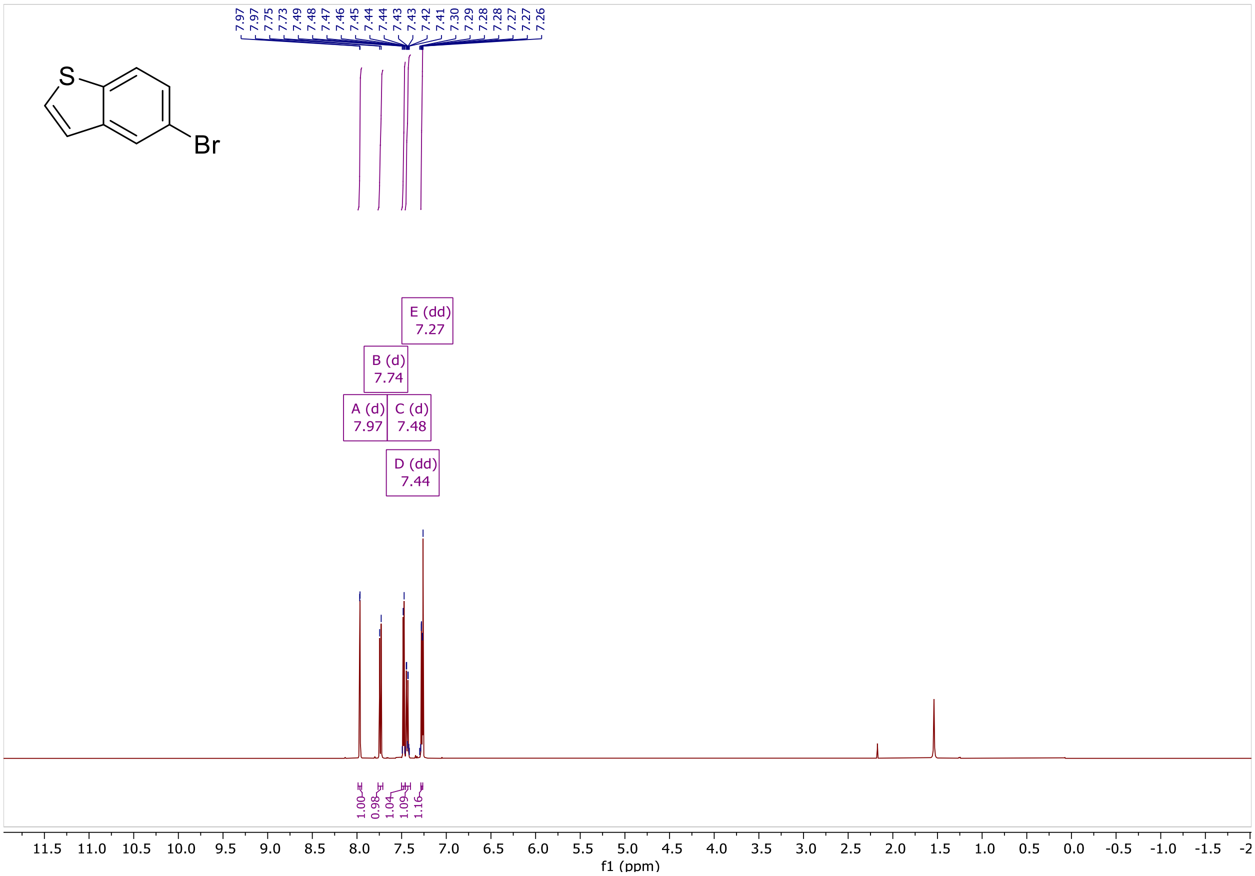

Figure S23. ^{1}H NMR spectrum of **5-bromo-benzo[b]thiophene** in $CDCl_3$

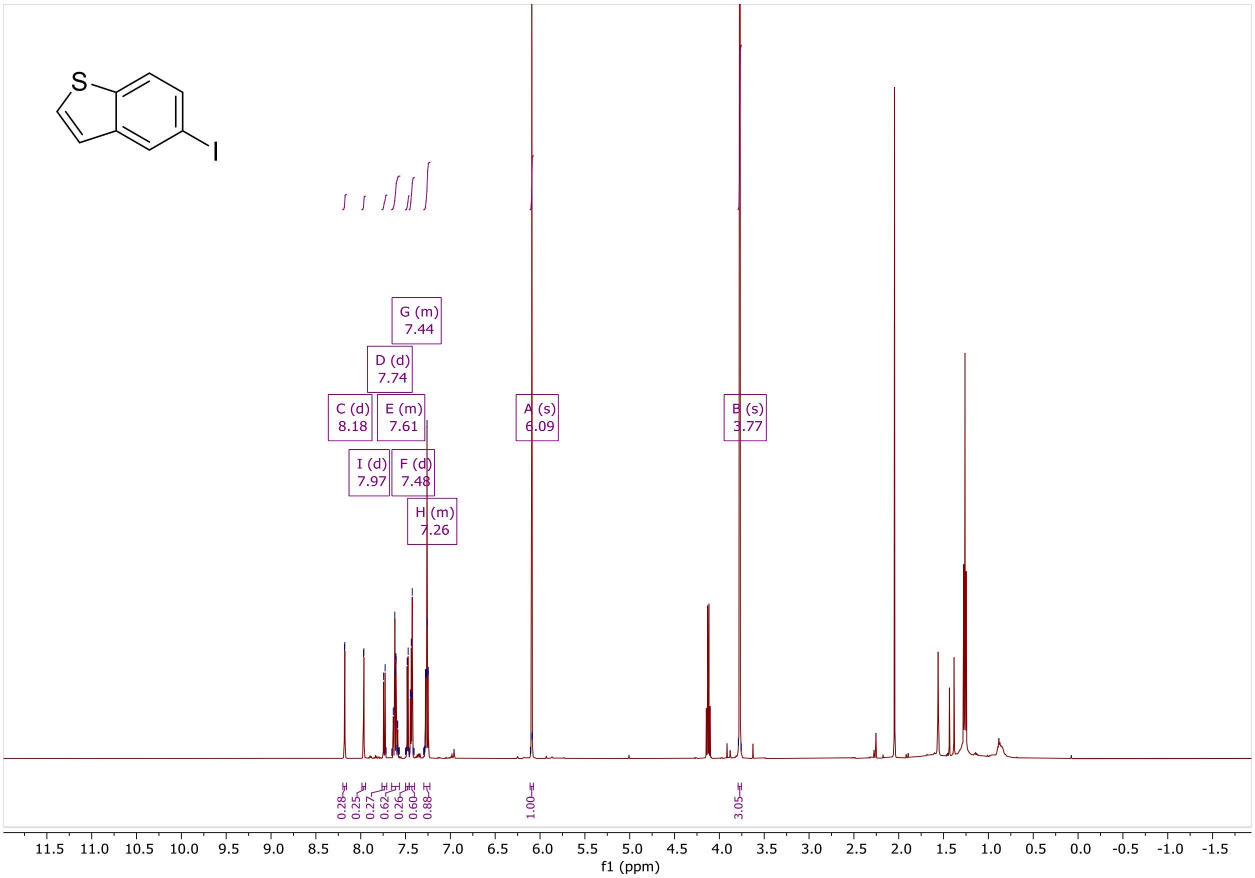

Figure S24. ^{1}H NMR spectrum of **16** in $CDCl_3$

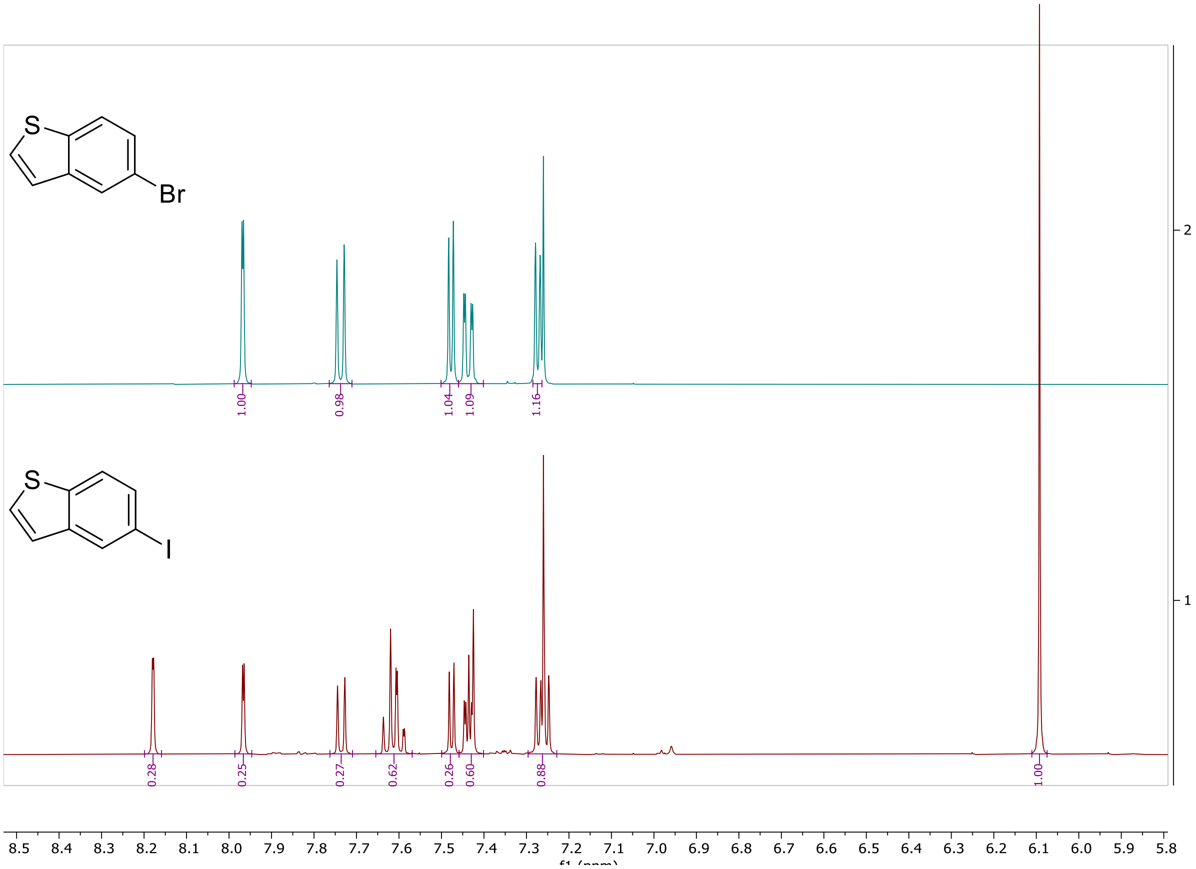

Figure S25. Overlaid ^{1}H NMR spectrum of **5-bromobenzo[b]thiophene** and **16** in $CDCl_3$

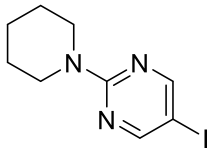

5-Iodo-2-(piperidin-1-yl)pyrimidine (17)

Following the General Procedure 1, 5-bromo-2-(piperidin-1-yl)pyrimidine (121.0 mg, 0.5 mmol) was converted into 5-iodo-2-(piperidin-1-yl)pyrimidine. Filtration provided the mixture of starting material and **17** (Br: 28%, I: 72%) according to q^{1}H. ^{1}H NMR (400 MHz, $CDCl_3$) δ **8.35 (s, 1.43H)**, **8.25 (s, 0.57H)**, <u>6.09 (s, 1H)</u>, <u>3.77 (s, 3H)</u>, 3.76 – 3.71 (m, 5H), 1.72 – 1.63 (m, 2H), 1.59 (ddd, *J* = 12.9, 6.6, 4.3 Hz, 7H).

Br (lit.)[7] : ^{1}H NMR (600 MHz, $CDCl_3$) δ: **8.24 (s, 2H)**, 3.75 – 3.70 (m, 4H), 1.65 (m, J = 6.5 Hz, 2H), 1.58 (m, J = 5.5 Hz, 4H)

I (lit.)[8] : ^{1}H NMR (500 MHz, $CDCl_3$) δ **8.34 (s, 2H)**, 3.78 – 3.69 (m, 4H), 1.71 – 1.63 (m, 2H), 1.59 (tt, J = 7.8, 4.5 Hz, 4H)

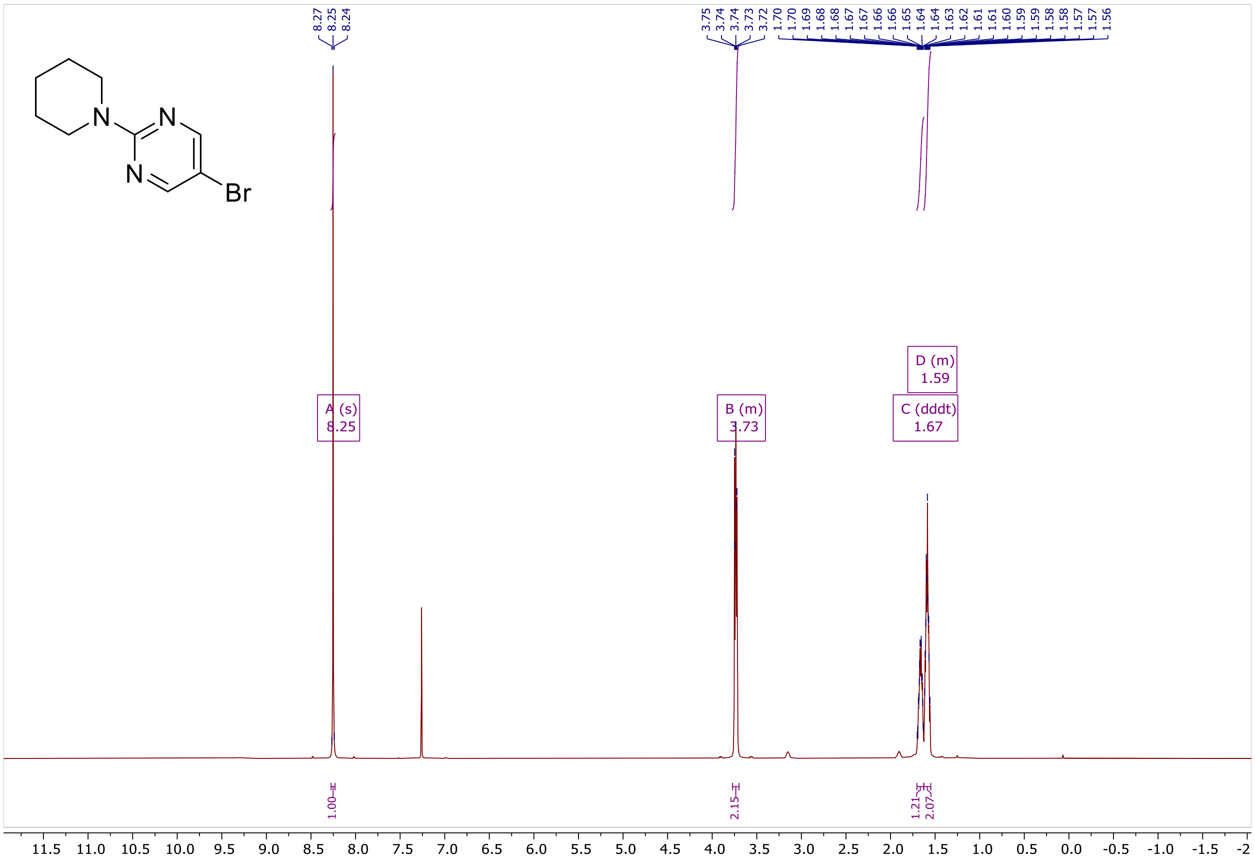

Figure S26. ^{1}H NMR spectrum of **5-bromo-2-(piperidin-1-yl)pyrimidine** in $CDCl_3$

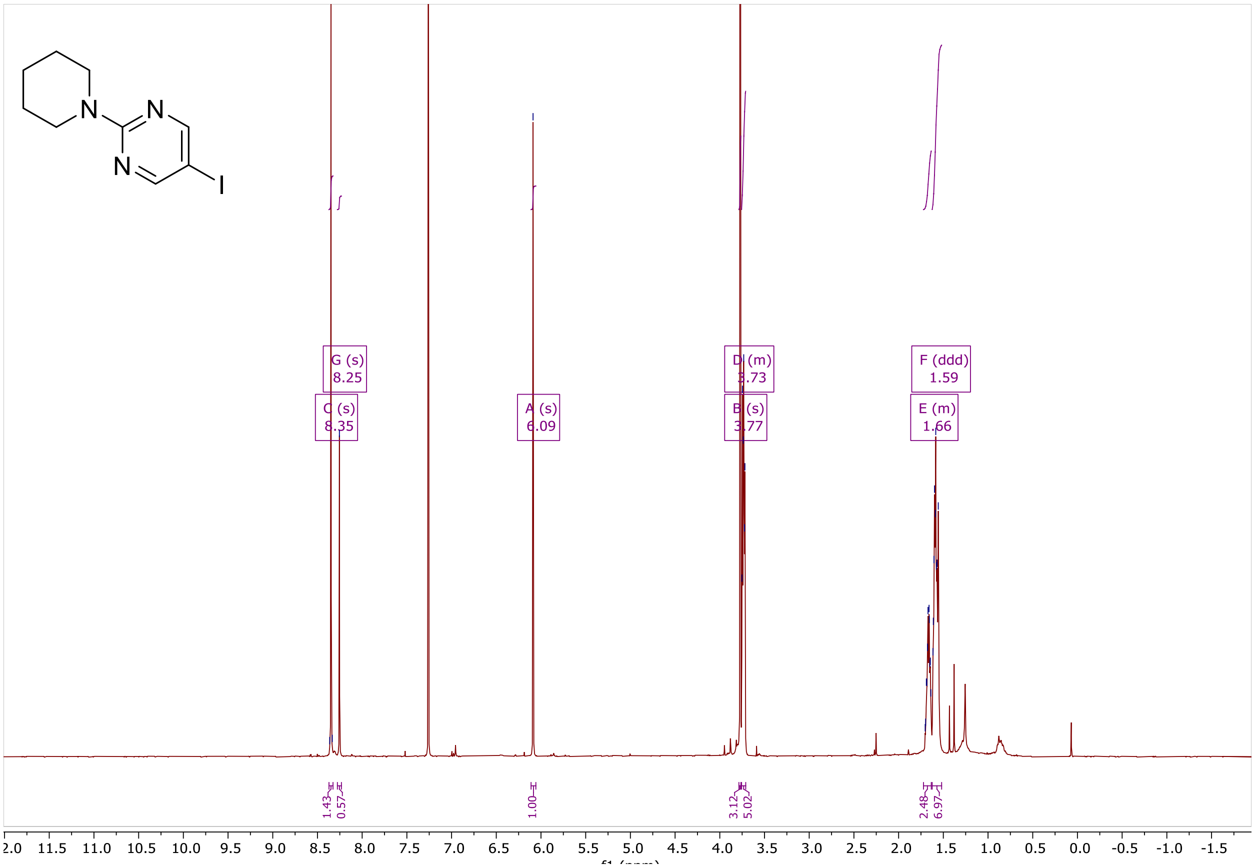

Figure S27. ^{1}H NMR spectrum of **17** in $CDCl_3$

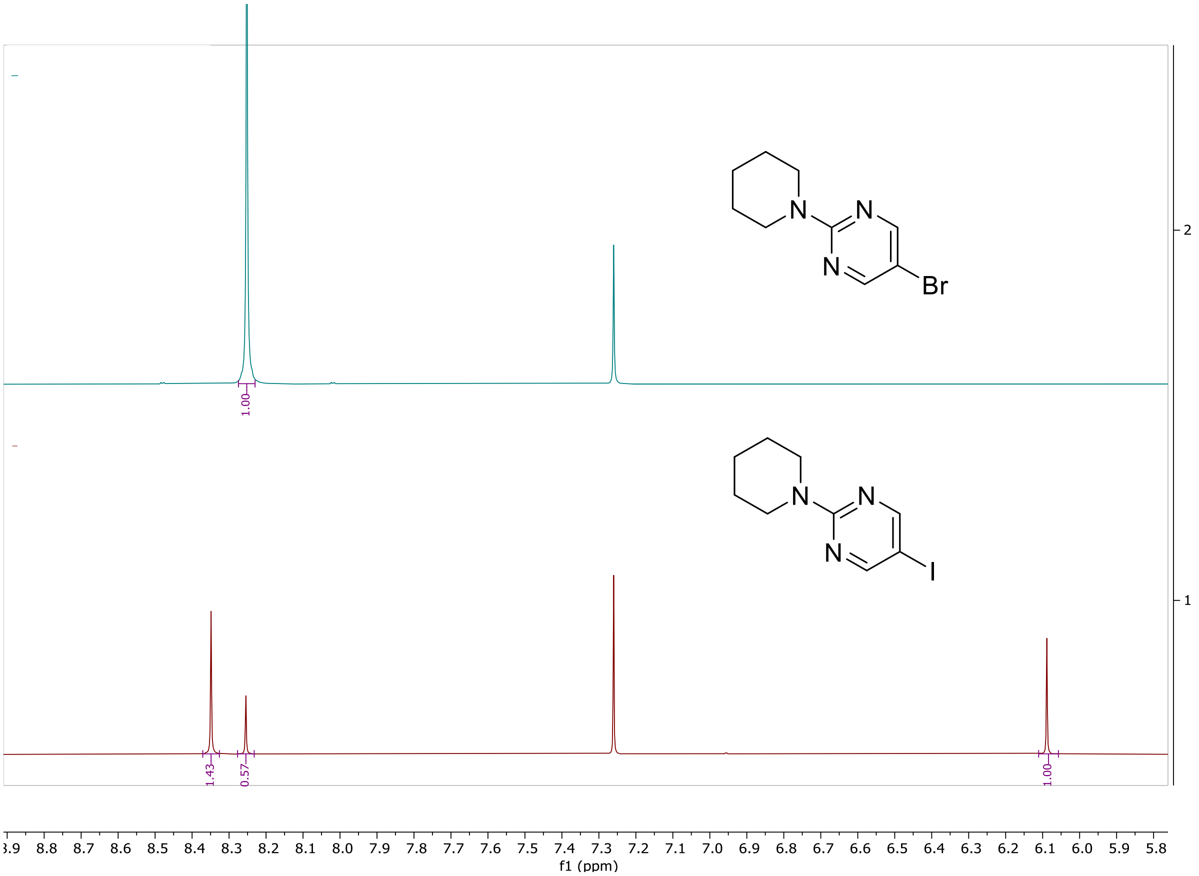

Figure S28. Overlaid ^{1}H NMR spectrum of **5-bromo-2-(piperidin-1-yl)pyrimidine** and **17** in $CDCl_3$

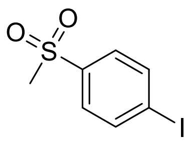

4-Iodophenyl methyl sulfone (18)

Following the General Procedure 1, 4-bromophenyl methyl sulfone (117.5 mg, 0.5 mmol) was converted into 4-iodophenyl methyl sulfone. Filtration provided the mixture of starting material and **18** (Br: 15%, I: 84%) according to q^{1}H NMR. ^{1}H NMR (400 MHz, $CDCl_3$) δ **7.99 – 7.90 (m, 1.77H)**, **7.83 – 7.80 (m, 0.27H)**, 7.76 – 7.69 (m, 0.29H), 7.69 – 7.62 (m, 1.74H), <u>6.09 (s, 1H)</u>, <u>3.77 (s, 3H)</u>, 3.05 (d, *J* = 2.8 Hz, 3H).

Br (lit.)[9] : ^{1}H NMR (400 MHz, $CDCl_3$) δ **7.78-7.76 (m, 2H),** 7.69-7.67 (m, 2H), 3.02 (s, 3H).

I (lit.)[10] : ; ^{1}H NMR (400 MHz, $CDCl_3$) δ **7.93 (dd, J = 8.7, 1.9 Hz, 2H)**, 7.79 – 7.60 (m, 2H), 3.04 (s, 3H).

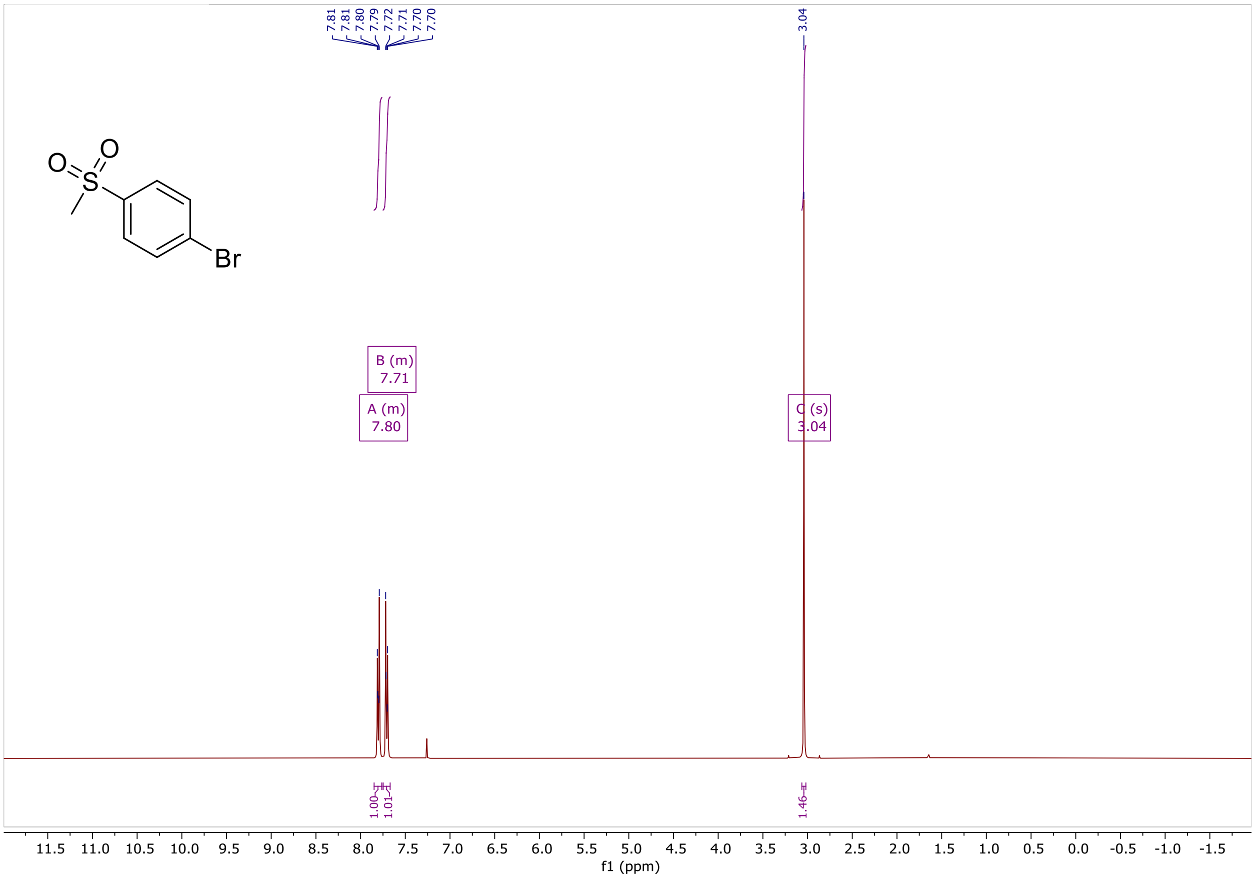

Figure S29. ^{1}H NMR spectrum of **4-bromophenyl methyl sulfone** in $CDCl_3$

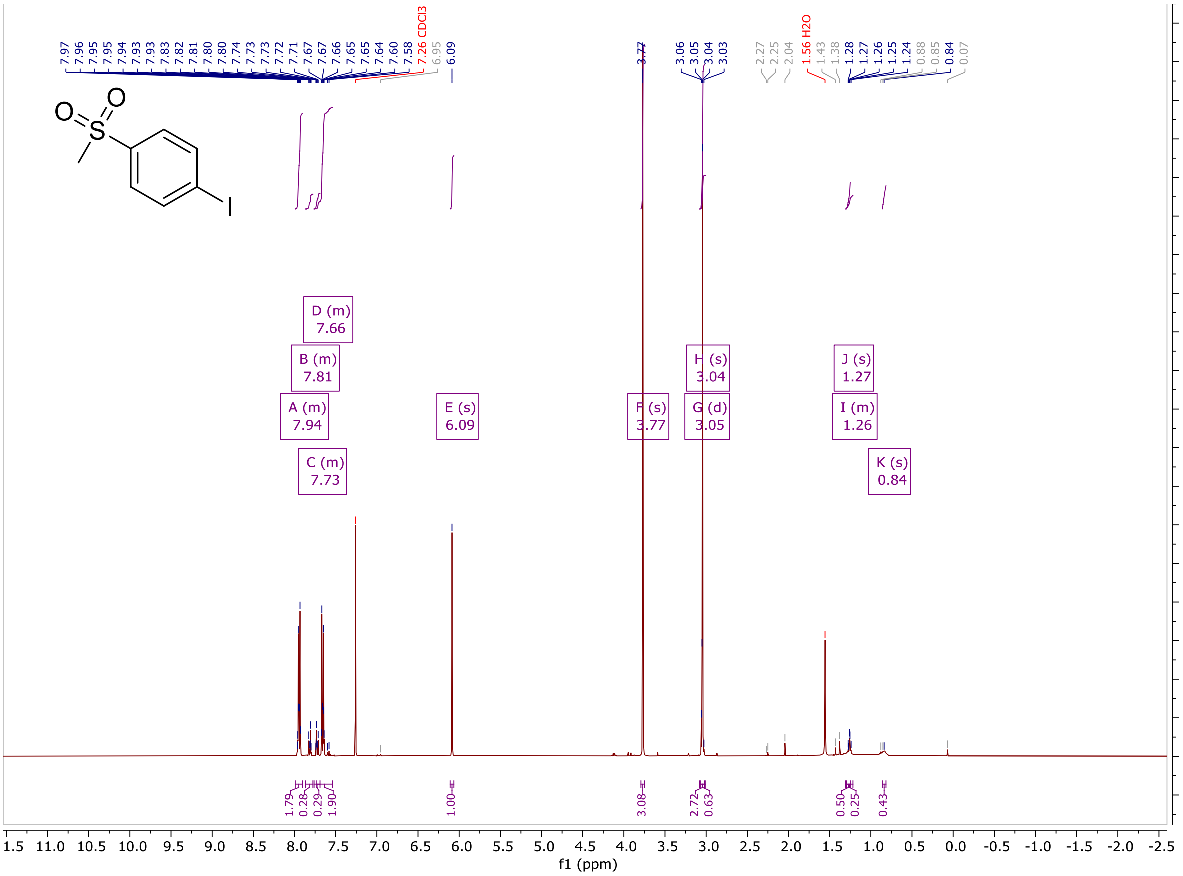

Figure S30. ^{1}H NMR spectrum of **18** in $CDCl_3$

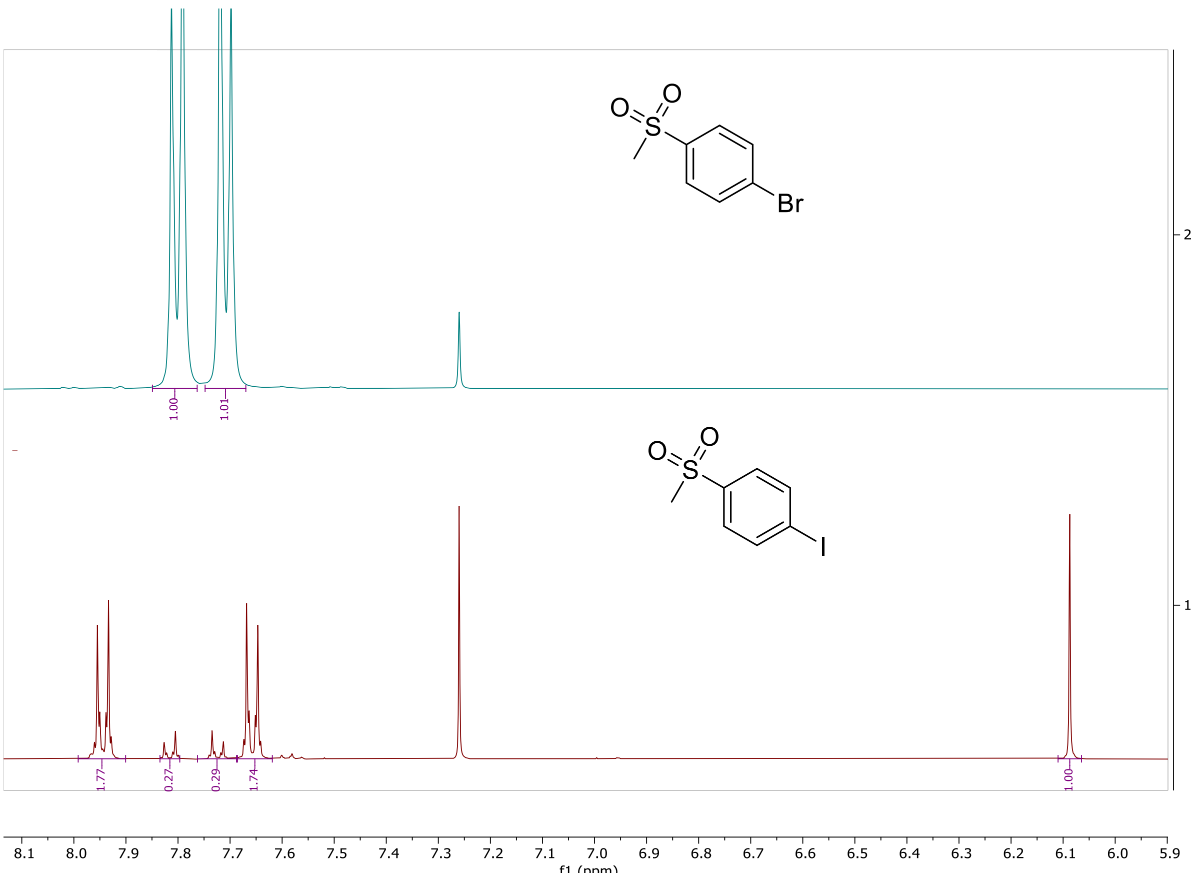

Figure S31. Overlaid ^{1}H NMR spectrum of **4-bromophenyl methyl sulfone** and **18** in $CDCl_3$

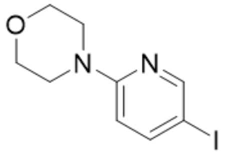

4-(5-Iodopyridin-2-yl)morpholine (19)

Following the General Procedure 1, 4-(5-bromopyridin-2-yl)morpholine (121.5 mg, 0.5 mmol) was converted into 4-(5-iodopyridin-2-yl)morpholine. This provided a mixture of starting material and **19** (Br: 22 %, I: 78 %) according to q^{1}H NMR. ^{1}H NMR (400 MHz, $CDCl_3$) δ 8.33 (d, *J* = 2.5 Hz, 0.7H), 8.20 (d, *J* = 2.7 Hz, 0.27H), 7.68 (dd, *J* = 8.9, 2.4 Hz, 0.85H), 7.55 (dd, *J* = 9.0, 2.5 Hz, 0.23H), **6.53 (d, *J* = 9.0 Hz, 0.22H)**, **6.47 (d, *J* = 8.9 Hz, 0.81H)**, 6.09 (s, 1H), 3.86 – 3.75 (m, 8H), 3.53 – 3.43 (m, 5H), 1.26 (q, *J* = 7.5 Hz, 1H), 0.85 (s, 1H).

Br (lit.)[11] : ^{1}H NMR (400 MHz, $CDCl_3$) δ 8.20 (d, J = 2.5 Hz, 1H), 7.55 (dd, *J* = 9.1, 2.5 Hz, 1H), **6.53 (d, *J* = 9.1 Hz, 1H)**, 3.81 (dd, *J* = 4.8, 4.8 Hz, 4H), 3.47 (dd, *J* = 4.8, 4.8 Hz, 4H).

I (lit.)[12] : ^{1}H NMR (400 MHz, $CDCl_3$) δ 8.32 (d, *J* = 2.3 Hz, 1H), 7.68 (dd, *J* = 8.9, 2.3 Hz, 1H), **6.47 (d, *J* = 8.9 Hz, 1H)**, 3.81 - 3.78 (m, 4H), 3.47 - 3.45 (m, 4H).

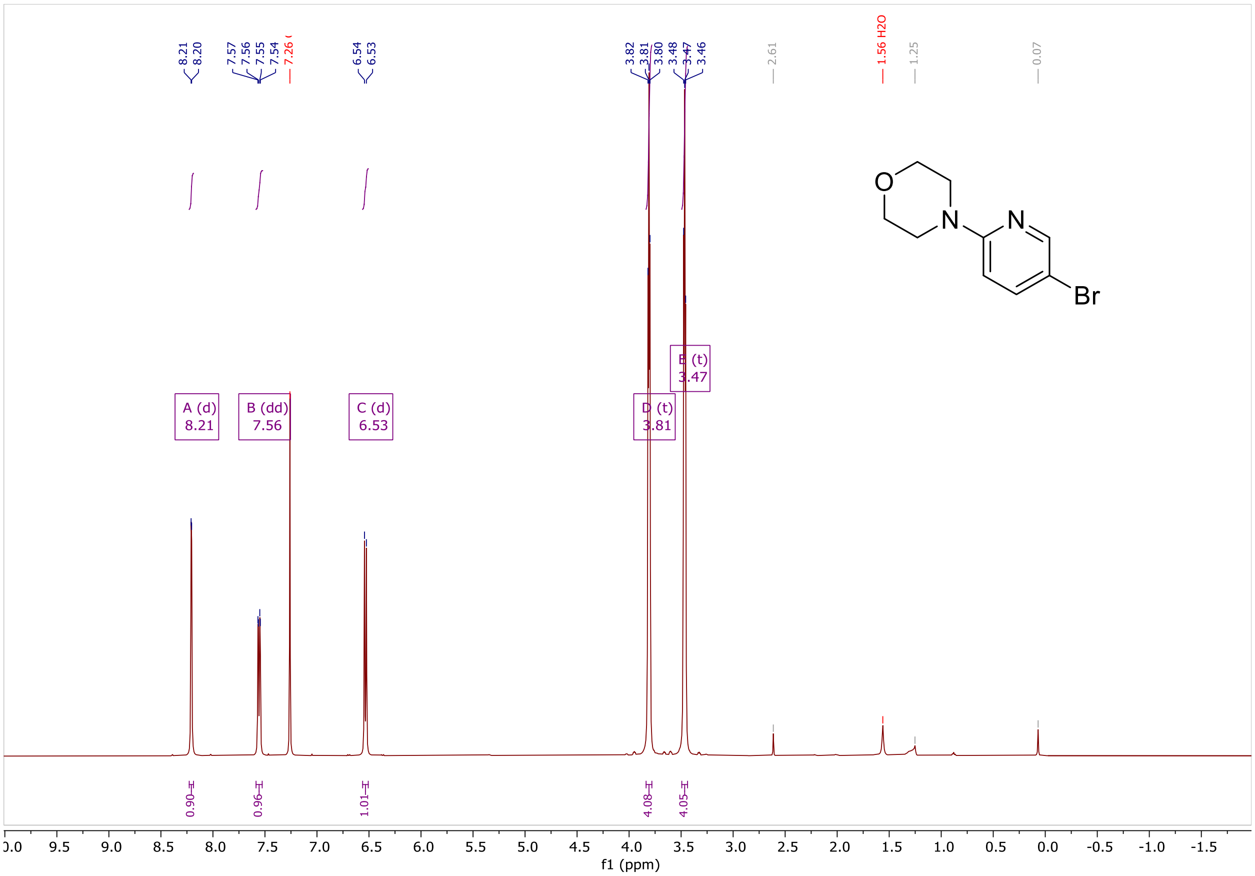

Figure S32. ^{1}H NMR spectrum of **5-bromo-2-morpholinopyridine** in $CDCl_3$

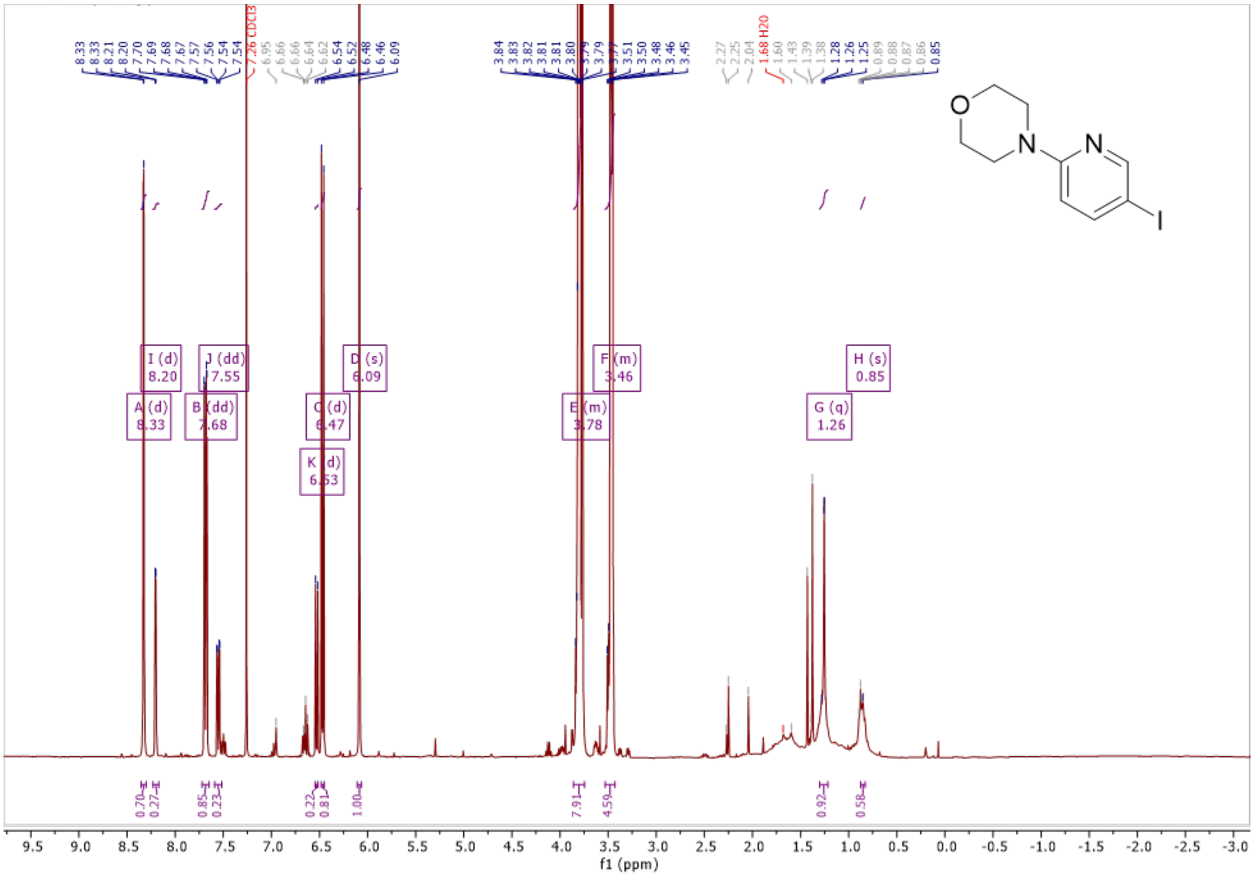

Figure S33. ^{1}H NMR spectrum of **19** in $CDCl_3$

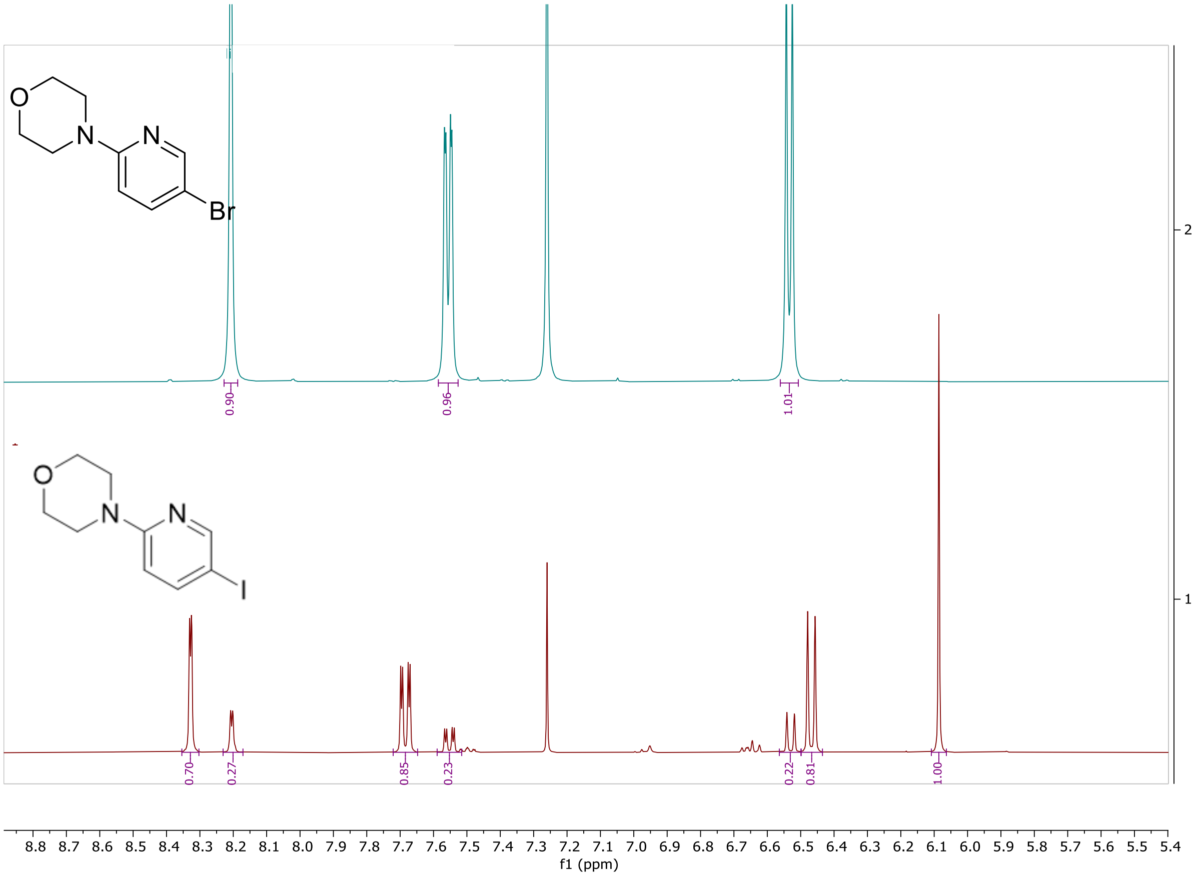

Figure S34. Overlaid ^{1}H NMR spectrum of **5-bromo-2-morpholinopyridine** and **19** in $CDCl_3$

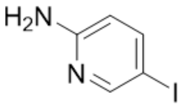

5-Iodopyridin-2-amine (20)

Following the General Procedure 3, 5-bromopyridin-2-amine (86.5 mg, 0.5 mmol) was converted into 5-iodopyridin-2-amine. This provided the mixture of starting material and **20** (1st pass: Br: 22%, I: 78%; 2nd pass: Br: 8%, I: 92%) according to q^{1}H NMR.
1st pass: ^{1}H NMR (400 MHz, $CDCl_3$) δ 8.23 (dd, *J* = 2.3, 0.7 Hz, 0.81H), 8.10 (dd, *J* = 2.4, 0.7 Hz, 0.20H), **7.63 (dd, *J* = 8.6, 2.3 Hz, 0.78H)**, **7.49 (dd, *J* = 8.7, 2.5 Hz, 0.22H)**, 6.41 (dd, *J* = 8.7, 0.7 Hz, 0.20H), 6.35 (dd, *J* = 8.6, 0.8 Hz, 0.81H), 6.09 (s, 1H), 4.46 (s, 2H), 3.77 (s, 3H), 1.30 – 1.22 (m, 0H), 0.85 (s, 0H).
2nd pass: ^{1}H NMR (500 MHz, $CDCl_3$) δ 8.23 (d, *J* = 2.2 Hz, 0.90H), 8.10 (d, *J* = 2.4 Hz, 0.08H), **7.63 (dd, *J* = 8.6, 2.2 Hz, 0.92H)**, **7.50 – 7.47 (m, 0.08H)**, 6.42 (d, *J* = 8.7 Hz, 0.08H), 6.36 (d, *J* = 8.6 Hz, 0.89H), 6.09 (s, 1H), 4.44 (s, 3H), 3.77 (s, 3H), 1.30 – 1.23 (m, 0H), 0.86 (s, 1H).

Br (lit.)[13] : ^{1}H NMR (400 MHz, $CDCl_3$): δ 8.08 (s, 1H), **7.48 (d, *J* = 8.7 Hz, 1H)**, 6.40 (d, *J* = 8.7 Hz, 1H), 4.52 (bs, 2H).
I (lit.)[14] : ^{1}H NMR (400 MHz, $CDCl_3$) δ 8.23 (d, *J* = 1.7 Hz, 1H), **7.63 (dd, *J* = 8.6, 2.2 Hz, 1H)**, 6.36 (d, *J* = 8.6 Hz, 1H), 4.50 (s, 2H).

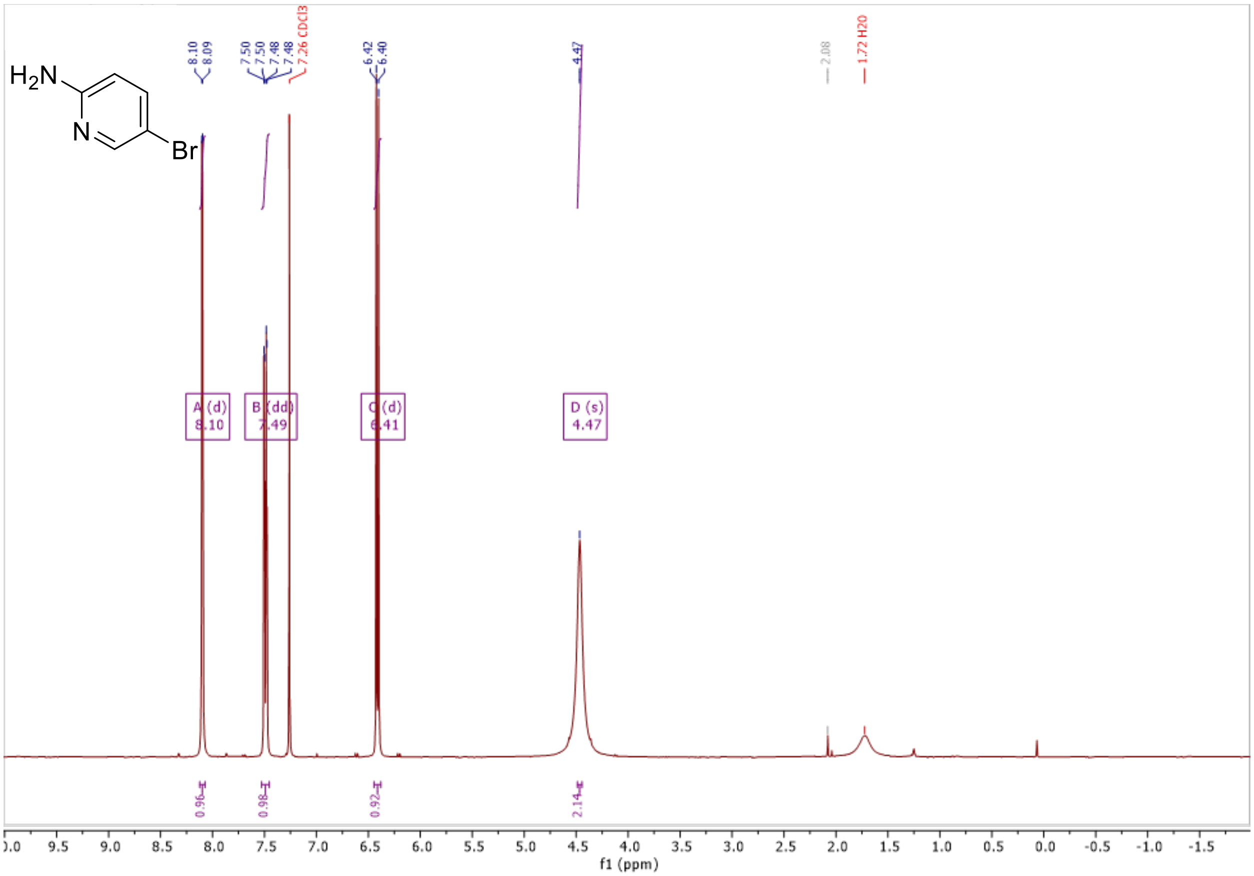

Figure S35. ^{1}H NMR spectrum of **5-bromopyridin-2-amine** in $CDCl_3$

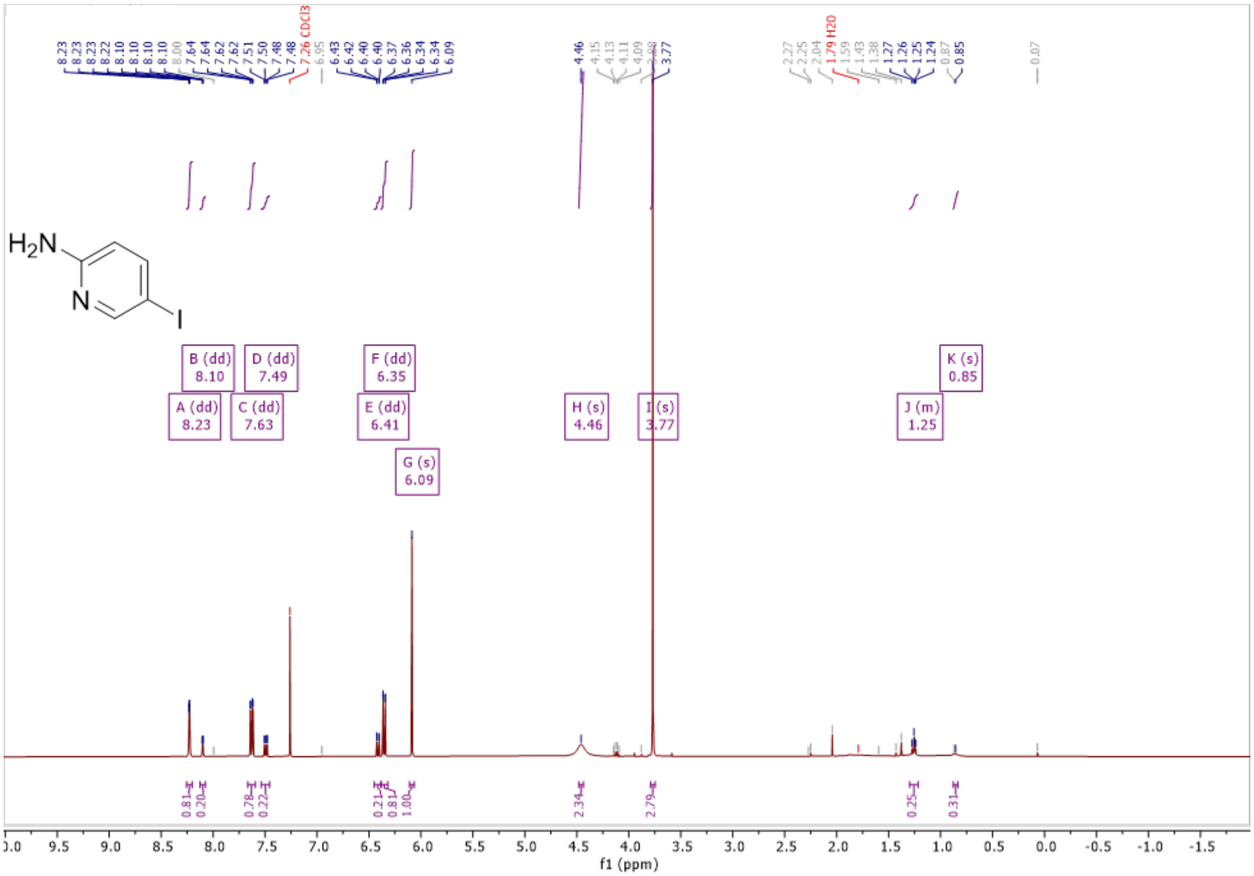

Figure S36. ^{1}H NMR spectrum of **20** (1st pass) in $CDCl_3$

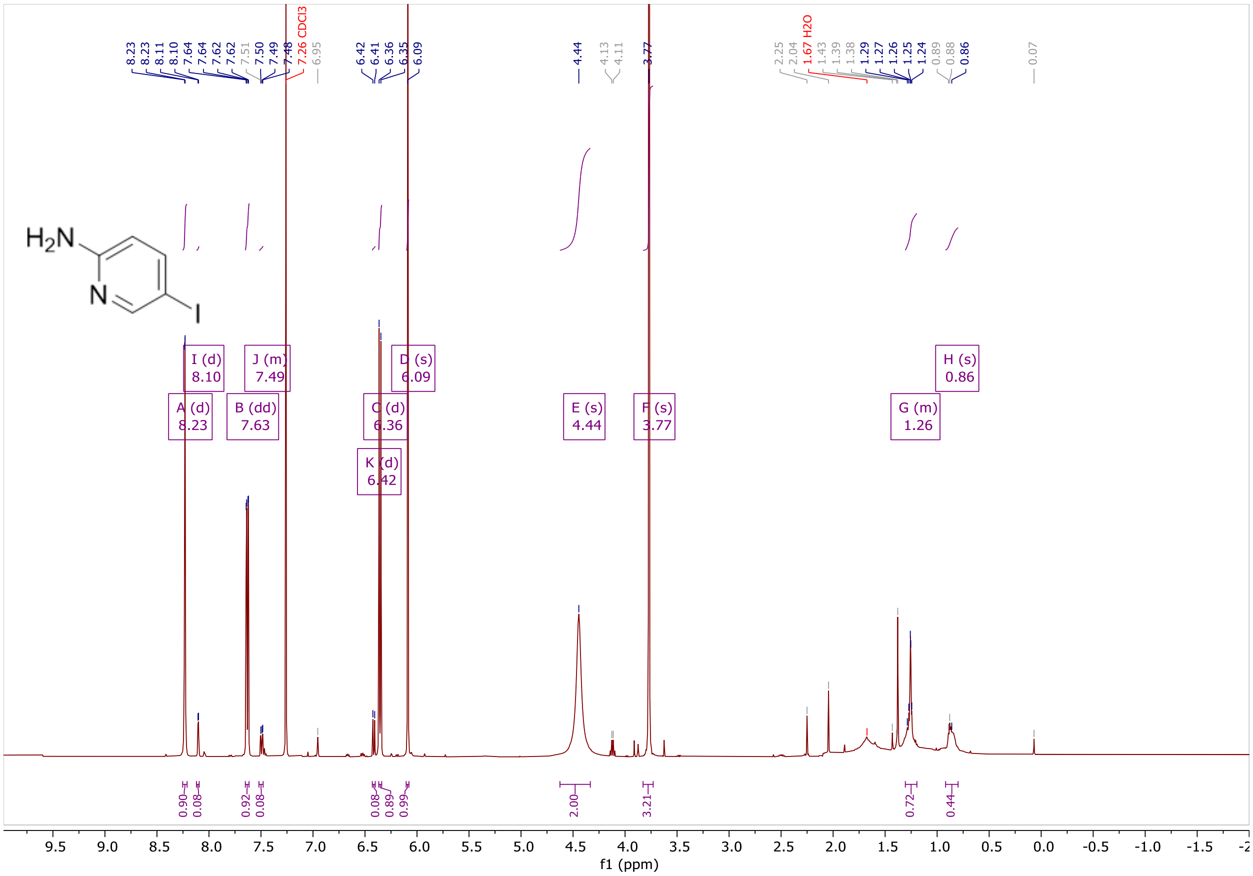

Figure S37. ^{1}H NMR spectrum of **20** (2nd pass) in $CDCl_3$

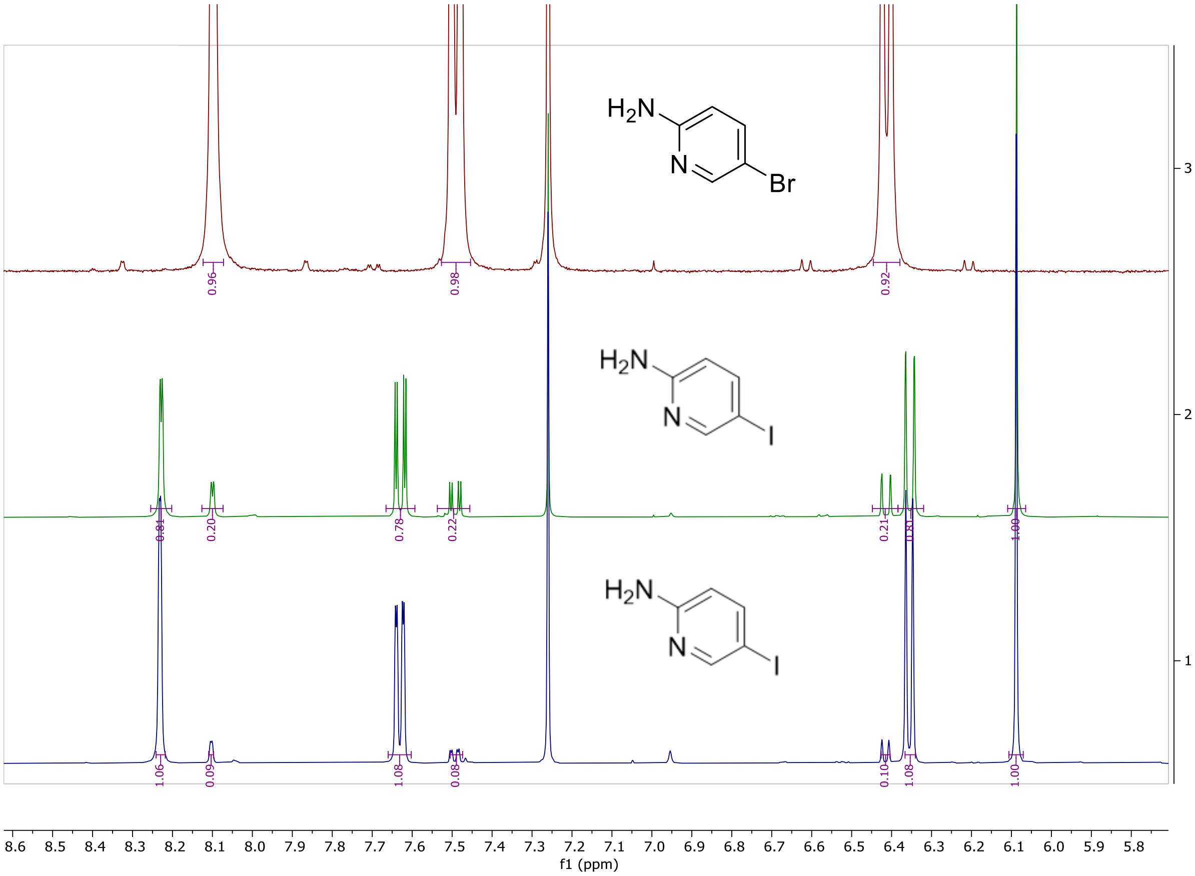

Figure S38. Overlaid ^{1}H NMR spectrum of **5-bromopyridin-2-amine** and **20** (1st and 2nd passes) in $CDCl_3$

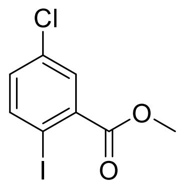

Methyl-5-chloro-2-iodobenzoate (21)

Following the General Procedure 3, methyl-5-chloro-2-bromobenzoate (124.8 mg, 0.5 mmol) was converted into methyl-5-chloro-2-iodobenzoate. This provided the mixture of starting material and **21** (1st pass: Br: 56%, I: 44%; 2nd pass: Br: 24%, I:76%) according to q^{1}H NMR. Note: For the first pass, 21.0 mg (0.125 mmol) of internal standard was added, leading to a reference integration of 0.75 at 6.07 ppm.

1st pass: ^{1}H NMR (400 MHz, $CDCl_3$) δ 7.91 (d, *J* = 8.4 Hz, 0H), 7.78 (q, *J* = 4.4 Hz, 1H), 7.59 (d, *J* = 8.6 Hz, 1H), **7.30 (dd, *J* = 8.5, 2.6 Hz, 0.57 H)**, **7.14 (dd, *J* = 8.5, 2.6 Hz, 0.44 H)**, 6.09 (s, 0.75 H), 3.94 (s, 3H), 3.77 (s, 2.30 H), 2.05 (s, 0H), 1.45 – 1.36 (m, 0H), 1.31 (s, 0H), 1.25 (s, 0H), 0.86 (s, 0H).
2nd Pass: ^{1}H NMR (400 MHz, $CDCl_3$) δ 7.91 (d, *J* = 8.4 Hz, 1H), 7.82 – 7.74 (m, 1H), 7.59 (dd, *J* = 8.5, 3.7 Hz, 0H), **7.30 (dt, *J* = 8.6, 3.3 Hz, 0.29 H)**, **7.14 (dd, *J* = 8.5, 2.6 Hz, 0.76 H)**, 6.09 (s, 1H), 4.41 (q, *J* = 7.1 Hz, 0H), 3.94 (s, 3H), 3.77 (s, 3H), 1.46 – 1.36 (m, 1H), 1.27 (d, *J* = 12.8 Hz, 1H), 0.86 (s, 1H).

Br (lit.)[15] : ^{1}H NMR (300 MHz, $CDCl_3$): δ = 7.78 (distorted dd, *J* = 2.6, 0.8, 1 H), 7.58 (distorted dd, *J* = 8.5, 0.8, 1 H), **7.30 (distorted ddd, *J* = 8.5, 2.6, 0.8, 1 H)**, 3.94 (s, 3 H)
I (lit.)[16] : ^{1}H NMR (400 MHz, $CDCl_3$) δ 7.90 (dd, *J* = 8.4, 3.3 Hz, 1H), 7.79 (t, *J* = 2.7 Hz, 1H), **7.18 – 7.09 (m, 1H)**, 3.93 (s, 3H).

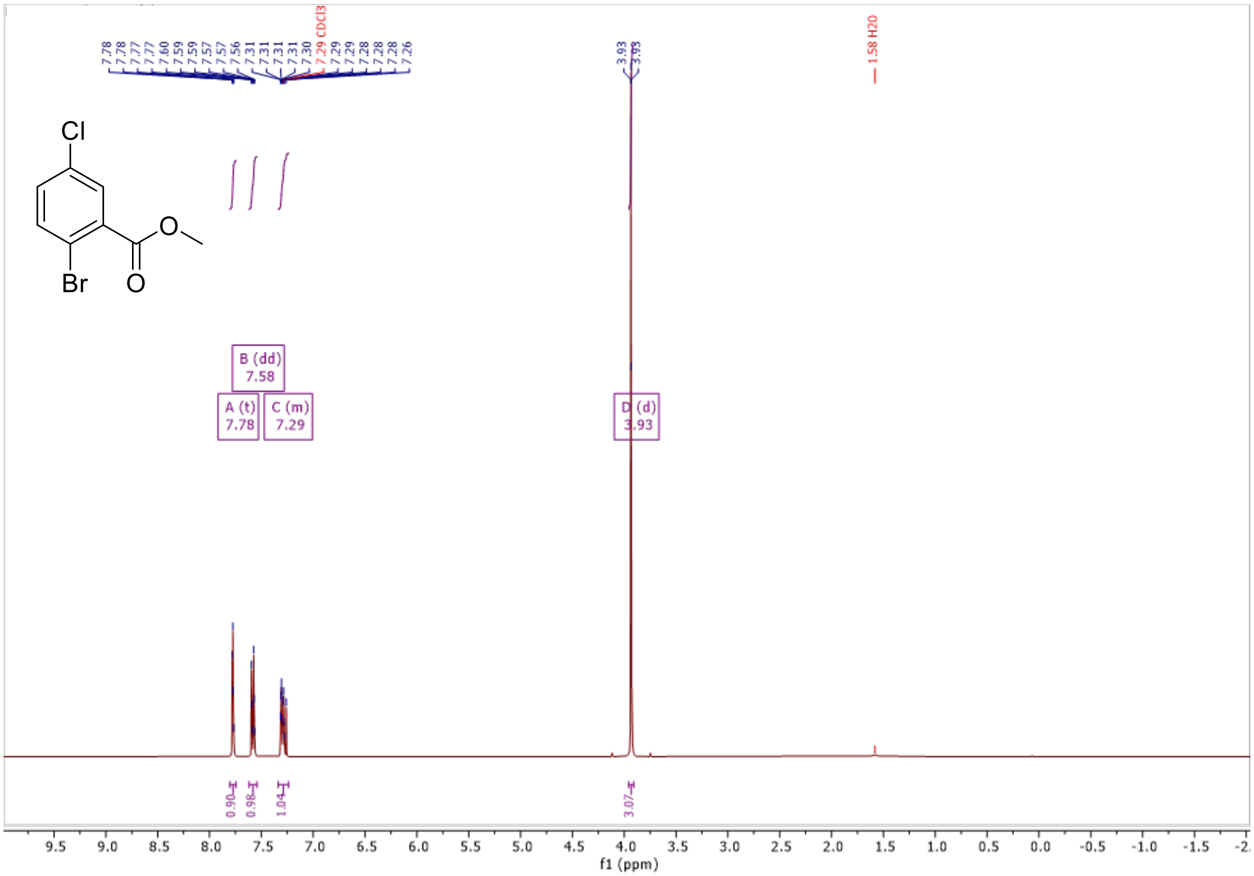

Figure S39. ^{1}H NMR spectrum of **methyl 4-chloro-2-bromobenzoate** in $CDCl_3$

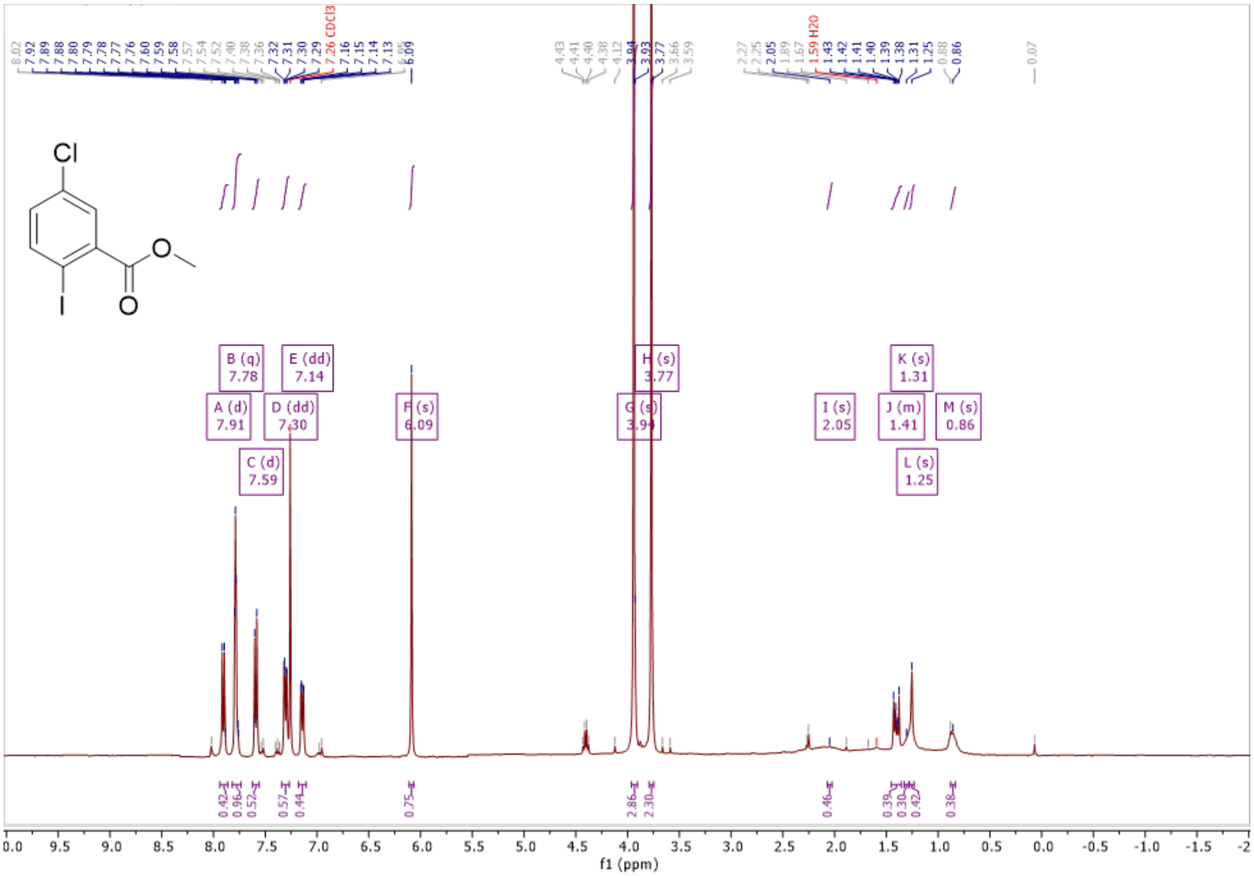

Figure S40. ^{1}H NMR spectrum of **21** (1st pass) in $CDCl_3$

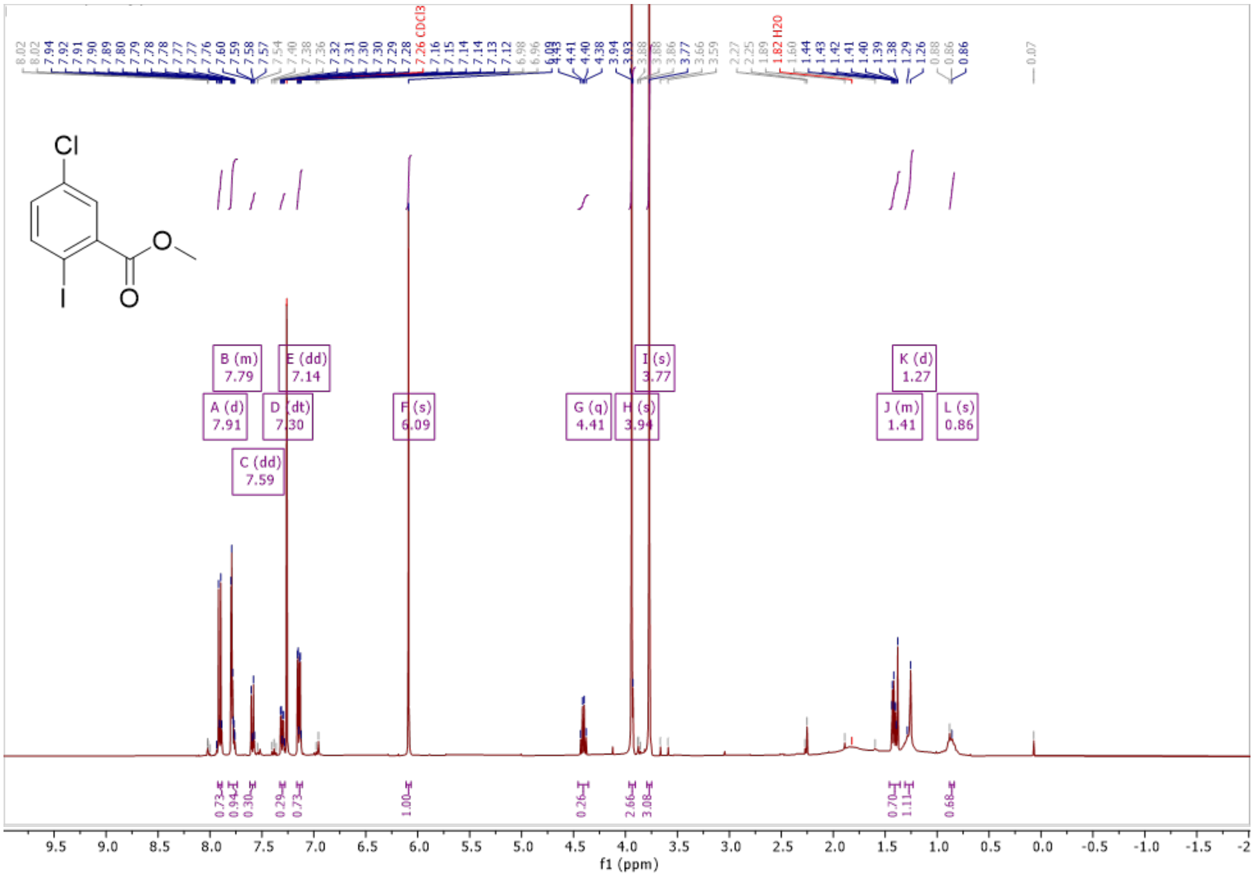

Figure S41. ^{1}H NMR spectrum of **21** (2nd pass) in $CDCl_3$

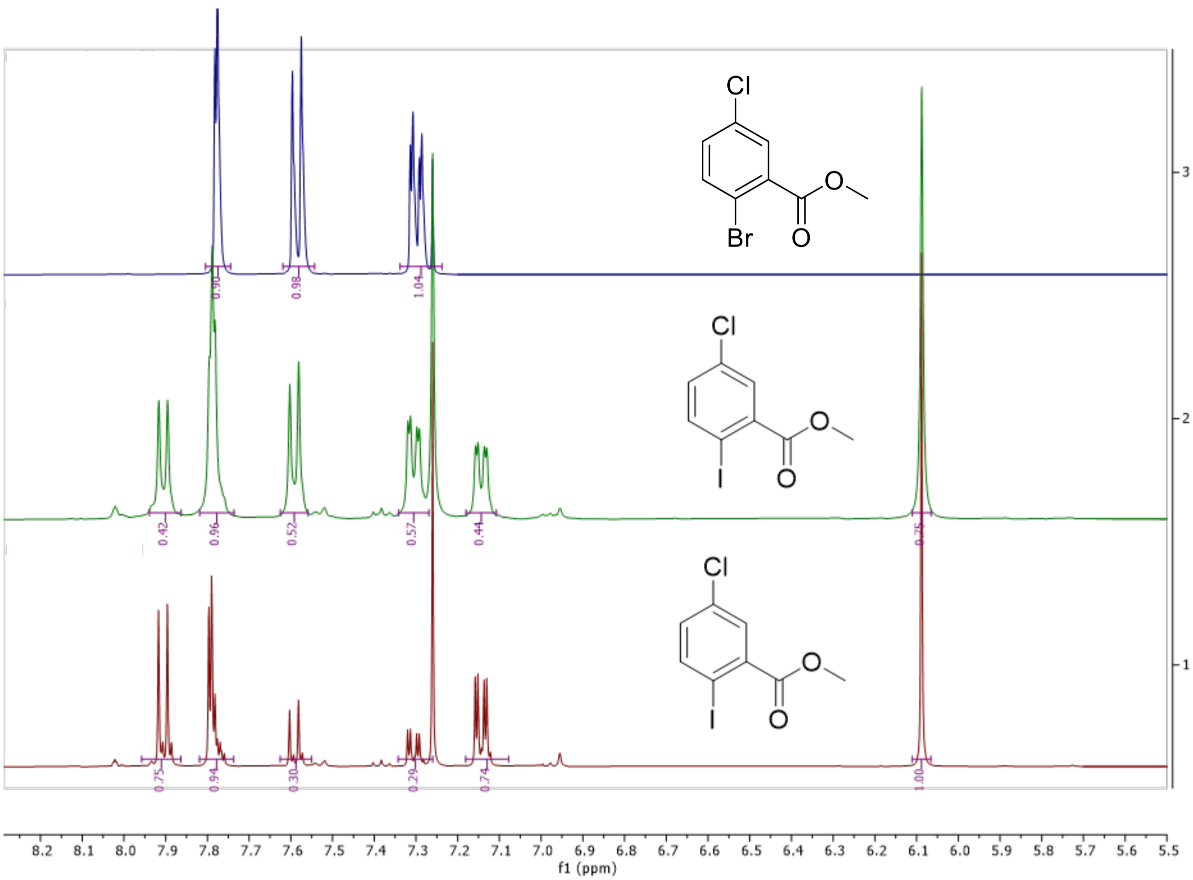

Figure S42. Overlaid ^{1}H NMR spectrum of **methyl 5-chloro-2-bromobenzoate** and **21** (1st and 2nd passes) in $CDCl_3$

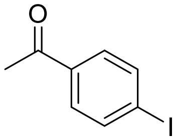

4-Iodoacetophenone (22)

Following the General Procedure 3, 4-bromoacetophenone (99.5 mg, 0.5 mmol) was converted into 4-iodoacetophenone. This provided the mixture of starting material and **22** (1st pass: Br: 21.5%, I: 76.5%; 2nd pass: Br: 10%, I: 90%) according to q^{1}H NMR. Note: 2nd pass data was obtained from a 0.25 mmol (49 mg) scale reaction.

1st pass: ^{1}H NMR (400 MHz, $CDCl_3$) δ 7.87 – 7.78 (m, 2H), **7.69 – 7.63 (m, 1.53 H)**, **7.62 – 7.58 (m, 0.43 H)**, 6.08 (s, 1H), 3.77 (s, 3H), 2.58 (d, *J* = 4.8 Hz, 3H).

2nd pass (0.25 mmol scale): ^{1}H NMR (500 MHz, $CDCl_3$) δ 7.86 – 7.78 (m, 1H), **7.67 – 7.64 (m, 0.91 H)**, **7.61 – 7.59 (m, 0.10 H)**, 6.08 (s, 1H), 3.77 (s, 3H), 2.57 (s, 2H).

Br (lit.)[17] : ^{1}H NMR (400 MHz, $CDCl_3$) δ 7.85-7.79 (m, 2H), **7.64-7.58 (m, 2H)**, 2.59 (s, 3H)
I (lit.)[18]: ^{1}H NMR (500 MHz, $CDCl_3$) δ 7.85 (2 H, d, *J* 8.0), **7.68 (2 H, d, *J* 8.1)**, 2.59 (3 H, s).

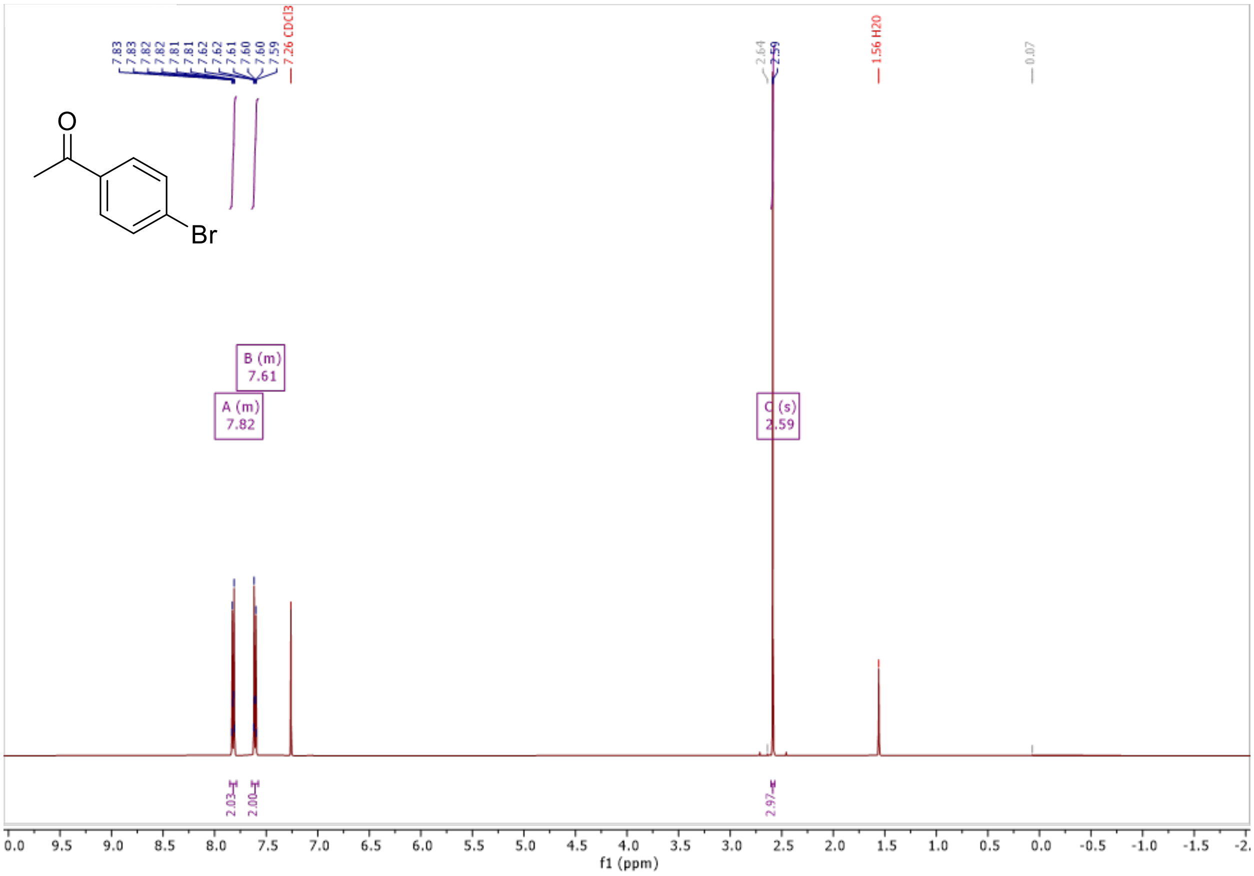

Figure S43. ^{1}H NMR spectrum of **4-bromoacetophenone** in $CDCl_3$

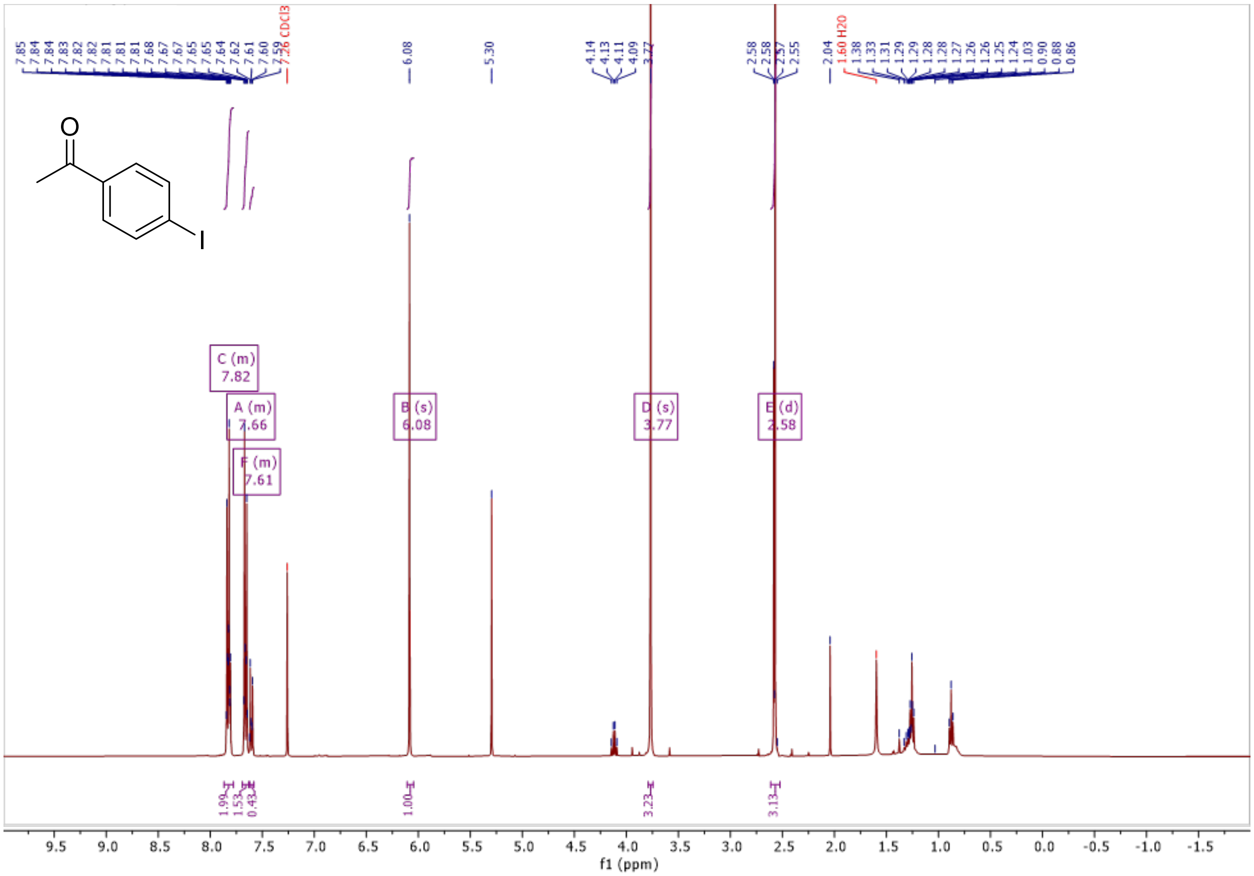

Figure S44. ^{1}H NMR spectrum of **22** (1st pass) in $CDCl_3$

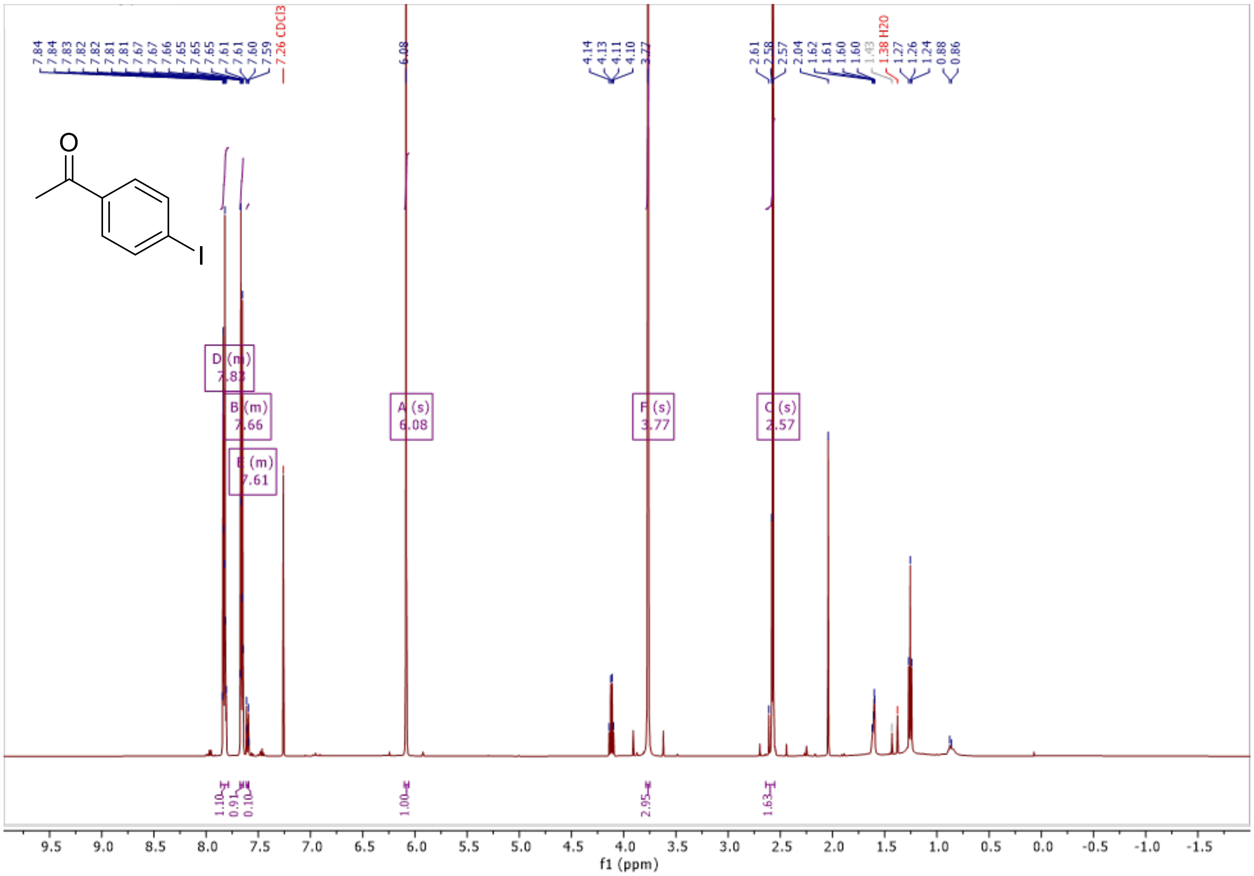

Figure S45. ^{1}H NMR spectrum of **22** (2nd pass) in $CDCl_3$

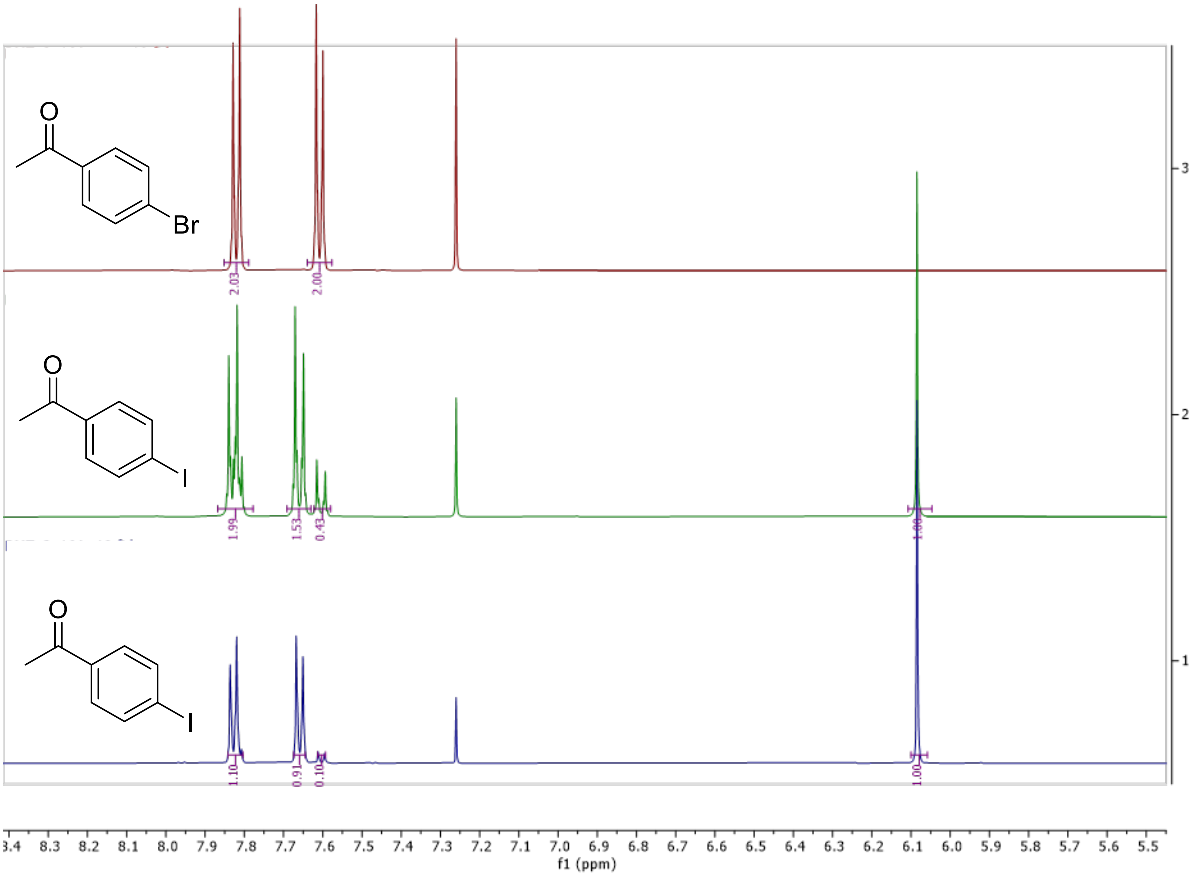

Figure S46. Overlaid ^{1}H NMR spectrum of **4-bromoacetophenone** and **22** (1st and 2nd passes) in $CDCl_3$

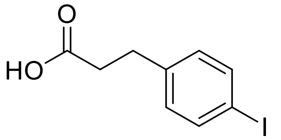

3-(4-Iodophenyl)propanoic acid 23)

Following the General Procedure 2, 3-(4-bromophenyl)propanoic acid (114.5 mg, 0.5 mmol) was converted into 3-(4-iodophenyl)propanoic acid. This provided the mixture of starting material and **23** (Br: 23%, I: 77%) according to q^{1}H NMR. ^{1}H NMR (400 MHz, $CDCl_3$) δ 7.61 – 7.58 (m, 1.73H), 7.42 – 7.37 (m, 0.45H), **7.08 (d, *J* = 8.3 Hz, 0.46H), 6.96 (d, *J* = 8.0 Hz, 1.64H),** 6.08 (s, 1H), 4.11 (q, *J* = 7.1 Hz, 3H), 3.76 (s, 3H), 2.89 (td, *J* = 7.7, 4.9 Hz, 2H), 2.63 (t, *J* = 7.6 Hz, 2H).

Br (lit.)[19] : ^{1}H NMR (400 MHz, $CDCl_3$) δ 11.45 (br s, 1H) 7.44-7.39 (m, 2H) **7.11-7.06 (m, 2H)**, 2.91 (t, 2H, *J* = 7.6 Hz) 2.66 (t, 2H, *J* = 7.6 Hz).

I (lit.)[20] : ^{1}H NMR (400 MHz, $CDCl_3$) δ 7.62 (d, *J* = 8.4 Hz, 2 H), **6.97 (d, *J* = 8.0 Hz, 2 H)**, 2.91 (t, *J* = 7.6 Hz, 2 H), 2.67 (t, *J* = 7.6 Hz, 2 H).

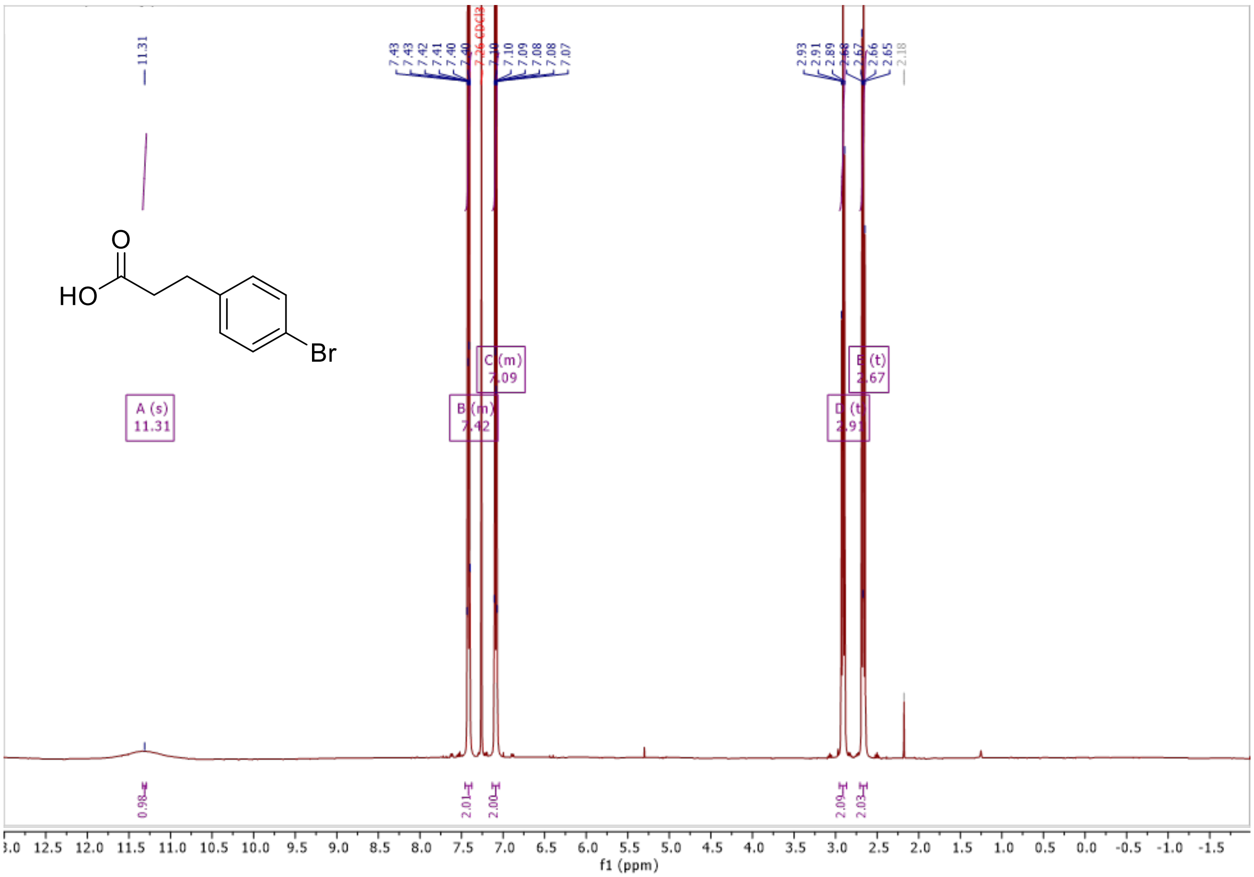

Figure S47. ^{1}H NMR spectrum of **3-(4-bromophenyl)propanoic acid** in $CDCl_3$

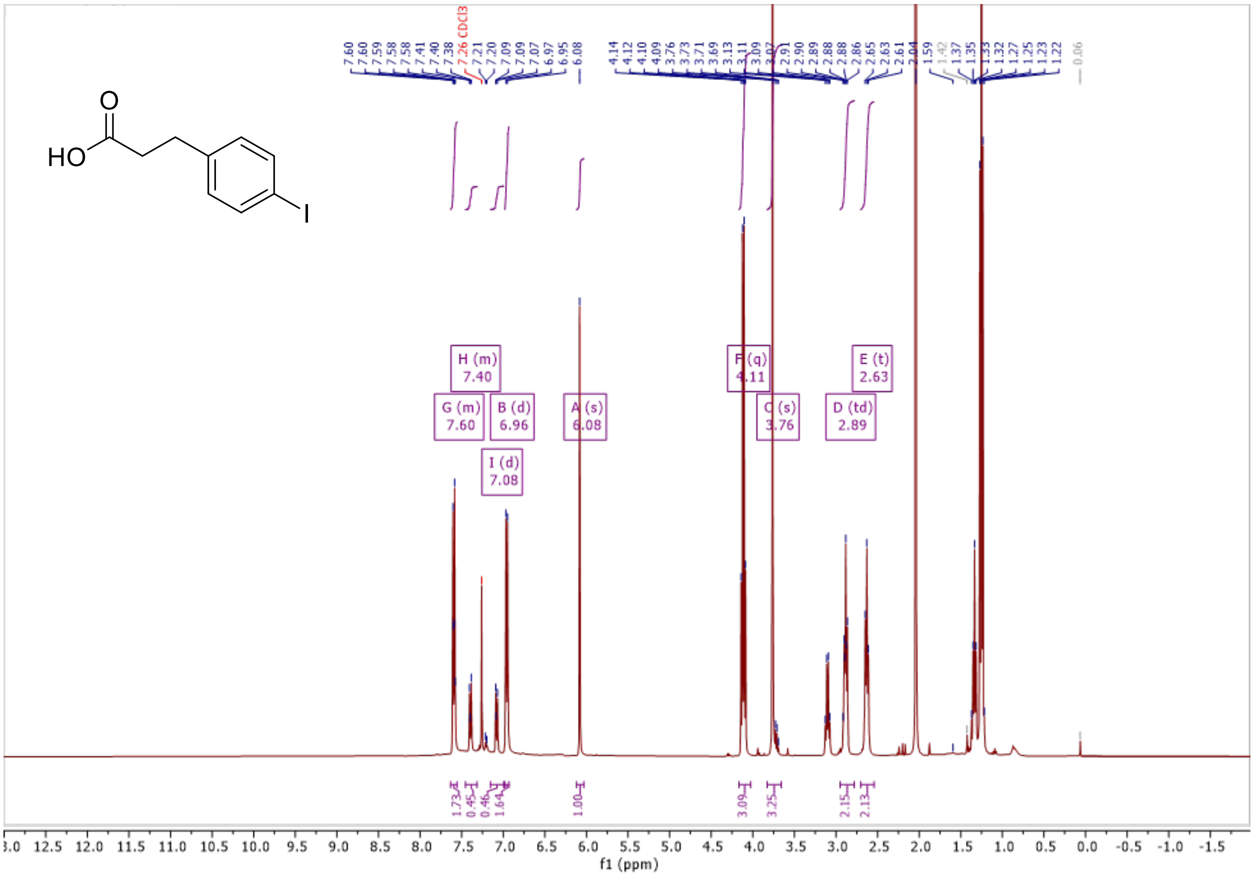

Figure S48. ^{1}H NMR spectrum of **23** in $CDCl_3$

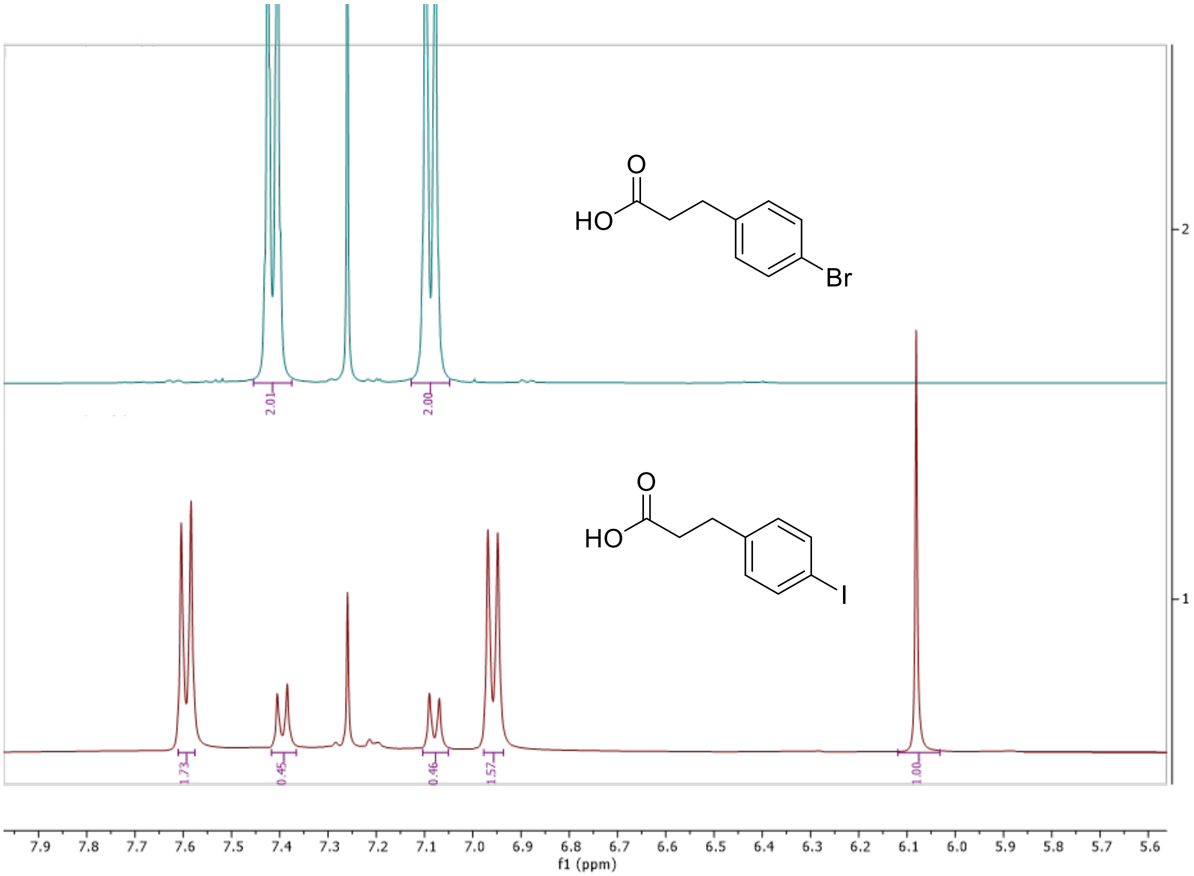

Figure S49. Overlaid ^{1}H NMR spectrum of **3-(4-bromophenyl)propanoic acid** and **23** in $CDCl_3$

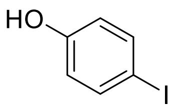

4-Iodophenol (24)

Following the General Procedure 2, 4-bromophenol (114.5 mg, 0.5 mmol) was converted into 4-iodophenol. This provided the mixture of starting material and **24** (Br: 32%, I: 56.5%) according to q^{1}H NMR. ^{1}H NMR (500 MHz, $CDCl_3$) δ **7.54 – 7.47 (m, 1.13H)**, **7.36 – 7.29 (m, 0.64H)**, 6.76 – 6.69 (m, 0.65H), 6.66 – 6.59 (m, 1.15H), 6.10 (d, *J* = 1.1 Hz, 1H), 5.28 (s, 1H), 4.14 (q, *J* = 7.2 Hz, 0H), 3.78 (s, 0H), 3.77 (s, 3H), 2.06 (s, 0H), 1.78 (s, 0H), 1.33 – 1.19 (m, 1H), 0.87 (dt, *J* = 14.1, 7.0 Hz, 0H).

Br (lit.)[21] : ^{1}H NMR (400 MHz, $CDCl_3$) δ = **7.28 (d, J = 8.9 Hz, 2 H)**, 6.72 (d, J = 8.9 Hz, 2 H).

I (lit.)[22] : ^{1}H NMR (400 MHz, $CDCl_3$) δ **7.52-7.502 (d, 2H)**, 6.64-6.61 (d, 2H), 5.34 (s, 1H).

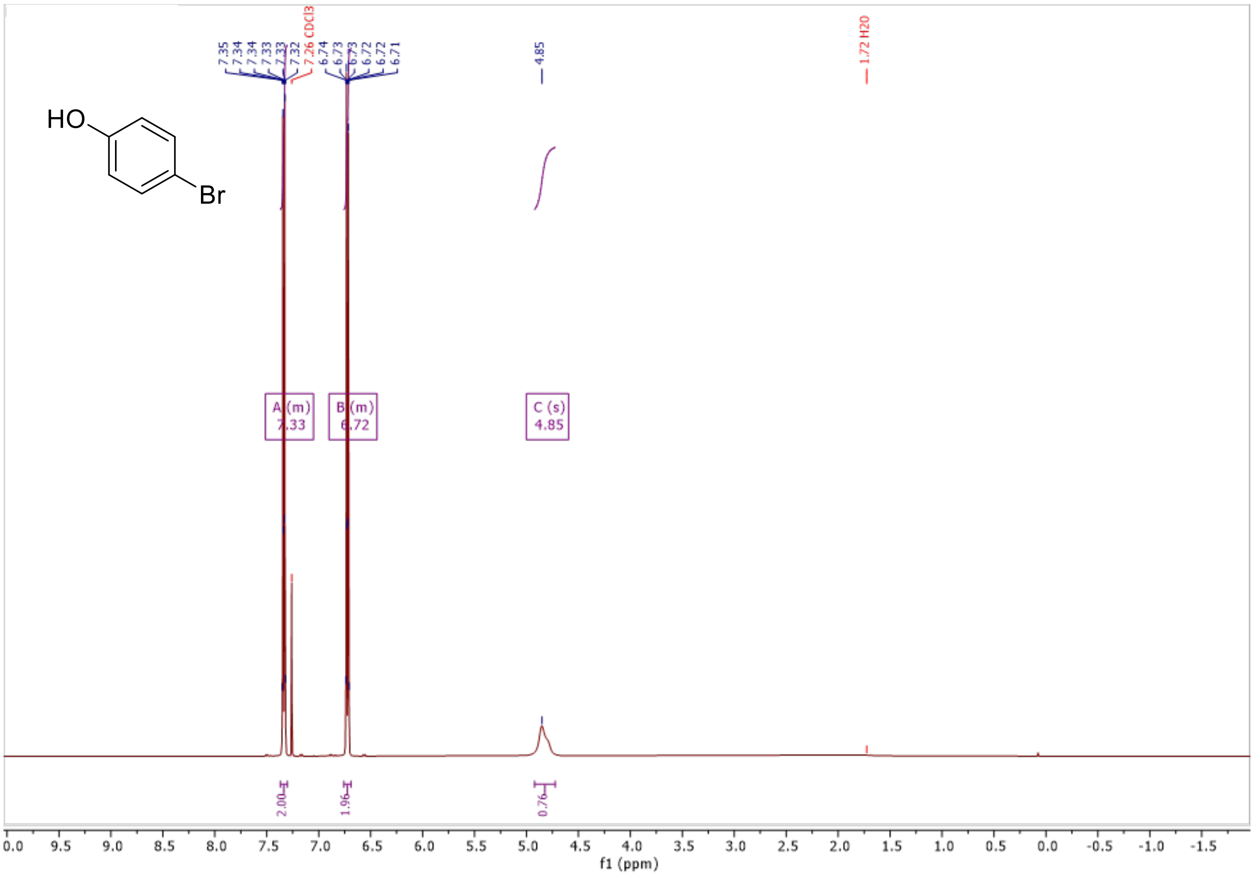

Figure S50. ^{1}H NMR spectrum of **4-bromophenol** in $CDCl_3$

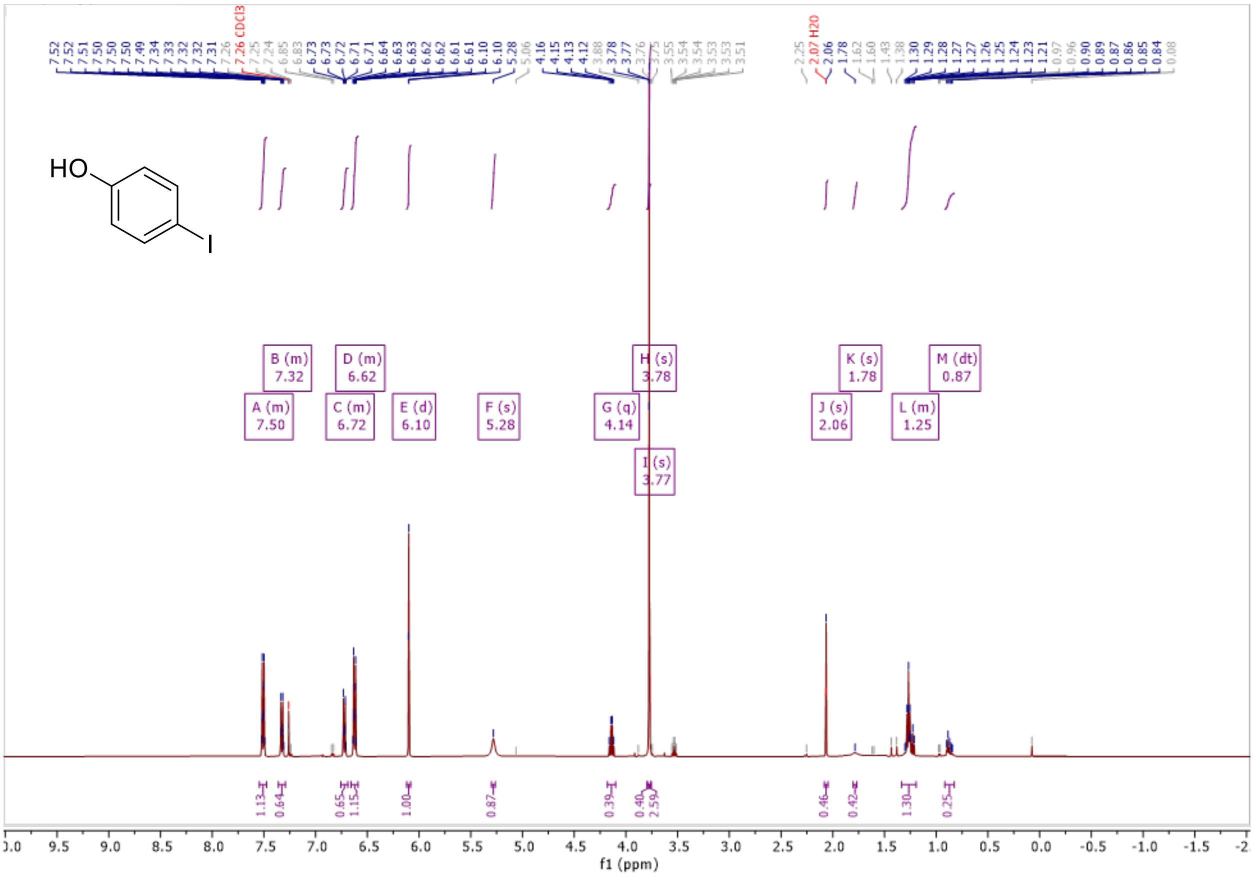

Figure S51. ^{1}H NMR spectrum of **24** in $CDCl_3$

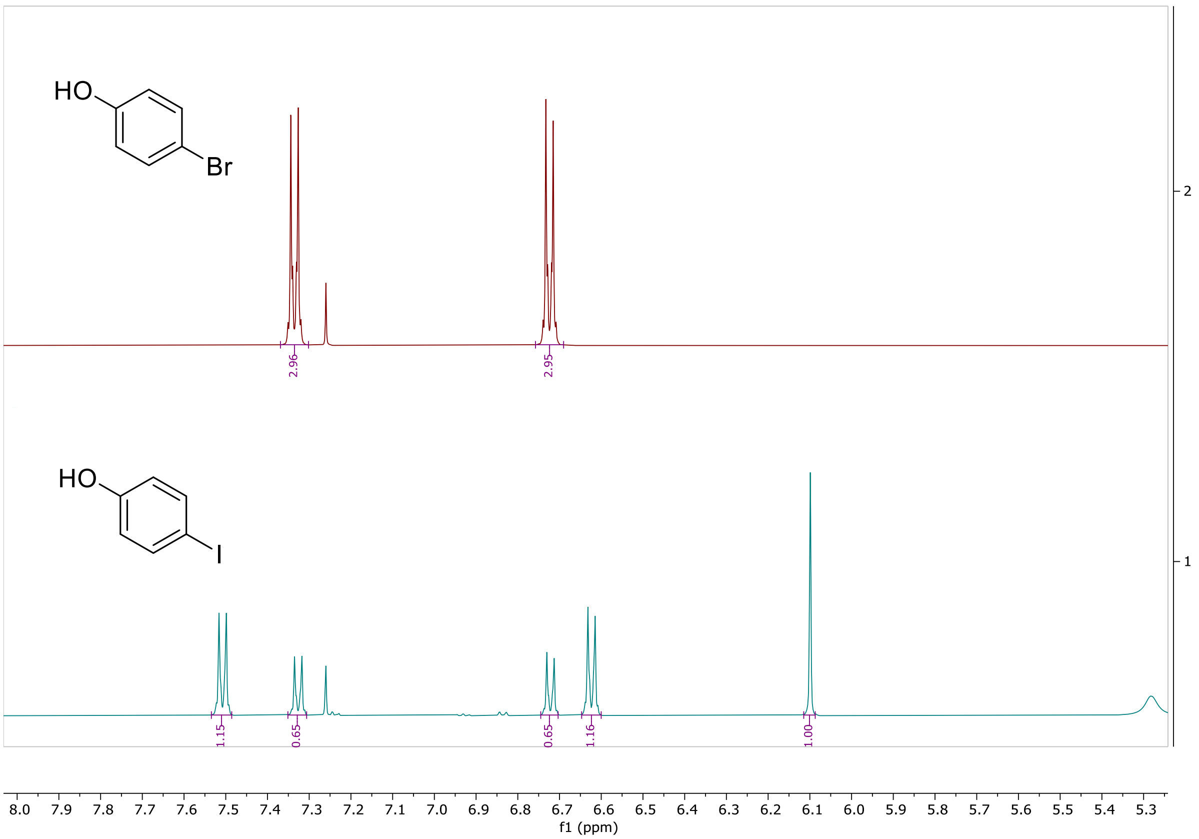

Figure S52. Overlaid ^{1}H NMR spectrum of **4-bromophenol** and **24** in $CDCl_3$

7. References

1 N. Maraš, S. Polanc and M. Kočevar, *Tetrahedron*, 2008, **64**, 11618–11624.

2 A. A. Jalil, N. Kurono and M. Tokuda, *Tetrahedron*, 2002, **58**, 7477–7484.

3 S. Y. Hong and A. T. Radosevich, *J Am Chem Soc*, 2022, **144**, 8902–8907.

4 Z. Qu, X. Chen, S. Zhong, G. J. Deng and H. Huang, *Org Lett*, 2021, **23**, 5349–5353.

5 N. Terzić, J. Konstantinović, M. Tot, J. Burojević, O. Djurković-Djaković, T. Srbljanović Jelena and Štajner, T. Verbić, M. Zlatović, M. Machado, I. S. Albuquerque, M. Prudêncio, R. J. Sciotti, S. Pecic, S. D'Alessandro, D. Taramelli and B. A. \v Solaja, *J. Med. Chem.*, 2016, **59**, 264–281.

6 P. Boehm, T. Martini, Y. H. Lee, B. Cacherat and B. Morandi, *Angew. Chem. Int. Ed Engl.*, 2021, **60**, 17211–17217.

7 P. Chen, K. Luo, X. Yu, X. Yuan, X. Liu, J. Lin and Y. Jin, *Org. Lett.*, 2020, **22**, 6547–6551.

8 N. T. Kadunce and S. E. Reisman, *J. Am. Chem. Soc.*, 2015, **137**, 10480–10483.

9 Z. Cheng, P. Sun, A. Tang and C. Jin Weiwei and Liu, *Org. Lett.*, 2019, **21**, 8925–8929.

10 Y. Li, S. A.-E.-A. Rizvi, D. Hu, A. Sun Danwen and Gao, Y. Zhou, J. Li and X. Jiang, *Angew. Chem. Int. Ed Engl.*, 2019, **58**, 13499–13506.

11 M. H. Keylor, Z. L. Niemeyer and K. L. Sigman Matthew S and Tan, *J. Am. Chem. Soc.*, 2017, **139**, 10613–10616.

12 G. J. P. Perry, J. M. Quibell and I. Panigrahi Adyasha and Larrosa, *J. Am. Chem. Soc.*, 2017, **139**, 11527–11536.

13 Y. Wu, S. Xu, H. Wang, D. Shao, Q. Qi, Y. Lu, L. Ma, J. Zhou, W. Hu, W. Gao and J. Chen, *J. Org. Chem.*, 2021, **86**, 16144–16150.

14 A. Palav, B. Misal and G. Chaturbhuj, *J. Org. Chem.*, 2021, **86**, 12467–12474.

15 R. Mancuso, C. C. Pomelli, F. Malafronte, A. Maner, N. Marino and B. Chiappe Cinzia and Gabriele, *Org. Biomol. Chem.*, 2017, **15**, 4831–4841.

16 J. Ren, M.-C. Jia, F.-H. Du and C. Zhang, *Chin. Chem. Lett.*, 2022, **33**, 4834–4837.

17 D. Shen, C. Miao, S. Wang and W. Xia Chungu and Sun, *Org. Lett.*, 2014, **16**, 1108–1111.

18 C. Li, R. Xu, Q. Song, Z. Mao, J. Li, H. Yang and J. Chen, *Tetrahedron Lett.*, 2022, **98**, 153818.

19 D. Shabashov and O. Daugulis, *J. Am. Chem. Soc.*, 2010, **132**, 3965–3972.

20 L. Qin, B. Hu, K. D. Neumann, K. Linstad Ethan J and McCauley, J. Veness, J. J. Kempinger and S. G. DiMagno, *European J. Org. Chem.*, 2015, **2015**, 5919–5924.

21 L. E. Hofmann, L. Mach and M. R. Heinrich, *J. Org. Chem.*, 2018, **83**, 431–436.

22 K. Yu, H. Zhang, Y. Sheng and Y. Zhu, *Tetrahedron Lett.*, 2020, **61**, 152010.

23 Y. Zhao, H. van Nguyen, L. Male, P. Craven, B. R. Buckley and J. S. Fossey, *Organometallics*, 2018, **37**, 4224–4241.

24 X. Fang, J. Cao, W. Ding, H. Jin, X. Yu and S. Wang, *Org. Lett.*, 2021, **23**, 1228–1233.

25 R. R. Thakore, B. S. Takale, V. Singhania, F. Gallou and B. H. Lipshutz, *ChemCatChem*, 2021, **13**, 212–216.

26 T. Markovic, P. R. D. Murray, B. N. Rocke, A. Shavnya, D. C. Blakemore and M. C. Willis, *J Am Chem Soc*, 2018, **140**, 15916–15923.

27 J. W. Lai, Z. Y. Liu, X. Y. Chen, H. Zhang and H. Y. Liu, *Tetrahedron Lett*, 2020, **61**, 152426.

28 M. Cortes-Clerget, N. Akporji, F. Zhou Jianguang and Gao, P. Guo, M. Parmentier, F. Gallou, J.-Y. Berthon and B. H. Lipshutz, *Nat. Commun.*, 2019, **10**, 2169.

29 S. A. Hudson, K. J. McLean, S. Surade, Y. Q. Yang, D. Leys, A. Ciulli, A. W. Munro and C. Abell, *Angewandte Chemie - International Edition*, 2012, **51**, 9311–9316.

30 S. Dumouchel, F. Mongin, F. Trécourt and G. Quéguiner, *Tetrahedron*, 2003, **59**, 8629–8640.
